# Monoamine Oxidase Inhibitors in Drug Discovery Against Parkinson’s Disease: An Update

**DOI:** 10.3390/ph18101526

**Published:** 2025-10-10

**Authors:** Luana Vergueiro Ribeiro, Larissa Emika Massuda, Vanessa Silva Gontijo, Claudio Viegas

**Affiliations:** PeQuiM—Laboratory of Research in Medicinal Chemistry, Institute of Chemistry, Federal University of Alfenas, Alfenas 37133-840, MG, Brazil; luana.vergueiro@sou.unifal-mg.edu.br (L.V.R.); laahmassuda@gmail.com (L.E.M.); vanessagontijo@yahoo.com.br (V.S.G.)

**Keywords:** neurodegenerative diseases, α-synuclein, Parkinson’s disease, MAO, monoamine oxidase inhibitors

## Abstract

**Background:** Parkinson’s disease (PD) is a progressive neurodegenerative disorder with substantial socioeconomic impact, characterized by the gradual loss of dopaminergic neurons, dopamine deficiency, and pathological processes such as neuroinflammation, oxidative stress, and α-synuclein aggregation. Monoamine oxidases (MAOs) are enzymes responsible for the degradation of neuroactive amines, including dopamine, a neurotransmitter essential for motor, cognitive, and behavioral functions. Among these, MAO-B plays a central role in dopamine metabolism, producing reactive metabolites and oxidative species that contribute to the oxidative stress associated with PD pathophysiology. In this context, MAO-B inhibition has emerged as a promising therapeutic strategy. However, specific limitations, such as motor complications linked to prolonged levodopa use and the adverse effects of currently available MAO inhibitors, remain significant clinical challenges. **Methods:** A comprehensive literature search was conducted using PubMed and SciFinder databases. Keywords such as “MAO inhibitors”, “Parkinson’s pathology,” and “Parkinson’s disease” were combined with Boolean operators (AND, OR, NOT). The search covered publications from 2010 to 2025. **Results:** While previous reviews, particularly those by the groups of Guglielmi and Alborghetti, mainly emphasized the clinical use of MAO-B inhibitors and advances in patents, the present review identified approximately 300 compounds synthesized and evaluated as MAO inhibitors, encompassing diverse chemical classes. Among them, selective MAO-B inhibitors exhibited the greatest pharmacological potential, reinforcing the relevance of this isoform as a strategic target in PD therapy. **Conclusion:** These findings highlight the advances of Medicinal Chemistry in the development of novel MAO-B inhibitors, both as monotherapies for early-stage PD and as adjuvants to levodopa in advanced disease. Collectively, they emphasize the promise of MAO-B inhibitors as candidates for more effective therapeutic interventions in Parkinson’s disease.

## 1. Introduction

Parkinson’s disease (PD) is a chronic progressive disease, affecting about 1% of the world’s population aged over 60 [1]. It is the second most common neurodegenerative disease (ND) [2], with an estimated prevalence ranging from 100 to 300 cases per 100,000 people [3]. Considering the global increase in life expectancy, the prevalence of Parkinson’s disease (PD) is projected to reach 15.6 million cases by 2030 and 25.2 million by 2050, representing a 112% increase compared with epidemiological data from 2021 [4].

PD is characterized by motor changes such as tremors, bradykinesia, rigidity, and postural instability, due to the degeneration of 50–80% of dopaminergic neurons in the substantia nigra (SN). In addition, there are non-motor symptoms that include depression, sleep disturbance, constipation, and anosmia, which usually precede motor symptoms, as well as speech impairment as the disease progresses [2,5]. Despite being a multifactorial disease, its progression is mainly associated with α-synuclein (α-SYN) proteotoxicity and the formation of Lewy’s bodies (LB), which are clusters and deposits of poorly processed α-synuclein fibrils. In turn, the presence of such neurotoxic deposits plays a central role in the alteration of other biochemical pathways associated with the evolution and worsening of the disease, such as oxidative stress (OS), mitochondrial dysfunction, dopamine oxidation, and excitotoxicity [5]. In particular, alteration in the degradation process of catecholamines by monoamine oxidase (MAO) enzyme, whose activity generates neurotoxic products, has been well documented by its contribution to the pathogenesis and symptoms of PD.

MAO exists in two isoforms: MAO-A and MAO-B, which have about 70% homology concerning amino acid sequences (primary structure) and a three-dimensional structure (tertiary structure) [6]. Despite the high degree of homology, both isoforms have different specificities regarding the substrate and distribution in the human body. Although both enzymes degrade dopamine (DpA), noradrenaline, tryptamine, and tyramine in various brain regions, in the SN, degradation of DpA is mainly catalyzed by MAO-B, evidencing the importance of this isoform in the loss of dopaminergic neurons [7]. In addition, it is important to emphasize that the current pharmacological therapy for PD is only symptomatic, aiming at improving dopaminergic signaling and life quality of the patient. Therefore, there are still no effective treatments for slowing the progression of PD, and the few approved drugs have several side effects [8]. Thus, massive investments in research for new drug candidates, with innovative mechanisms of action, which include MAO inhibition, are urgent and should provide important information for the development of more effective and safer medicines. In recent years, medicinal chemists have dedicated intensive efforts to the search for new molecules, preferably capable of operating by multiple mechanisms of action or directed to multiple targets. In this scenario, MAOs have shown great relevance as potential targets against the complex PD pathophysiology, justifying the importance of gathering the most recent information in the literature to contribute to the design, optimization, and development of novel disease-modifying drug prototype candidates.

Previous reviews have substantially contributed to the field, such as the work by Alborghetti et al. (2019), which addressed aspects of the laboratory and clinical development of a series of MAO-B inhibitors [9], and more recently, Guglielmi and colleagues, who provided an overview of MAO-A and MAO-B inhibitors patented between 2018 and 2021, with greater emphasis on chemistry and less focus on clinical applications [10]. The present work, however, aims to provide a comprehensive review of MAO inhibitors reported over the past fifteen years, encompassing not only the results of pharmacological assays but also detailed chemical structures. The compounds were organized into distinct chemical groups, and, finally, a final selection of the most promising inhibitors was constructed based on their selectivity index, offering a critical perspective that complements and expands upon the approaches of previous reviews.

### 1.1. General Pathophysiological Aspects of Parkinson’s Disease

**Figure 1:**
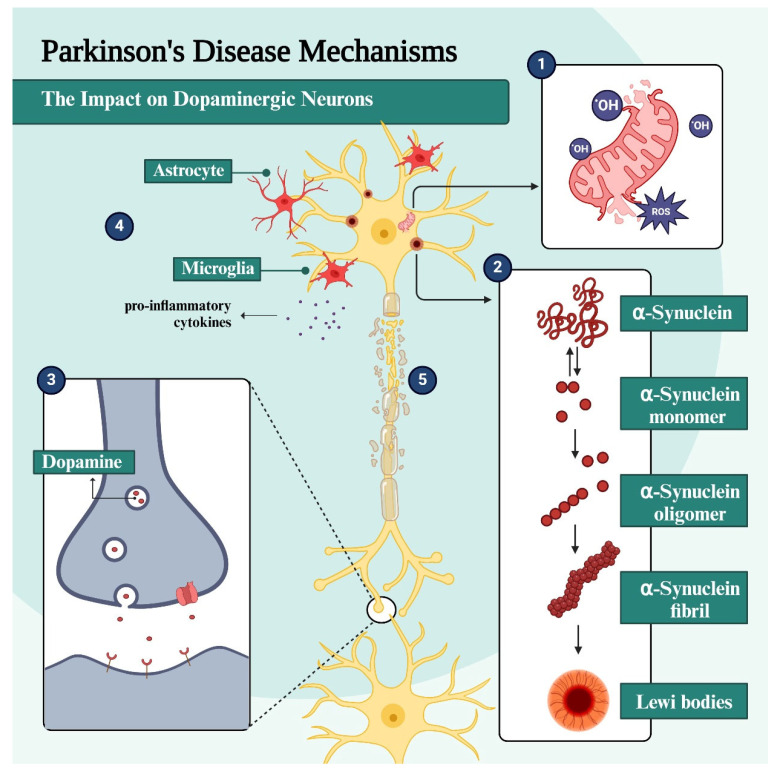
Multifactorial aspects related to PD physiopathology. (**1**) Mitochondrial dysfunction and excessive generation of reactive oxygen species (ROS); (**2**) misfolding of α-synuclein, starting from functional monomers, progressing to toxic oligomers, insoluble fibrils, and resulting in the formation of Lewi’s bodies; (**3**) Dopaminergic deficit triggered by oxidative stress and inflammation, besides the accumulation of Lewy’s bodies; (**4**) Inflammatory process mediated by the activation of astrocytes and microglia, culminating in the release of pro-inflammatory cytokines that amplify the neuroinflammatory picture; (**5**) interconnected cycle of proteotoxicity, oxidative stress and inflammation affecting dopaminergic neuron and its degeneration.

PD is a progressive ND characterized by the damage and death of dopaminergic neurons present mainly in the SN and the basal ganglia [11,12]. DpA is a neurotransmitter closely involved in motor and executor control, and its continuous release in the dorsal striatum is essential for the regulation of movements [13]. During the synaptic process, degradation of DpA is mainly mediated by MAO-B, after the presynaptic reuptake process [14]. In addition to the characteristic symptoms of PD, some non-motor manifestations may slowly be preceded by years, including depression, hyposmia, sleep disturbances, and constipation [13]. This complex mosaic of symptoms is thought to be due to the multifactorial picture associated with neuroinflammation, mitochondrial dysfunction, OS in the central nervous system (CNS), gradual accumulation and deposition of α-synuclein fibrils, and formation of LB, as well as massive loss of dopaminergic neurons and other cellular and biochemical dysfunctions that are not yet well understood (Figure 1) [15].

Among all neuronal cells in the SN, which make up the nigrostriatal pathway, dopaminergic neurons seem to have the greatest vulnerability, as opposed to those that make up the mesolimbic pathway [11]. Neurons affected by LB formation undergo morphological and functional changes that include elongated, thin axons with a thin or absent myelin sheath, which could increase their susceptibility to the deposition of α-synuclein aggregates [16]. Moreover, DpA is also pointed out as another cause of dopaminergic neuron vulnerability, since the oxidation of this neurotransmitter generates neuromelanin (NM) and 5,6-di-hydroxy-indole, in addition to the overproduction of reactive oxygen species (ROS). Notably, dopaminergic neurons in the SN are characterized by the presence of NM [17] and are more susceptible to OS because, in addition to having long and myelinated axons, they still require high energy demand and have a pacemaker activity, which leads to transient levels of cytosolic Ca^2+^ and higher levels of DpA, culminating in the production of toxic metabolites that activate and exacerbate OS [11].

### 1.2. The Role of α-Synuclein and Lewy Bodies in PD Pathogenesis

Proteotoxicity is one of the most pronounced hallmarks of ND, which is caused by the accumulation of misfolded or poorly processed insoluble protein fragments, such as α-synuclein in PD. In the pathological condition of PD, this protein changes from a monomeric to an oligomeric form, which is less soluble and more prone to aggregation and deposition in the neuronal cytosol. Once deposited, α-SYN aggregates are the main components of LB, commonly observed in PD patients [18]. This accumulation of poorly processed proteins, with a misfolded structure, progresses in a predictable manner and is known as the “Braak stage”. Initially, it is observed in the dorsal motor nucleus of the glossopharyngeal and vagus nerves and the anterior olfactory nucleus and later migrates to the brainstem and neocortex [18,19].

In a physiological state, α-synuclein is in its monomeric form and interacts with ATP synthase, being able to regulate this enzyme and improve the efficiency of ATP synthesis [20]. It is an abundant neuronal protein in pre-synaptic terminals, and by a not fully understood process, pathogenic conditions result from alterations in its biosynthesis and degradation, or genetic and epigenetic factors, such as mutations in the SNCA gene (gene encoding α-synuclein) and alterations in the lysosomal degradation pathway [14,19,20]. The oligomeric form of α-SYN induces oxidation of the ATP synthase subunit and lipid peroxidation in the mitochondria. Due to the oxidation of ATP synthase, excessive production of ROS occurs, damaging lipids, proteins, and other endogenous molecules. On the other hand, mitochondrial lipid peroxidation increases the likelihood of the permeability transition pore (PTP) opening, which causes swelling and, subsequently, cell death [20]. In addition, the depolarization of the mitochondrial membrane induces the release of cytochrome C and mitochondrial fragmentation, which is associated with OS, leading to the accumulation of oxidized DpA, lysosomal dysfunction, and, in turn, a greater accumulation of α-SYN [19]. In this cycle of physiological changes, the accumulation of α-SYN neurofibrils can incite diverse neurotoxic effects and also favor the accumulation of more misfolded protein aggregates [16]. Studies raise the possibility of positive pathogenic feedback between the lysosome and mitochondria since lysosomal dysfunction leads to deficits in mitophagy, i.e., the accumulation of dysfunctional mitochondria [21].

As in other NDs, aging is a risk factor for PD development and is linked to a reduction in the functioning of the lysosomal autophagic system (LAS) and ubiquitin-proteasome systems, which are responsible for the homeostasis of intracellular α-synuclein. This association is confirmed by experimental data showing increased levels of α-synuclein in dopaminergic neurons in the SN during normal aging, corroborating that inhibition of any of these degradation systems can lead to elevated levels of this protein [14].

### 1.3. Oxidative Stress and Neuroinflammation in PD

In a healthy physiological system, ROS and reactive nitrogen species (RNS) are normally produced in low concentrations and are useful in regulating diverse cell functions. However, under pathological conditions these highly reactive radical species become harmful agents when generated in an exacerbated and uncontrolled manner, overloading the antioxidant defense system and leading to OS which, as already mentioned, triggers toxic effects on cells [18]. The brain is one of the organs with the highest oxygen demand and, therefore, the presence of these free radicals without a detoxifying system makes it more susceptible to oxidative damage [18,19]. Due to their high reactivity, radical species can cause functional changes in cells by interacting and causing modifications to proteins and DNA, whose attempt to repair triggers an inflammatory process in the affected region, which in the case of a brain with PD is more prominent in the SN [12].

In the context of neurodegeneration, 1-methyl-4-phenyl-1,2,3,6-tetrahydropyridine (MPTP) is the precursor form of the neurotoxin MPP+, which originates from the degradation of MPTP by the action of MAO-B, which can inhibit mitochondrial respiration and an increase in ROS [8]. ROS can cause mutation in mitochondrial DNA and oxidation of α-SYN, parkin, and proteasome proteins in the CNS, leading to increased formation of aggregates of the respective oxidized and malformed protein fragments, triggering neuroinflammation. In addition, the activation of K^+^ channels, concomitant with the inactivation of nicotinic receptors, leads to impaired release of DpA and acetylcholine (ACh), respectively, resulting in the motor impairment hallmark of PD [12]. Several studies have shown that microglia activation in the SN and striatum of PD brains and animal models is associated with an exacerbation in the levels of pro-inflammatory cytokines in the cerebrospinal fluid (CSF) and basal ganglia. In addition, there is evidence of a role for the complement system in this pathogenesis, since high levels of characteristic proteins of this system are also observed in LB. In vitro studies have shown that α-synuclein in different forms (nitrated or aggregated, for example) can induce the activation of microglia, which occasionally generate more toxic cytotoxic products [18].

### 1.4. Mitochondrial Dysfunction in PD Pathogenesis

Post-mortem analysis of PD brains revealed defects in complex I (CI) of the mitochondrial respiratory chain in SN, frontal cortex, and peripheral tissues, such as platelets and skeletal muscle. This evidence supports the hypothesis that mitochondrial dysfunction is one of the factors triggering PD neuropathogenesis [8,22]. More recent studies on the PD pathophysiology have confirmed that factors such as MPTP degradation and rotenone inhibit the functioning of the mitochondrial respiratory chain, due to damage to the mitochondrial CI, and the consequent failure in ATP generation, increased expression of pro-apoptotic pathways and, subsequently, cell death [23].

Mitochondria play a central role in producing energy in the form of ATP through oxidative phosphorylation, as well as helping to regulate cell death, calcium homeostasis, formation and transport of the Fe-S complex, and acting in the control of cell division and growth. This organelle is composed of a lipid bilayer, with an inner and outer membrane, and the interstitial space contains important units for oxidative phosphorylation. Due to the location of mitochondrial DNA, close to the electron chain, it is more exposed to damage from free radicals generated there, which can lead to altered genetic material related to PD. In addition, during the oxidative phosphorylation process, some electrons can escape from the respiratory chain, especially from complexes I and III, which react with molecular oxygen and form superoxide (O^2−^). In non-pathological conditions, this production occurs in very low concentrations, so that the mitochondrial antioxidant system can neutralize and remove ROS, such as the action of manganese superoxide dismutase (MnSOD) and glutathione (GSH). This evidence suggests that an imbalance in this electron leak, and the consequent excessive formation of O^2−^, is one of the main factors leading to cell death in PD [22]. It has been experimentally proven that the peroxynitrite formed by the reaction of ROS with nitric oxide (NO) and tyrosine nitrate residues in proteins is capable of damaging protein integrity and leading to cell death. Nitrotyrosine residues have been observed in LB from PD patients, suggesting the possibility that when protein nitration occurs, the risk of developing PD is increased. In other studies, it has been suggested that ROS or reactive quinones produced by the oxidation of DpA, either spontaneously or through the action of MAO, could have an inhibitory effect on respiratory chain proteins. It has been experimentally proven that DpA could inhibit complexes I and IV, suggesting that this occurs through the action of quinones and ROS [22].

### 1.5. Glutamate Production and Excitotoxicity in PD

Mitochondrial dysfunction is still considered one of the causes of excitotoxicity because it reduces intracellular ATP levels, causing partial neuronal depolarization by decreasing Na^+^/K^+^-ATPase activity and, consequently, increasing Ca^2+^ influx [22]. Concomitantly to the dopaminergic neuron death, there is an overactivation of the subthalamic nucleus, which leads to an increase in the release of glutamate in the SN region. Therefore, higher levels of glutamate lead to excessive activation of *N*-methyl-*D*-aspartate (NMDA) receptors, which are abundant in the SN [12]. Allied with this, increased Ca^2+^ influx generates a response from the ion transporter to high levels of extracellular Na^+^, leading to an accumulation of Ca^2+^ in the mitochondria, affecting ATP synthesis, as well as an overproduction of ROS that contributes to oxidative damage [22].

In vivo studies of the effects of NMDA antagonists in MPTP-induced PD-like conditions revealed a protective effect against the loss of SN dopaminergic neurons. However, these antagonists have limited use due to their low potency and poor tolerance [18]. In addition to the excessive release of glutamate, the reduction in its reuptake can also result in its accumulation in the synaptic cleft, reinforcing overactivation of NMDA receptors and neuronal excitotoxicity. Overstimulation of the NMDA receptors induces various neurotoxic effects, such as increased production of NO, ROS, RNS, and disruption of homeostasis, generating mitochondrial dysfunction, showing that glutamate-induced excitotoxicity is a relevant factor in dopaminergic neuron death in the SN [15].

### 1.6. Monoamine Oxidases: Functional Aspects and Its Relevance in the PD Pathogenesis

Monoamine oxidases (MAOs) are enzymes specialized in the degradation of neuroactive amines, but the two isoforms have substrate selectivity [24]. MAO-A is mainly involved in the degradation of norepinephrine and serotonin, while MAO-B is responsible for degrading most DpA and 2-phenylethylamine, among other monoamine compounds [25]. In addition, both isoforms are found in the outer mitochondrial membrane and are distributed throughout peripheral organs and in the brain. MAO-A predominates in catecholaminergic neurons, while MAO-B is predominant in serotoninergic and histaminergic neurons, besides in astrocytes. However, in the human brain, MAO-B activity predominates over MAO-A by more than 80%. In peripheral tissues, MAO-B is largely localized in platelets and lymphocytes, and MAO-A is especially abundant in fibroblasts and the placenta. Notably, one isoform is expressed in cells that contain the substrate of the other isoform, which is suggestive of possible protection of oxidases in their host cells by also degrading other substrates [26]. The main structural and functional differences between MAO-A and MAO-B are summarized in Table 1.

The degradation of monoamines by the action of MAOs initially involves the formation of aldehyde intermediates which are subsequently converted into their respective carboxylic acids by the action of aldehyde dehydrogenase (ALDH) or can also be converted into glycols or alcohols by aldehyde reductase (ALR) [27]. These degradation reactions generate H_2_O_2_, NH_3_, and aldehydes, with H_2_O_2_ playing a relevant role when it comes to the formation of mitochondrial ROS, which could lead to apoptosis [28]. Particularly, dopaminergic neurons in the SN are more exposed to ROS resulting from metabolites released in the catecholamine degradation [29]. The levels of neurotransmitters such as serotonin and DpA play a fundamental role in regulating areas such as cognition, motor functions, endocrine regulation, behavior, and cognition, which may explain the relationship of the monoaminergic system with various NDs, such as PD [30].

DpA is a fundamental neurotransmitter, responsible for modulating various functions, including behavior, decision-making, mood, and aggression control, reproductive behavior, learning, and memory [31]. It is a reactive molecule that is stored in neuronal synaptic vesicles, since in the cytosol it could be spontaneously oxidized by reactions particularly catalyzed by ions, such as Fe^2+^/Fe^3+^, and can be inhibited by antioxidants such as GSH [32]. Despite being essential, the exacerbated degradation of DpA induces OS, both through the non-enzymatic and enzymatic pathways. The non-enzymatic pathway involves the formation of neurotoxic semiquinone and *ortho*-quinone byproducts, that once polymerized could form NM and ROS. The enzymatic pathway, mediated by MAO-A and MAO-B, is the main pathway for DpA degradation, generating 3,4-dihydroxyphenylacetaldehyde (DOPAL) and H_2_O_2_. In turn, H_2_O_2_ is highly responsible for OS, because although it is not considerably reactive, in the presence of high concentrations of iron ions in SN, it can be converted into other highly reactive metabolites [33]. In the context of oxidative reactions, oxidation of DpA with the loss of 1 electron can interfere with its storage and generate oxidative proteins and changes in genetic material. Moreover, oxidation with the loss of 2 electrons generates *ortho*-quinone, which can react with biological nucleophiles, which are capable of redox cycling and depleting cellular antioxidants [34].

Considering all the above mentioned data, inhibition of MAOs, especially MAO-B, has attracted special attention as a promising target for drug discovery and improved therapeutic strategies.

**Table 1:** Comparison between MAO-A and MAO-B isoforms: localization, substrates, physiological functions, risks, and clinical applications.

Characteristic	MAO-A	MAO-B
Tissue Location	Mainly expressed in peripheral tissues (liver, kidney, and heart) and brain (cortex and hippocampus) [26]	Mainly expressed in brain (striatum, thalamus, pale globe, and substantia nigra), platelets, and glia [35]
Cell distribution	Catecholaminergic neurons, hepatocytes, and cardiomyocytes [26]	Serotoninergic and histaminergic neurons, astrocytes, platelets, and renal cells [36]
Primary Substrates	Serotonin, norepinephrine, adrenaline, dopamine (pre-synaptic neurons), and tyramine [25]	Dopamine (synaptic cleft and glial cells), phenylethylamine, benzylamine, and tyramine [35]
Physiological Role	Metabolism of peripheral monoamines and regulation of humor and anxiety (via serotonin and noradrenaline) [37]	Control of striatal dopaminergic pathway, motor function modulation, metabolism of phenylamine, and oxidative stress modulation in glial cells [35]
Inhibition-related Risk	Tyramine accumulation (Cheese effect) and serotoninergic syndrome [37]	Low risk of cheese effect, which is potentialized by high doses or low selectivity [36]
Clinical uses of inhibitors	Antidepressive and anxiolytics [37]	Potential monotherapy for early stages PD or as adjuvant to levodopa on advanced stages [35]

### 1.7. Current Available Drugs for PD Therapy

**Figure 2:**
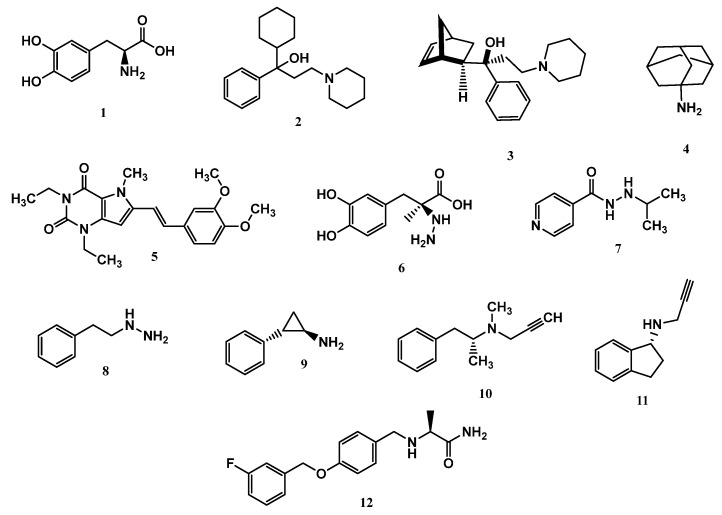
Chemical Structures of levodopa (**1**), trihexyphenidyl (**2**), biperiden (**3**), amantadine (**4**), istradefylline (**5**), carbidopa (**6**), iproniazid (**7**), phenelzine (**8**), tranylcypromine (**9**), selegiline (**10**), rasagiline (**11**), and safinamide (**12**), the main approved drugs for PD therapy.

Given the multifactorial nature of PD, the pharmacological options currently available cover different therapeutic targets, but only in a symptomatic manner, aiming to ameliorate life quality, but not being capable of slowing the disease’s progress. These include levodopa (**1**, Figure 2), anticholinergics, anti-glutamatergics, MAO inhibitors, DpA agonists, catechol-*O*-methyltransferase (COMT) inhibitors, and adenosine A2 receptor antagonists [38]. Levodopa is a precursor of DpA, and its clinical use is based on pharmacokinetic purposes since levodopa can cross the blood–brain barrier (BBB), but not DpA. It is therefore a prodrug and is the main and first choice therapeutic alternative for the symptomatic treatment of PD. However, despite its clinical efficacy, its prolonged use can cause levodopa-induced dyskinesia, as well as other adverse effects such as nausea and drowsiness, due to the action of DpA β-hydroxylase (DBH), a peripheral enzyme that catalyzes the conversion of DpA into *nor*-epinephrine and noradrenaline [39].

Anticholinergic or antimuscarinic drugs, represented by trihexyphenidyl (**2**) and biperiden (**3**, Figure 2), in turn, are not used as the first option in PD clinics, due to their low efficacy and adverse side effects, including memory loss, confusion, constipation, urinary retention, and dry mouth. However, in young patients or the early disease stages, it is still prescribed [40]. The main representative of the anti-glutamatergic drug class is amantadine (**4**), whose mechanism of action leads to an improvement in the release of DpA and inhibition of its reuptake, through changes in glutamine receptor affinity and blockade of glutamate NMDA receptors, to normalize glutamatergic activity. In addition, these drugs can reduce the effect of levodopa-induced dyskinesia, but their side effects include hallucinations, peripheral edema, and dizziness [38,41]. DpA agonists are drugs capable of binding to endogenous DpA receptors in the absence or decrease of DpA. They are currently used as a first-line symptomatic treatment for patients aged over 60 and are capable of delaying motor complications and dyskinesia, but their side effects include constipation, nausea, headaches, sleep disorders, and hallucinations [42].

The COMT enzyme is another pharmacological target since its inhibition leads to an increase in DpA levels. This enzyme is responsible for metabolizing catecholamines in the dopaminergic system; besides DpA, it also degrades levodopa, blocking the biosynthesis of DpA. Although generally well tolerated, COMT inhibitors have significant side effects, which include hepatotoxicity, levodopa-induced dyskinesia, nausea, postural hypotension, diarrhea, and orange-colored urine [38,43].

Adenosine A2 receptor antagonists facilitate the transmission of DpA in the prefrontal cortex, which may be related to cognitive function. Istradefylline (**5**, Figure 2) is an example of an A2 receptor antagonist drug and can be used in association with levodopa or carbidopa (**6**, Figure 2), leading to improvement in motor fluctuations related to levodopa. Usually, istradefylline is well tolerated, and its side effects include increased dyskinesia, dizziness, constipation, nausea, hallucinations, and insomnia [38,44].

The discovery of MAO inhibitors dates back to 1951, when clinical trials with iproniazid (**7**, Figure 2), a drug used to treat tuberculosis, revealed euphoria as one of its side effects. Further studies showed that the mechanism of action of iproniazid involved MAO inhibition, increasing the bioavailability of unmetabolized endogenous monoamines [27]. Subsequent studies resulted in the first generation of MAO inhibitors, represented by a group of non-selective and irreversible drugs such as phenelzine (**8**) and tranylcypromine (**9**, Figure 2). The administration of these drugs resulted in the “cheese effect”, which was so-called because of the rise in blood pressure after eating foods containing tyramine, which is mainly present in cheese and wine. The next generation of MAO inhibitors was based on the discovery of the two MAO isoforms, represented by selegiline (**10**), a selective MAO-B inhibitor. The increased selectivity for MAO-B avoids the “cheese effect” caused by MAO-A inhibition in the gut. Contradictorily, the third generation consists of MAO-A inhibitor drugs aimed at treating depression, one of which is resveratrol, a natural polyphenol abundant in red grapes and various types of almonds [27,30]. However, to date, non-selective MAO inhibitors usually show low affinities for each enzyme isoform, leading to important adverse effects. In addition, given the mechanism of action based on an irreversible inhibition mode, these drugs have a broad spectrum of intrinsic toxicity that includes orthostatic hypotension, hepatotoxicity, and hypertensive crises [45].

Among the drugs currently used to treat PD, whose mechanism of action involves MAO inhibition, selegiline (**10**, Deprenyl, Figure 2), rasagiline (**11**, Figure 2), and safinamide (**12**, Figure 2) stand out. Selegiline is an irreversible MAO-B inhibitor and is used for the symptomatic treatment of motor dysfunction, both in the early and late stages of PD. More recently, rasagiline was introduced into the market as another irreversible MAO-B inhibitor. However, both selegiline and rasagiline generate neurotoxic metabolites [46]. One of the most recent advances is represented by safinamide, a selective MAO-B inhibitor approved by the FDA in 2017, which acts as a reversible MAO-B inhibitor capable of preventing the reuptake of DpA and modulating the release of glutamate, mitigating excitotoxicity [47].

## 2. Methodology

In this work, the most recent contributions of MedChem are compiled and discussed; the international community, whether in Academia or the Pharmaceutical Industry, has elected MAO-A and MAO-B as molecular targets of interest in the search for new bioactive chemical entities with potential application in the PD therapy. The methodology used was based on data collection from PubMed and SciFinder platforms. The keywords were combined by using Boolean operators (AND, OR, NOT) resulting in “MAO inhibitors AND Parkinson’s disease”, “Parkinson’s pathophysiology”, “Parkinson pathology”, and “MAO AND Parkinson’s disease”, covering the period from 2010 to 2025. Studies dealing with natural products, pharmacology, and those that were not electronically available were excluded. Thus, the objective was to systematically construct a review that can contribute to the area of Medicinal Chemistry, especially dedicated to the design of new prototype candidate drugs against PD.

## 3. Recent Advances in the Search for MAO Inhibitors as Drug Candidates for PD

### 3.1. Selective MAO Inhibitors

#### 3.1.1. Indazole and Indole/Melatonin-like Inhibitors

**Figure 3:**
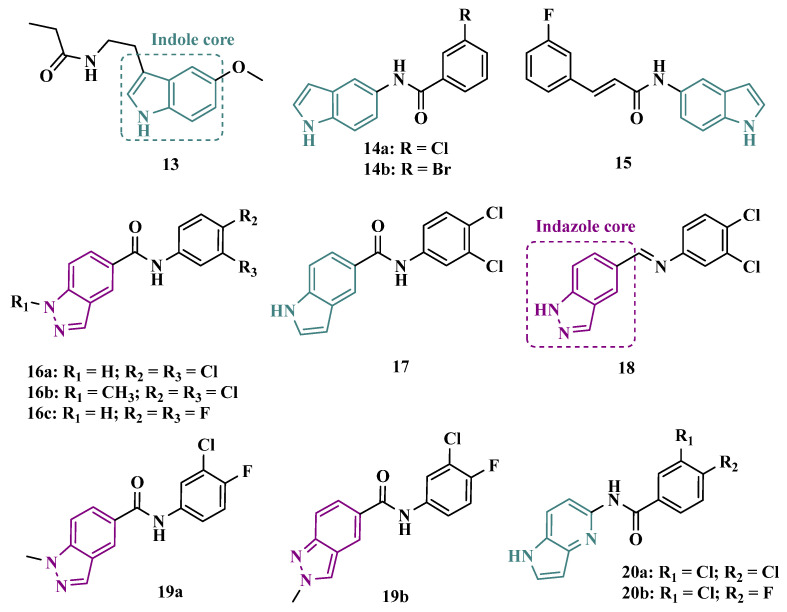
Chemical structures of melatonin **13** and its derivatives **14a** and **14b**, *N*-(1*H*-indol-5-yl)aryl-acrylamide derivative **15**, indazole-5-carboxamine derivatives **16a**–**c**, **18**, indole-5-carboxamide **17**, and the most active N-alkyl-indazol-5-carboxamide derivatives **19a**, **19b**, and pyrrole-pyridin-5-yl)benzamide derivatives **20a** and **20b**.

Melatonin (**13**, Figure 3) is a substance found in most living beings and has several essential properties such as antioxidant, oxygen-free radical scavenger, anti-inflammatory, and neuroprotective, among others. In addition, the age-related decline of its availability has been associated with the progression of NDs, especially PD and Alzheimer’s disease. Experimental data indicate that the melatonin potential against ROS is due to the presence of the electron-rich indole system. Based on this data and on previous studies with indole derivatives, in which excellent MAO-B inhibitors were obtained, Elkamhawy and co-workers synthesized new melatonin analogues (Figure 3) containing diverse aromatic functional groups linked to the position 5 of the indole ring via an amide spacer, aiming at obtaining MAO-B inhibitors against OS. In silico studies suggested good solubility, adequate BBB permeability, good gastrointestinal absorption and higher selectivity index (SI > 50) for most of the compounds, in comparison to rasagiline. The most promising neuroprotective MAO-B selective inhibitors **14a** (IC_50 MAO-B_ = 1.41 μM) and **14b** (IC_50 MAO-B_ = 0.91 μM), did not show significant cytotoxicity towards PC12 cells, being capable of reversing 6-OHDA and rotenone-induced OS in PC12 cells by increasing the expression of HO-1 and inducing the nuclear translocation of the transcriptive factor Nrf2 in a dose-dependent manner [48].

In previous studies, the Duarte group reported a series of 2-(1H-indol-3-yl)ethan-1-amine derivatives with selective MAO-B inhibition and significant ability to reduce neuronal death in in vitro and ex vivo models through ROS reduction. However, these inhibitors exhibited considerable cellular toxicity, as well as compromised stability and limited blood–brain barrier (BBB) permeability [49]. Subsequently, aiming to improve the obtained inhibitors, Duarte conducted an optimization campaign, identifying new N-(1H-indol-5-yl)aryl-acrylamide derivatives that generally displayed high MAO-B inhibitory potency, selectivity, and Nrf2 induction, while achieving favorable pharmacokinetic profiles. Derivative **15** (Figure 3) was identified as the most potent MAO-B inhibitor, with an IC_50_ of 3.61 μM and reversible inhibition. Moreover, compound **15** exhibited strong anti-inflammatory activity in LPS-stimulated BV2 cells, neuroprotective effects against 6-OHDA—restoring 89.7% of cell viability—and protection in the rotenone/oligomycin A model. Finally, the derivative also demonstrated high hepatic microsomal stability and favorable pharmacokinetics in mice, making it a promising candidate [50].

The indole nucleus has been widely studied and considered a privileged structure in MedChem for its synthetic versatility and its pharmacophoric contribution related to various pharmacological properties such as anticancer, anti-inflammatory, neuroprotective, and antimicrobial [51,52]. Many of its derivatives are capable of influencing the neurotransmitter serotonin release/reuptake, as well as having neuroprotective action, by modulating OS [53]. On the other hand, the indazole core, defined by Emil Fisher as a pyrazole ring fused to a benzene ring, a bioisoster of the indole system, is an essential structure for some bioactive compounds and is a common structural fragment in at least 43 current clinical candidates or approved drugs. Indazole compounds substituted with different functionalities have demonstrated diverse biological properties, such as anti-inflammatory, antibacterial, anti-HIV, antiarrhythmic, antifungal, and antitumor, as well as potential inhibition of MAOs [54]. In this regard, Tzvetkov and co-workers synthesized and evaluated new indazole-5-carboxamide and indol-5-carboxamide derivatives (Figure 3), which were assessed in vitro for their ability to inhibit human and murine MAOs. Most compounds showed selectivity for MAO-B, with derivatives **16a**, **16b**, **17**, and **18** standing out with IC_50_ values of 0.59, 0.39, 0.23, and 0.6 nM, respectively. Further studies with **16a** have shown that compounds of the indazole-5-carboxamide type were able to inhibit MAO-B in a reversible and competitive mode [55]. Aiming at structural optimization and following their previous studies, Tzvetkov’s group synthesized another set of indazole-5-carboxamide-*N*-alkylated derivatives (Figure 3). Biological evaluation against rat and human MAO, revealed **19a** (IC_50 hMAO-B_ = 0.66 nM) as a nanomolar selective MAO-B inhibitor, followed by the selective, but less potent, analogue **19b** (IC_50_ = 562 and 8.08 nM, for MAO-A and MAO-B, respectively), with high oral absorption and BBB permeability [56]. Next, still inspired by the structure of indazole pharmacophore, the same group synthesized a novel series of (pyrrolo-pyridin-5-yl)benzamide derivatives (Figure 3), leading to the identification of the most promising compounds **20a** (IC_50 hMAO-B_ = 1.11 nM, IC_50 rMAO-_B = 4.20 nM) and **20b** (IC_50 hMAO-B_ = 3.27 nM, IC_50 rMAO-B_ = 4.90 nM), as highly potent, reversible and competitive MAO-B inhibitors, with good druggability properties, including adequate BBB permeability. In addition, compound **20a** exhibited neuroprotective capacity in cortical neurons and a neurovascular unit cell model, as well as inducing neural network growth [57].

**Figure 4:**
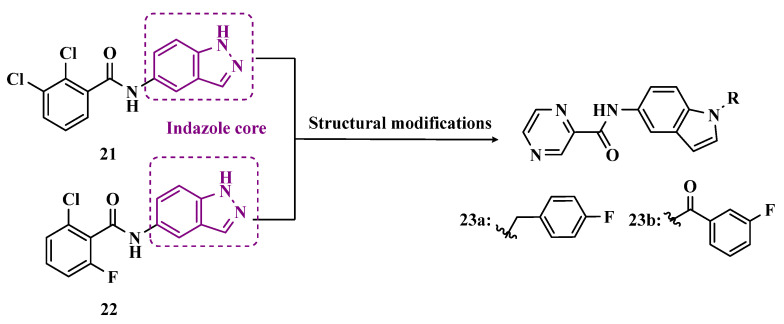
Structures of indazole analogues **21**, **22**, and their improved indole derivatives **23a** and **23b**.

To develop indole-based compounds capable of acting as MAO-B inhibitors, Elkamhawy and co-workers proposed further structural modifications in the Tzvetkov’s indazole project [55], leading to the new scaffolds **21** and **22** (Figure 4). As a result of the biological evaluation, derivatives **23a** (IC_50_ = 1.65 µM) and **23b** (IC_50_ = 0.78 µM) stood out, revealing their reversible and competitive ability to inhibit MAO-B, with good selectivity indices (SI > 60 and 120, respectively) [58]. 

**Figure 5:**
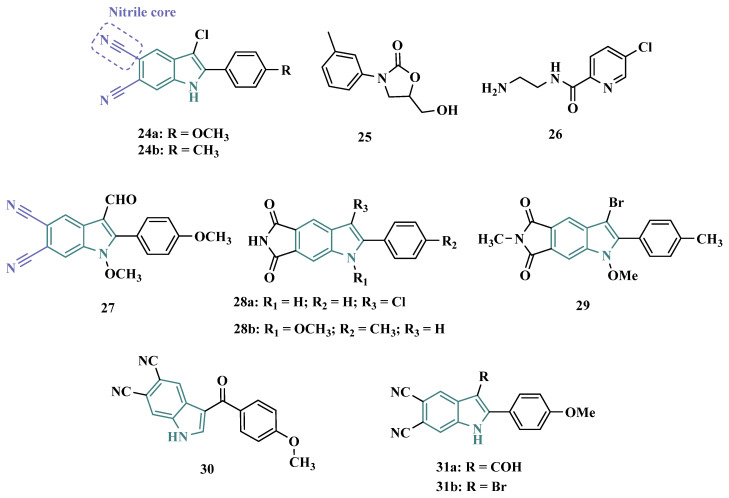
Chemical structures of indole-5,6-dicarbonitrile analogues **24a**, **24b**, **27**, and **30**, MAO inhibitors toloxatone **25** and lazabemide **26**, pyrrole[3,4-f]-indole-5,7-dione derivatives **28a**, **28b**, **29**, and compounds **31a** and **31b**, with selective inhibitory activity of MAO-A and MAO-B.

Based on the fact that phthalonitriles and benzonitriles have also been reported as other chemical classes of compounds with interesting inhibitory activity against MAO, with the nitrile functionality apparently playing a role in the enzyme’s inhibitory potency, Chirkova et al. proposed a novel indole-5,6-dicarbonitrile scaffold (Figure 5) to investigate their ability to inhibit MAO. Biological data evidenced compounds **24a** (IC_50 MAO-A_ = 0.004 µM; MAO-B = 0.020 µM) and **24b** (IC_50 MAO-A_ = 0.014 µM; MAO-B = 0.017 µM), with no selectivity but much more potent than the reference inhibitors toloxatone (**25**, IC_50 MAO-A_ = 3.92 µM) and lazabemide (**26**, IC_50 MAO-B_ = 0.091 µM, Figure 5). Further studies revealed a reversible and competitive mode of inhibition of **24b** for both MAO isoforms [59].

Subsequently, the same group explored other indole derivatives, including indole-5,6-dicarbonitrile, indole-5,6-dicarboxylic acid, and pyrrolo [3,4-f]indole-5,7-dione (Figure 5), aiming to evaluate their MAO inhibitory potential. Altogether, biological data revealed indole-5,6-dicarbonitrile derivatives as specific MAO-A inhibitors, and compound **27** was identified as the most outstanding MAO-A inhibitor with an IC_50_ value of 0.147 µM). The pyrrolo[3,4-f]indole-5,7-dione series also showed promising results, especially for compounds **28a** and **28b**, which stood out as a selective MAO-A inhibitor (IC_50_ = 0.25 µM) and a selective MAO-B inhibitor (IC_50_ = 0.581 µM), respectively [53].

In another approach, Chirkova’s group synthesized and evaluated a series of pyrrolo[3,4-f]indole-5,7-dione and indole-5,6-dicarbonitrile derivatives (Figure 5) that were tested in parallel, revealing that the pyrrolo[3,4-f]indole-5,7-dione derivatives, represented by compound **29**, were more potent and selective in the inhibition of MAO-A than the dicarbonitrile derivatives, represented by **30** (IC_50_ values of 0.813 μM for MAO-A and 0.532 μM for MAO-B, respectively). Compound **29** was identified as the most potent, but non-selective inhibitor of MAO-A/B, with IC_50_ values of 0.023 and 0.178 μM, respectively, with comparable potency to the reference MAO-A inhibitor **25** (IC_50_ = 3.92 μM) and MAO-B **26** (IC_50_ = 0.091 μM) [60].

Seeking to optimize the pharmacological profile and structural patterns of the hit compounds from previous studies [53,59,60], Chirkova’s group synthesized a homologous series of pyrrolo[3,4-f]indole-5,7-dione and indole-5,6-dicarbonitrile architecture. The best MAO inhibitory profile was identified for compounds **31a** (IC_50_ = 6 nM, Figure 5) and its bromide analogue **31b** (IC_50_ = 58 nM), which reached a selective and nanomolar range of potency for MAO-A and MAO-B, respectively [61].

**Figure 6:**
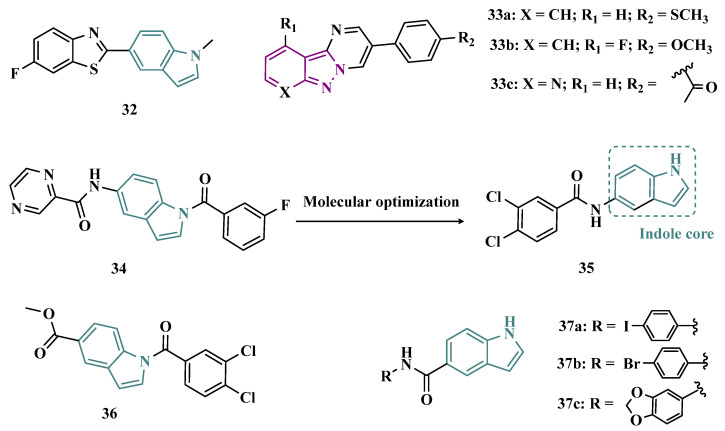
Chemical structures of derivative **32**, the new promising pyrimido[1,2-b]indazole analogues **33a**, **33b** and **33c**, the indole prototype **34** and its optimized most potent and selective MAO-B inhibitor derivatives **35**, **36** and **37a**–**c**.

Studies carried out by Nam et al. with thiazolopyridine and oxazolopyridine derivatives as potential MAO-B inhibitors revealed their low metabolic stability. To optimize their pharmacokinetic profile and MAO-B inhibitory activity, new derivatives with a benzothiazole and benzoxazole motif, containing an indole subunit were then designed. Pharmacological studies evidenced compound **32** (IC_50_ = 28 nM, SI > 357, Figure 6), as a hit nanomolar and selective inhibitor, with a comparable potency to safinamide (**12**, IC_50_ = 51 nM). In addition, compound **32** proved to be a reversible, with adequate metabolic stability in human liver microsome assays and low risk of drug–drug interactions when evaluated against CYP isoenzymes. In vivo studies shown that compound **32** was effective in ameliorating the motor MPTP-induced impairment in a PD model, and raised tyrosine hydroxylase levels in the SN and striatum [62].

More recently, Jismy’s group drew on their experience with bicyclic and tricyclic aromatic compounds to design a new series of pyrimido[1,2-b]indazole analogues (**33**, Figure 6) aimed at inhibiting human MAO-B. A preliminary in vitro evaluation for their inhibitory potential showed a general selectivity for MAO-B, revealing compounds **33a**–**c** as the best selective inhibitors, with IC_50_ values of 0.065, 0.062, and 0.130 µM, respectively. These compounds showed reversible and competitive inhibition modes, as well as low cytotoxicity against SH-SY5Y human neuroblastoma cells, with cell viability above 85% even at concentrations higher than 100 µM. In addition, derivative **33c** showed neuroprotective properties in a PD model in SH-SY5Y cells subjected to 6-OHDA-induced neurotoxicity [63].

Following on from previous studies, Elkamhawy et al. synthesized 26 new indole analogues (Figure 6) designed based on the indoleamide prototype **34** (Figure 6), which was previously identified as a nanomolar MAO-B inhibitor (IC_50_ = 777 nM) [64]. To increase its potency and selectivity, structural modifications were made to the molecular framework of **34**, retaining the indole core, but replacing the heteroaryl subunit with different aliphatic and aromatic substituents to evaluate their pharmacophoric contributions, keeping halogens as substituents in positions 3 and 4 of the arylamide system and replacing the aromatic subunit at C5 with different aliphatic groups. Among all new synthetic compounds, derivative **35** stood out as a selective, competitive, highly potent, and selective MAO-B inhibitor (IC_50_ = 42.1 nM, SI > 2375), with high permeability and bioavailability in CNS (Pe = 54.49 × 10^−6^ cm/s), good oral absorption and excretion profile. In addition, compound **35** showed no cytotoxicity towards PC12 cells, even at doses above 30 µM, and exhibited a neuroprotective effect against 6-OHDA-induced damage [47].

In a continuous effort aimed at structural optimization of the bioactive prototype **34**, Elsherbeny et al. designed a new set of indole-arylamides, leading to identification of the ester derivative **36** (IC_50_ = 0.33 µM), and the diarylamides **37a** (IC_50_ = 0.02 µM), **37b** (IC_50_ = 0.03 µM), and **37c** (IC_50_ = 0.45 µM) as the most promising compounds, with excellent inhibitory potencies and selectivity for MAO-B (SI > 305, 3649, 3278, and 220, respectively), with both **37a** and **37b** acting as reversible and competitive inhibitors. The neurotoxicity of these compounds was assessed using the MTT test on PC12 cells, with only **37b** decreasing cell viability at 30 µM. In addition, these compounds showed a neuroprotective effect for damage induced by 6-OHDA and rotenone in PC12 cells, with compounds **36**, **37b** and **37c** being able to decrease the rotenone- and 6-OHDA-induced production of ROS, and moderate activity in the scavenging of DPPH radicals [52].

#### 3.1.2. Hydrazide and Hydrazone-Based Analogues

**Figure 7:**
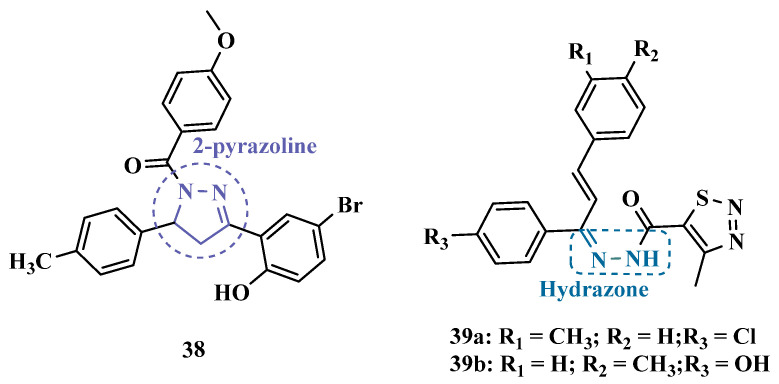
Chemical structures of the most active 2-pyrazoline derivative **38**, and hydrazones **39a** and **39b**.

Hydrazones and their derivatives constitute a wide class of compounds intensively reported in the literature for their diverse biological properties, including anticonvulsant, analgesic, anti-inflammatory, antimicrobial, antidepressant, and MAO inhibitors. Thus, they are quite common as structural fragments in the architecture of ligands and drug candidates, with easy synthetic access from aldehyde and ketone precursors, with good stability towards hydrolysis. Due to their chemical structure based on an azomethine group –NHN = CH–, hydrazones are an analogue class to hydrazines, which can also be considered as their bioisosters. The C = N bond strongly influences their physicochemical properties, since the nitrogen atoms could act as nucleophilic sites, with the –NH position being the most reactive [64]. As an example of the use of the hydrazone subunit as a pyrazole bioisoster, Evranos-Aksöz and co-workers synthesized and evaluated a series of 2-pyrazoline derivatives and its hydrazone analogues as potential MAO inhibitors. Biological data revealed a tendency towards competitive, reversible, and selective inhibition of MAO-A, with emphasis on the most active compounds **39a** and **39b** (Figure 7), with Ki value of 10 nM for both, except for compound **38**, which showed a selective inhibition of MAO-B (Ki = 29.66 µM) [65]. 

**Figure 8:**
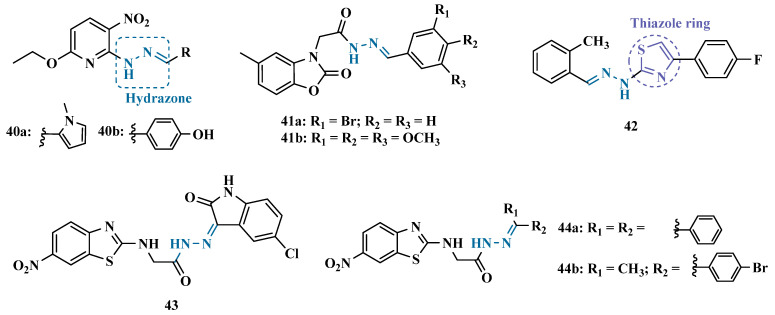
Chemical structures of the most active hydrazone derivatives **40a** and **40b**, the most active and selective acyl-hydrazone derivatives for MAO-B **41a**–**41b**, **43** and **44a**, hydrazone analogue **44b**, and of the most potent and selective MAO-B inhibitor hydrazonyl-thiazole derivative **42**.

MAO inhibitors generally have an amino or imine group in their scaffold. Based on that, Turan-Zitouni and co-workers designed and synthesized a series of fourteen new *N*-pyridyl-hydrazone derivatives (Figure 8) as potential MAO inhibitors. Biological data evidenced compound **40a** with the best MAO-A inhibitory effect (IC50 = 6.12 μM), followed by **40b** as a poor selective and mixed type MAO-A inhibitor, with IC50 values of 6.25 and 9.30 μM for MAO-A and MAO-B, respectively. Both compounds showed non-significant cytotoxicity toward NIH/3T3 cells [66].

In another example, Salgin-Gökşen and colleagues designed a *N*-acyl-aryl-hydrazone scaffold (Figure 8), leading to the discover of new selective, reversible, and competitive MAO-B inhibitors. It was suggested that substitution on the phenyl ring, especially at position 3, significantly increased the inhibitory activity against MAO-B. These findings were corroborated by compounds **41a** and **41b**, which showed the strongest affinities (Ki values of 35.4 and 24.2 nM, respectively), with compound **41b** being more potent than the reference drug selegiline (Ki = 30.35 nM). In addition, the most potent derivative **41b** has also shown a higher selective index of 147.1 for MAO-B than selegiline (SI = 67.9) [67].

Inspired by previous studies with 2-thiazolylhydrazones [68], Distinto and cols. synthesized a set of new 2-thiazolylhydrazone derivatives (Figure 8), to study their MAO inhibitory profile and to understand the pharmacophoric influence of chloro- and fluoro-phenyl substituents of the thiazole ring. Biological data revealed that almost all compounds were inactive against MAO-A at concentrations below 100 µM. Conversely, derivative **42** was identified as a promising selective MAO-B inhibitor (IC_50_ = 0.19 µM), being equipotent to the reference drug rasagiline, and led the authors to suggest that the fluorine substituent at position 4 of the thiazole ring seems to play a central role in the selectivity and inhibitory potency of MAO-B isoform [69].

Also, exploring the thiazole core in the structural architecture of MAO inhibitors, Tripathi & Ayyannan synthesized and evaluated a new series of 2-amino-6-nitrobenzothiazole hydrazones (Figure 8). Biological evaluation, not only focused on enzyme inhibition, but also including kinetic parameters, reversibility, neurotoxicity, and in vivo antidepressant activity, led to the identification of compounds **43** (IC_50_ = 1.8 nM, SI = 766.67) and **44a** (IC_50_ = 4.4 nM, SI = 19,977.27) as the most promising MAO-B inhibitors. On the other hand, compound **44b** was the most active for MAO-A inhibition (IC_50_ = 0.42 µM). Further studies on enzyme kinetics indicated that compounds **43** and **44b** act as reversible and competitive inhibitors against MAO-B and MAO-A, respectively. However, despite their nanomolar range of potency, compounds **43** and **44b** showed an in vivo moderate neurotoxicity, whereas **44a**, exhibited no neurotoxicity, being considered the most promising candidate for neuroprotective use against PD [70].

**Figure 9:**
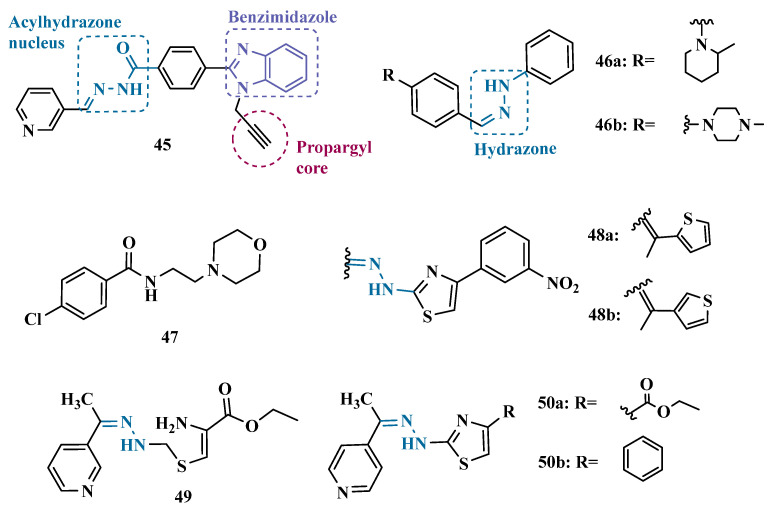
Chemical structures of benzimidazole analogue **45**, 2-phenylhydrazone derivatives **46a** and **46b**, the MAO-A inhibitor moclobemide **47**, the most potent 4-(3-nitrophenyl)thiazol-2-yl hydrazone derivatives **48a** and **48b**, and structural representation of 2-thiazolylhydrazone derivatives **49**, **50a**, and **50b** with selective MAO-B inhibitory properties.

In another strategy, Can and co-workers based their rationale on the MAO inhibitory activity shown by benzimidazole to propose and synthesize a series of fifteen new *N*′-(arylidene)-4-(1-(prop-2-in-1-yl)-1*H*-benzo[d]imidazol-2-yl)benzohydrazides (Figure 9). The authors considered the design of the acylhydrazone side chain inspired by the hydrazone pharmacophore as a mimic of the imine or amino groups commonly present in MAO inhibitors core, in addition to the propargyl pharmacophore for MAO-B present in selective inhibitors such as selegiline (**10**) and rasagiline (**11**). Among all tested compounds, derivative **45** (Figure 9) showed the most pronounced non-competitive and selective activity against MAO-B (IC_50_ = 0.075 µM; SI = 127.813). In addition, this compound showed no significant cytotoxicity in the NIH/3T3 mouse embryonic fibroblast cell line, non-mutagenic effect in the Ames assay, and provided good prediction for drug-like properties including ADME and BBB permeability [71].

From another perspective, Can’s group synthesized fourteen new 2-phenylhydrazone derivatives (Figure 9) and tested them against MAO isoforms. As a result, compounds **46a** and **46b** (Figure 9) exhibited the best MAO-A inhibitory activity with IC_50_ values of 0.342 µM and 0.028 µM, respectively, acting in a reversible and competitive inhibitory mode and being more potent than the reference inhibitor moclobemide (**47**, IC_50_ = 6.06 µM, Figure 9). In addition, both were not significantly cytotoxic against NIH/3T3 cells, and in silico data suggested adequate BBB permeability and hydrolytic stability [64].

Following on from previous studies focusing on a series of thiazole-2-yl hydrazones designed as selective multitarget inhibitors for MAO-B, Secci’s group synthesized another set of new 4-(3-nitrophenyl)thiazole-2-ylhydrazone derivatives and evaluated their ability to inhibit both MAO isoforms. Biological results evidenced **48a** (IC_50 hMAO-B_ = 1.8 nM) and **48b** (IC_50 hMAO-B_ = 2.5 nM, Figure 9) as the most potent nanomolar and selective compounds. Moreover, compound **48a** showed a competitive and partially reversible mode of inhibition, as well as slight antioxidant activity [72].

Chimenti et al. synthesized and evaluated 2-thiazolylhydrazone derivatives, whose structural design considered the conservation of the ethylpyridine subunit linked to the hydrazone function for all analogues, aiming to explore steric hindrance and electronic properties at the C4 position and their effects on MAO inhibition (Figure 9). Compounds **49** (IC_50 MAO-B_ = 0.07 μM; MAO-A = 6.57 μM), **50a** (IC_50 MAO-B_ = 0.13 μM; MAO-A = 6.6 μM), and **50b** (IC_50 MAO-B_ = 0.013 μM; MAO-A = 2.7 μM) (Figure 9) were identified as the most promising MAO-B inhibitors, acting in a selective, reversible and competitive mode of inhibition for **50a**,**b** and a mixed mode for **49** [73].

**Figure 10:**
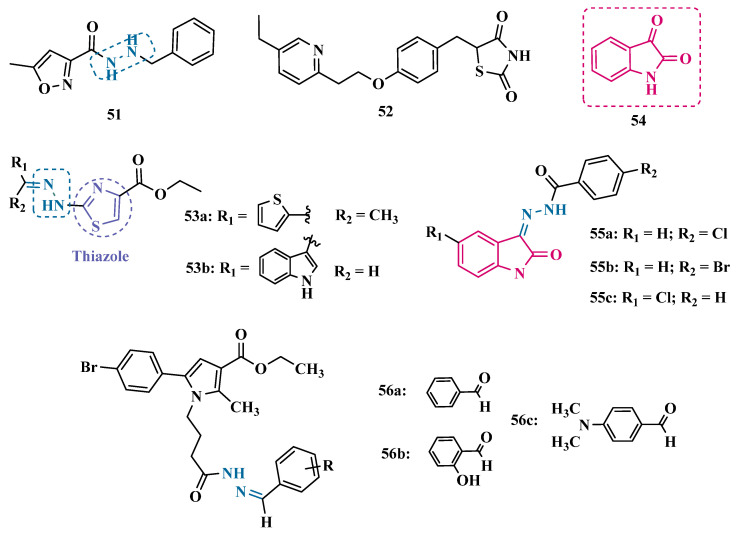
Chemical structures of the isocarboxazide (**51**), pioglitazone (**52**) and the hybrid hydrazothiazole derivatives **53a** and **53b**; isatin (**54**) and the halogenated isatin-derived hydrazones **55a**–**c**, and pyrrole-based hydrazones **56a**–**c**.

The hydrazine subunit is present in the structure of iproniazid (**7**), a drug that has been shown to inhibit MAOs, but which was withdrawn from the market due to its hepatotoxicity and replaced by the lower hepatotoxic isoxazole analogue isocarboxazide (**51**, Figure 10). Computational studies suggest that the hydrazine subunit present in the structure of such compounds contributes to their correct orientation for interaction with the MAO catalytic site. In addition, pioglitazone (**52**, Figure 10), an agonist of the peroxisome proliferator-activated receptor γ (PPARγ), also showed neuroprotective properties due to MAO-B inhibition. Based on these findings, Carradori et al. designed and synthesized a series of hydrazothiazole hybrids, combining the hydrazone fragment of **51** with the thiazole subunit of the PPARγ agonist **52** (Figure 10). Pharmacological screening evidenced compounds **53a** (IC_50_ = 350 nM) and **53b** (IC_50_ = 851.3 nM), with the best selective MAO-B inhibitory profile, inhibiting 40–50% of MAO-A activity at 100 μM [74].

Isatin (**54**, Figure 10) is an endogenous small molecule exhibiting multiple confirmed biological and pharmacological activities, including reversible inhibition of human MAO-A and MAO-B. Based on its structure, Kumar explored the C-3 position with hydrophobic moieties, substituting it with an acyl hydrazone ligand and a halogenated phenyl group as a strategy to develop a new family of effective MAO inhibitors. Among the synthesized compounds, derivatives **55a**, **55b**, and **55c** (Figure 10) stood out for their pronounced activity, exhibiting IC_50_ values of 0.124, 0.082, and 0.104 μM, respectively, acting as reversible and competitive MAO-B inhibitors. These lead derivatives demonstrated high CNS permeability and bioavailability in PAMPA assays, as well as non-cytotoxic, neuroprotective, and anti-inflammatory effects in human SH-SY5Y neuroblastoma cell assays [75].

In previous studies [76,77,78,79], the Georgieva group reported a broad range of pharmacological activities of pyrrole derivatives, highlighting their low toxicity and potential neuroprotective and MAO-inhibitory effects. In a subsequent approach, the group developed a series of pyrrole hydrazone derivatives, with compounds **56a**, **56b**, and **56c** (Figure 10) standing out for their ability to inhibit hMAO-B comparably to selegiline (**10**, Figure 2). Regarding enzyme inhibition, compounds **56a** (IC_50 MAO-B_ = 0.692 μM, SI > 289) and **56b** (IC_50 MAO-B_ = 0.425 μM, SI > 471) exhibited selective MAO-B inhibition, whereas compound **56c** was non-selective (IC_50 MAO-B_ = 0.710 μM; IC_50 MAO-A_ = 0.821 μM; SI = 1.16). Furthermore, the lead derivatives showed low neurotoxicity and significant neuroprotection in oxidative stress models using synaptosomes, mitochondria, and microsomes. The most potent compound, **56b**, was also evaluated in vivo in a rotenone-induced Parkinson’s disease model, demonstrating neuroprotection without histological alterations in neuronal cells [80].

#### 3.1.3. Phthalide, Phthalimide, and Indanone Derivatives

**Figure 11:**
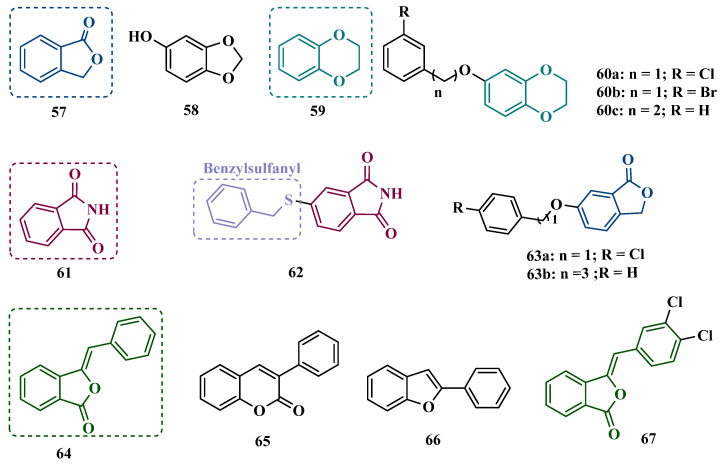
Chemical structures of phthalide **57**, sesamol (**58**), benzodioxane **59**, and the most active hybrid derivatives **60a**–**c**; phthalimide (**61**) and the most active and selective sulfanylphthalimide derivative **62** for MAO-B;phthalide[2-benzofuran-1(3H)-one] derivatives **63a** and **63b**; 3-benzalphthalide (**64**), 3-phenylcoumarin (**65**), 2-phenylbenzofuran (**66**), and the highly selective benzalphthalimide derivative **67**.

Despite side effects such as the “cheese effect” caused by MAO-A inhibition, this isoform continues to be considered a target of interest in the treatment of PD. Notably, it is involved in the control of DpA concentrations, and thus its inhibition could help to improve symptoms of depression, which is often related to PD pathogenesis. However, for therapeutic purposes and improved safety, the inhibition MAO-A must be reversible [81]. Phthalide (**57**, Figure 11) is a structural subunit used in the design of reversible MAO inhibitors which, if properly functionalized, contributes to the inhibitory potency [82,83]. Given this information, Engelbrecht et al. proposed the synthesis of new derivatives of sesamol (**58**, Figure 11) and benzodioxane (**59**, Figure 11), considering that these two compounds have a close structural similarity and could be bioisosters of phthalide (Figure 11). In vitro evaluation on the inhibition of MAO-A and MAO-B revealed increased selectivity for MAO-B and that the benzodioxane derivatives are more potent than sesamol, highlighting compounds **60a** (IC_50_ = 57 nM), **60b** (IC_50_ = 45 nM) and **60c** (IC_50_ = 48 nM) as the most potent and promising reversible and competitive MAO-B inhibitors. Structure-activity relationship (SAR) studies suggested that for benzodioxane derivatives, a phenylethoxy substituent favors MAO-B inhibition, but smaller and more electronegative halogens, such as F and Cl, as substituents in the phenylalkyloxy system seem to disfavor inhibitory selectivity [82].

Although phthalimides (**61**, Figure 11) are not considered strong MAO inhibitors in general, substitutions at the C5 position have led to improved selectivity and potency in the inhibition of MAO-B, such as observed for the halogenated-benzylsulfanyl derivatives. With this in mind, van der Walt and co-workers designed a series of substituted phtalimides aimed at studying the structure-activity effects of diverse substituted-bezylsulfanyl subunits as substituents at the C5 position of the phthalimide core (Figure 11) in the selectivity for MAO-B inhibition. Overall, biological evaluation showed a selective inhibitory activity of MAO-B, and compound **62** (IC_50_ = 4.5 nM, SI = 427) has stood out as the most promising hit, showing a low-nanomolar inhibitory potency, with high selectivity and a quasi-reversible inhibitory manner [83].

In another approach, Strydom et al. synthesized a new series of phthalide[2-benzofuran-1(3H)-one] analogues (**63**, Figure 11) to obtain reversible inhibitors of both MAO isoforms. The rational design was based on the structure of isatin (**54**, Figure 10) and phthalimide (**61**, Figure 11), whose structures have previously been described as suitable for designing promising MAO inhibitors. Bearing in mind that the substitution of a benzyloxy group at the C5 position of both isatin and phthalimide contributes to potentiated activity against MAO-B, substitutions were made at the C6 position of phthalimide, since this is equivalent to the C5 position of isatin. Among all tested compounds against human MAO isoforms, derivatives **63a** (IC_50_ hMAO-A = 0.172 μM, hMAO-B = 2.8 nM) and **63b** (IC_50_ hMAO-A = 0.096 μM, hMAO-B = 6.2 nM) showed a low-nanomolar potency in the inhibition of MAO-B, with 61- and 15-fold higher selectivity, respectively [81].

Benzylphthalides constitute a class of compounds with reported anxiolytic and antiviral activities. In contrast, *Z*-3-benzylphthalides (**64**) possess a trans-stilbenoid core, with a spatial arrangement analogous to that of 3-phenylcoumarins (**65**) or 2-phenylbenzofurans (**66**), two compound families reported as potent MAO inhibitors [84,85,86]. Motivated by the structural similarity of these molecules, Olmo et al. synthesized an initial series of benzylphthalides to identify novel potential MAO inhibitors. The compound exhibiting pronounced and reversible MAO-B inhibition was **67** (IC_50_ = 0.0006 μM), comparable to the reference inhibitor lazabemide (**26**, IC_50_ = 0.091 μM). Moreover, this analog provided significant protection against apoptosis and 6-OHDA–induced mitochondrial toxicity in SH-SY5Y cells [87].

**Figure 12:**
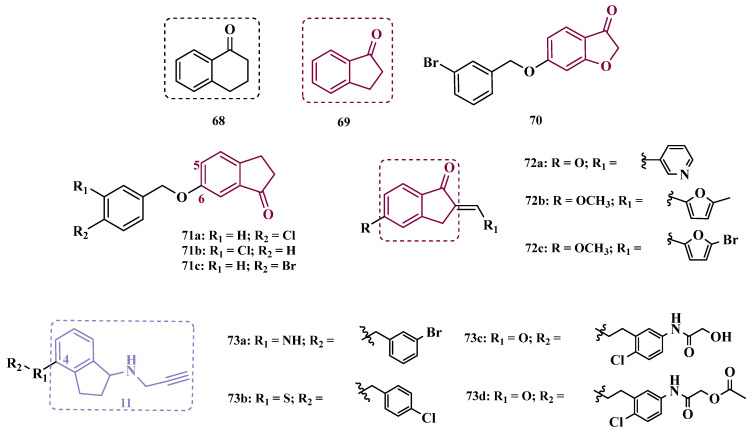
Chemical structures of α-tetralone (**68**), 1-indanone (**69**) and the 3-coumaranone derivative **70**; derivatives **71a**–**71c**, 2-heteroarylidene-1-indanone derivatives **72a**–**72c**, and rasagiline and its derivatives **73a**–**73d**.

Derivatives of α-tetralone (**68**) and 1-indanone (**69**, Figure 12) have also been described as potent MAO inhibitors. Thus, Dyk et al. synthesized a series of 3-cumaranone derivatives **70** (Figure 12), designed based on the structural feature of **68** and **69**, leading to the identification of selective inhibitors of MAO-B, especially for compound **70** (IC_50_ = 4 nM), showing a 23- and 12-fold higher potency than the reference drugs lazabemide (**26**, IC_50_ = 0.091 µM) and safinamide (**12**, IC_50_ = 0.048 µM), respectively, acting by a reversible and competitive mechanism [88].

Given the structural similarity between α-tetralone (**68**) and 1-indanone (**69**), and the previous identification of the nanomolar MAO-B inhibitor **63**, Mostert & co-workers designed a new set of 1-indanone (**71**, Figure 12), and indane derivatives. Rationalization of the experimental data suggested that C6-substituted-1-indanone derivatives were more able to inhibit MAO-A and MAO-B, leading to the identification of compounds **71a** (IC_50 MAO-A_ = 0.032 µM; MAO-B = 2 nM), **71b** (IC_50 MAO-A_ = 0.084 µM; MAO-B = 2 nM), and **71c** (IC_50 MAO-A_ = 0.039 µM; MAO-B = 3 nM) as the most potent and reversible inhibitors, with high selectivity for MAO-B isoform. These Compounds were then selected for further studies as promising drug candidates for the development against PD and depression. Furthermore, 1-indanone derivatives substituted at the C5 and C6 positions showed a significant increase in the inhibitory potency of both isoforms, especially when halogens (e.g., Cl, Br) were attached to the phenyl ring of the benzyloxy subunit [89].

Nel and co-workers explored the 2-heteroarylidene-1-indanone architecture (Figure 12) in the search for new MAO inhibitors. Pharmacological data led to the selection of **72a** (IC_50_ = 0.061 μM), **72b** (IC_50_ = 0.026 μM), and **72c** (IC_50_ = 4.4 nM) as significantly potent MAO-A inhibitors, outperforming the reference compounds toloxatone (**25**, IC_50_ = 3.92 μM), an MAO-A inhibitor, and the reference MAO-B inhibitors lazabemide (**26**, IC_50_ = 0.091 μM) and safinamide (**12**, IC_50_ = 0.048 μM) [90].

In previous studies, Li and co-workers used the fragment-based drug discovery strategy to evaluate increasing hydrophobic fragments located at the 4-position of rasagiline (**11**). Following the same strategy, they synthesized a series of 2,3-dihydro-1*H*-inden-1-amine derivatives (Figure 12), and identified compounds **73a** (IC_50_ = 0.11 µM), **73b** (IC_50_ = 0.18 µM), **73c** (IC_50_ = 0.27 µM), and **73d** (IC_50_ = 0.48 µM), as moderate MAO-B inhibitors in comparison to selegiline (**10**, IC_50_ = 0.06 µM), with similar selectivity of the reference drug [91].

#### 3.1.4. Chalcones

**Figure 13:**
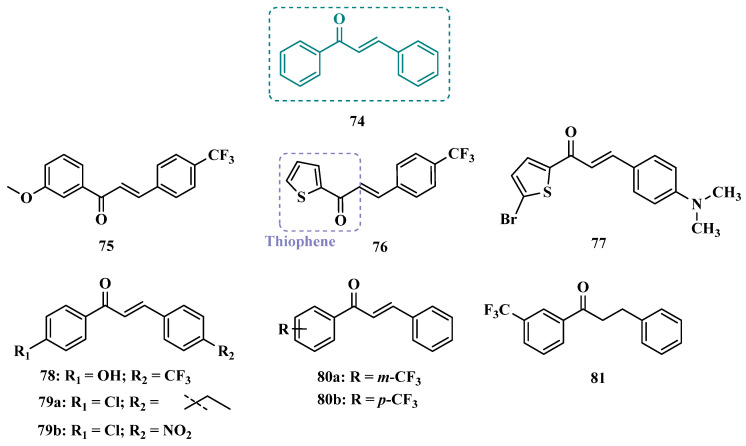
Chemical structures of the chalcone **74** and the MAO-B inhibitors **75**, **76**, **80a**,**b** and **81**; brominated thienyl-chalcone **77**, bromo-thienyl-chalcone **78**, and derivatives **79a** and **79b**.

Chalcones (**74**, Figure 13) are heterocyclic compounds that can exist as both *cis* and *trans*-isomers [92]. Chalcone derivatives, especially with the most stable *trans* configuration, are abundant in nature and have been reported for their wide spectrum of pharmacological properties, including analgesic, antipyretic, and anti-inflammatory [93]. More recently, chalcones have also been described as selective MAO-B inhibitors, justifying the great interest in exploring their singular structural architecture in the design of new selective and reversible MAO-B inhibitors. In addition, this class has the ability to bind to the benzodiazepine site of γ-aminobutyric acid (GABA) and result in a sedative effect in rats [94,95].

Inspired by the structure of fluorine- and trifluoromethyl-substituted chalcones, which have been described for their antidepressant, antipsychotic, and anxiolytic properties, Mathew and co-workers conceived and synthesized a new series of fluorinated chalcones. Biological evaluation revealed the methoxy-trifluoromethyl derivative **75** as the most promising reversible and competitive MAO-B inhibitor (Ki = 0.22 µM, Figure 13), showing higher affinity than the reference drug selegiline (**10**, Ki = 0.33 µM) [96]. In another approach, the same group explored the bioisosteric replace for the thiophene subunit on **75**, which is present in the structure of MAO inhibitors and antidepressants, leading to a novel series of thiophene-fluorinated chalcones (Figure 13). Biological screening evidenced an apparent selectivity for MAO-B, highlighting compound **76** as the most potent, competitive MAO-B inhibitor (Ki MAO-B = 0.90 µM, MAO-A = 4.88 µM, SI = 5.42), acting as a reversible inhibitor for both isoforms [97].

Taking into account literature data, showing that the presence of lipophilic fragments in addition to electron withdrawer substituents in the *para* position of the B ring of chalcones lead to increased inhibitory potency against MAO-B, Mathew’s group designed another series of brominated thienyl-chalcone derivatives (Figure 13), aiming at a SAR study regarding various substituents located at the *para* position of the phenyl B ring. Overall, pharmacological data demonstrated compound **77** (Ki = 0.11 μM, SI = 13.18) as the most promising ligand, showing the greatest affinity and selectivity for MAO-B, without cytotoxicity in the human liver cancer cell line HepG2. Moreover, compound **77** showed a reversible inhibition of hMAO-B, with a competitive inhibition mode for both isoforms and good BBB permeability in the PAMPA assay [92]. In another work, the same group has observed that methoxylated chalcone derivatives containing a fluorine as substituent showed high affinities for MAO-B (Figure 13). Furthermore, it was also seen that the biological activity is influenced both by the nature and position of the fluorine groups on the B ring, as well as the nature and position of other substituents on the A ring. Thus, the authors synthesized a set of chalcone derivatives aimed at evaluating the pharmacophoric contribution of diverse substituents on the chalcone core in the MAO inhibition, especially focusing on the effects of a *para*-hydroxy substituent in ring A, which is a better H-bond donor and acceptor when compared to the *para*-methoxyphenyl. The most active compound was **78** (Ki = 0.30 µM and SI = 26.36), showing a reversible, high affinity and selectivity for MAO-B [98].

Based on the same premise that heterocyclic substituents on chalcones play a positive role in MAO inhibition, as well as the presence of lipophilic groups in the *para* position of the B ring favored inhibition of the MAO-B isoform, Mathew’s group prepared and evaluated another set of chalcone derivatives containing lipophilic substituents in the *para* position of the A ring, and a variety of electron donor and withdrawer substituents in the *para* position of the phenyl B ring. Compound **79a** (Figure 13, Ki = 0.11 µM, SI = 16) was identified as the most potent inhibitor against MAO-B, whose activity was more pronounced compared to the reference drug selegiline (**10**, Ki = 0.35 µM, SI = 15.8), while compound **79b** (Ki = 0.18 µM, SI = 0.13) stood out for its pronounced activity against MAO-A. Both proved to be competitive and reversible, able to cross the BBB in vitro and showed no cytotoxicity in in vitro studies with liver cells [99].

Considering the broad range of biological activities exhibited by chalcones (**74**, Figure 13), including anticancer, anti-inflammatory, and antioxidant effects, Lacovino et al. explored a small library of mono-substituted chalcone derivatives with the aim of identifying novel MAO inhibitors. Among the synthesized compounds, derivatives **80a** and **80b** (Figure 13), containing the same CF_3_ substituent at the meta and para positions, respectively, stood out for their high selectivity toward the MAO-B isoform, displaying Ki values of 5.0 and 14.6 nM, respectively. Notably, these derivatives exhibited a safe profile with low in vivo toxicity, alongside antiproliferative activity in tumor cell lines (A2780, HT-29, and MSTO-211H). Regarding pharmacokinetics, further studies demonstrated that compound **80a** acts as a competitive and reversible inhibitor, reinforcing its potential as a selective MAO-B inhibitor [100].

Subsequently, the same group designed a new series of chalcone analogs based on the **80a** structure, aiming to identify optimized MAO-B inhibitors. Structural optimizations targeted three specific regions: the *m*-CF_3_-phenyl ring, through the introduction of electronegative substituents; the α,β-unsaturated carbonyl intermediate; and the benzene ring. These derivatives were strategically designed to enhance binding affinity to the MAO-B active site, with compound **81** (Figure 13) identified as the most potent inhibitor (Ki = 0.875 μM). Derivative **81** was notable for its selectivity and minimal effects on the viability of both cancerous and normal cell lines, while also demonstrating potential neuroprotective effects in SH-SY5Y cells exposed to 6-OHDA, a Parkinson’s disease model [101].

**Figure 14:**
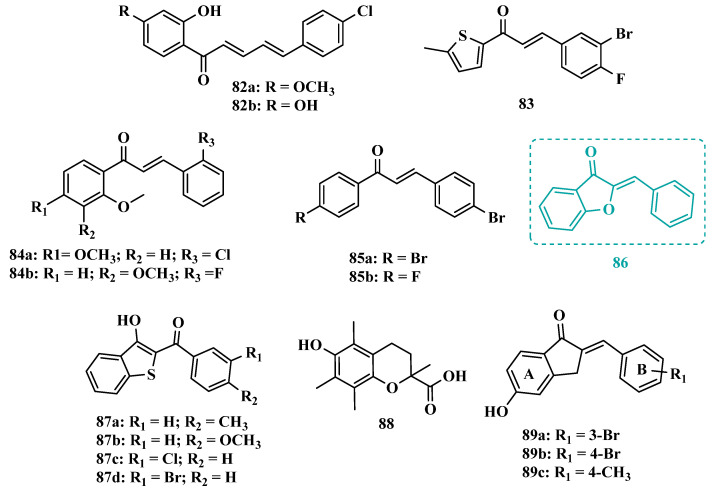
Chemical structures of the active chalcone derivatives **82a**,**b**, **83**, **84a**,**b**, and **85**; aurone (**86**) scaffold, benzo[b]thiophen-3-ol derivatives **87a**–**d**, trolox (**88**), and structure of the 2-Benzylidene-1-indanone analogues **89a**–**89c**.

Still exploring the structure of chalcones as molecular prototypes, Desideri and co-workers synthesized two series of (2*E*,4*E*)-1-(2-hydroxyphenyl)-5-phenylpenta-2,4-dien-1-one and (2*Z*,4*E*)-3-hydroxy-1-(2-hydroxyphenyl)-5-phenylpenta-2,4-dien-1-one analogues. Biological data revealed that among all tested compounds those with 2*E*,4*E* configuration (Figure 14) were significantly active, with **82a** (IC_50_ = 4.51 nM) and **82b** (IC_50_ = 11.35 nM) identified as the most potent and selective MAO-B inhibitors, acting in a reversible mode, with derivative **82b** also showing significant inhibition of MAO-A (IC_50_ = 15.37 μM) [102].

In another approach, the Minders’ group explored a series of heterocyclic chalcone analogues as potential reversible MAO inhibitors. The pharmacological evaluation highlighted compound **83** (Figure 14) as the most active and selective MAO-B inhibitor (IC_50_ = 0.067 µM, SI = 240), with a similar potency as for the reference drug lazabemide (**26**, IC_50_ = 0.091 µM). However, kinetic studies evidenced that **83** was not a reversible inhibitor, which could be explained by its strong binding to the active site, probably involving the thiophene subunit. Furthermore, this compound seems to act as a competitive inhibitor and did not show significant toxicity in HeLa cells at the concentrations of 1 µM to 10 µM [103].

More recently, inspired by literature data suggesting that halogen- and methoxy-substituted chalcones can exhibit good MAO-B inhibitory activity, Rehuman & colleagues synthesized new dimethoxy-halogenated chalcone derivatives, which were subjected to an in vitro biological screening. As a result, compounds **84a** (IC_50_ = 0.067 µM; SI = 93.88) and **84b** (IC_50_ = 0.118 µM; SI > 338.98, Figure 14) were identified as the most potent and selective MAO-B inhibitors, acting in reversible and competitive mode. In addition, compound **84a** did not show significant cytotoxicity on Vero cells at concentrations below 100 µg/mL [104].

In a similar proposal, Abdelgawad and co-workers synthesized two other series of F-and Br-substituted chalcone derivatives as potential MAO inhibitors (Figure 14). Biological results showed that in both series, there was a predominance of MAO-B selectivity, and in the brominated series, compound **85a** showed the best selective inhibition (IC_50 MAO-B_ = 6.2 nM; SI = 938.7), whereas in the fluorine-containing series, compound **85b** (IC_50 MAO-B_ = 0.011 µM; SI = 475.5, Figure 14) stood out. Interestingly, in both cases, the excelled compounds were shown to act by reversible and competitive modes, with significantly higher inhibitory potencies than the reference MAO-B inhibitors lazabemide (IC_50_ = 0.11 µM) and pargiline (IC_50_ = 0.14 µM) [105].

Inspired by the structure of indanone (**69**, Figure 12) and aurone (**86**, Figure 14), whose derivatives have been reported as selective MAO-B inhibitors, Guglielmi and co-workers synthesized a new series of benzo[b]thiophen-3-ol derivatives as potential MAO inhibitors (Figure 14), keeping their similar structural feature such as the bicyclic indanone system connected by a bridge to an aromatic/heteroaromatic ring. The isosteric repositioning of the oxygen atom of the aurones by a sulfur atom, as well as the presence of a 1,3-diketone system, resulted in its corresponding chalcone via keto-enolic tautomerism, probably preserving the metal chelation ability. In general, all new compounds demonstrated selectivity for MAO-B inhibition, and compounds **87a** (IC_50__MAO-A_ = 2.71 μM; MAO-B = 0.47 μM), **87b** (IC_50__MAO-A_ = 4.18 μM; MAO-B = 0.28 μM), **87c** (IC_50__MAO-A_ = 51.0 μM; MAO-B = 0.55 μM), and **87d** (IC_50__MAO-A_ = 63.2 μM; MAO-B = 0.35 μM) showed the best in vitro results. Further studies towards the 3,4-dihydroxyphenylacetic acid/dopamine (DOPAC/DpA) ratio and lactate dehydrogenase (LDH) activity highlighted compound **87d** as the best inhibitor in both cases. Regarding antioxidant and metal chelation ability, compound **87a** showed comparable activity to the reference compound trolox (**88**, Figure 14) [106].

Similarly, Nel and co-workers synthesized 2-benzylidene-1-indanone derivatives (Figure 14), which can be considered cyclic chalcone analogues, to explore the ability of these compounds to inhibit MAO. The rational structural design included changes in the substituents of ring A, including hydroxy and methoxy groups at positions 5 and 6. In turn, ring B was substituted at positions 3 and 4 with halides, alkyl, amine, and hydroxy groups. Pharmacological screening revealed some selective MAO-B inhibitors, but compound **89a** stood out for its reversible inhibition of MAO-A (IC_50_ = 0.131 μM). Differently, the equipotent compounds **89b** (IC_50_ = 5.3 nM) and **89c** (IC_50_ = 5.2 nM) showed significant selective nanomolar inhibitory activity against MAO-B isoform, with a comparable inhibitory profile as for the reference inhibitors lazabemide (**26**, IC_50_ = 0.091 μM), an MAO-B inhibitor, and toloxatone (**25**, IC_50_ = 3.92 μM), an MAO-A inhibitor [107].

#### 3.1.5. Propargylamine and Phtalonitrile Derivatives

**Figure 15:**
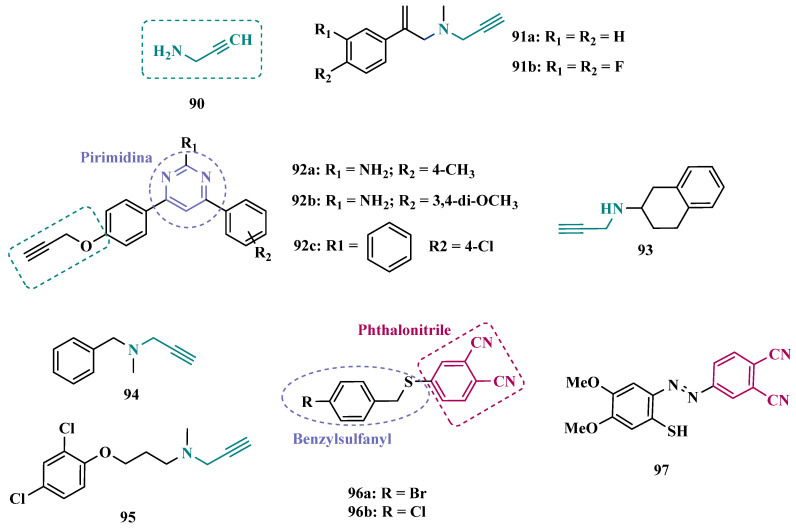
Chemical structures of the propargylamine subunit **90**, arylalkenylpropargylamine derivatives **91a**,**b**, pyrimidine analogues **92a**–**c**, *N*-propargylamine-2-aminotetralin derivative **93**, pargyline **94**, clorgyline **95**, sulfaniphthalonitrile derivatives **96a**,**b**, and the phthalonitrile derivative **97**.

The propargylamine subunit (**90**, Figure 15) is an important pharmacophore for MAO inhibition and is present in the structure of drugs such as selegiline (**10**) and rasagiline (**11**). Experimental data support that this subunit in rasagiline is responsible for neuroprotection due to its role in neutralizing several steps in the apoptotic cascade and, consequently, preventing cell death [108,109]. Based on these findings, Huleatt and co-workers proposed new aryl-alkenylpropargylamine analogues (Figure 15) as potential dual neuroprotective and MAO inhibitors. Biological data revealed **91a** (IC_50 MAO-B_ = 60 nM, SI = 58) and **91b** (IC_50 MAO-B_ = 2.3 nM, SI = 1347) as the most promising compounds, showing significant neuroprotective activity on PC12 cells, selective MAO-B inhibition in a nanomolar range, and low toxicity in the TAMH cell line [109].

Another structural framework worth mentioning is pyrimidine, a subunit present in several bioactive compounds, which could be the basis for other heterocyclic compounds with a good pharmacological profile, including neuroprotection. With this in mind, Kumar’s group synthesized 2,4,6-trisubstituted pyrimidine derivatives containing an *O*-propargyl subunit (Figure 15). Biological data suggested a reversible and selective inhibition of MAO-B for almost all tested compounds, particularly for **92a** (IC_50_ = 0.38 µM) as the most potent MAO-B inhibitor, and for the equipotent analogues **92b** (IC_50_ = 0.51 µM) and **92c** (IC_50_ = 0.48 µM) that showed the highest selectivity index (SI~100) for the same isoform. Moreover, **92b** led to a decreasing intracellular ROS formation, and none of these three bioactive ligands showed significant cytotoxicity against SH-SY5Y neuroblastoma cells at 25 µM [110].

More recently, still inspired by the propargylamine subunit as a pharmacophore for MAO inhibition, Meiring and co-workers synthesized the *N*-propargylamine-2-aminotetralin derivative **93** (Figure 15) as a racemate. In vitro biological evaluation showed selective, reversible and competitive inhibition of MAO-A (IC_50 MAO-A_ = 0.721 µM, MAO-B = 14.6 µM), with higher potency than the propargylamine reference drug pargyline (**94**, IC_50 MAO-A_ = 15.6 µM), but less potent than clorgyline (**95**, Figure 15, IC_50 MAO-A_ = 2.6 nM), selegiline (**10**, IC_50 MAO-B_ = 0.095 µM), and toloxatone (**25**, MAO-B: IC_50_ = 3.92 µM) [111].

In the vast chemical space of scaffolds that have been studied as potential MAO inhibitors, nitrile compounds such as phthalonitriles and benzonitriles are included, and several hits have been reported for their selective ability to inhibit MAO-B, and whose affinity can be explained by their highly polar nature. On the other hand, the benzyloxy substituent is present in MAO-B inhibitors such as safinamide and seems to contribute to the binding affinity with this isoform. Thus, van der Walt and co-workers synthesized new phthalonitrile and benzonitrile derivatives substituted with a benzylsulfanyl subunit (Figure 15) aiming at the development of novel potent and selective MAO-B inhibitors. Biological data revealed that sulfanylphthalonitriles exhibited higher inhibitory potency than sulfanylbenzonitriles, especially compound **96a** (IC_50 MAO-B_ = 0.025 µM and SI = 8,720), which stood out for its high potency and reversible mode of MAO-B inhibition, showing to be similar to the reference drug deprenyl (IC_50 MAO-B_ = 0.079 µM). Although, all compounds showed generally low inhibition of MAO-A, a worth mentioning compound is **96b** (IC_50 MAO-A_ = 0.623 µM), which showed pronounced inhibition of MAO-A, despite being less potent than clorgyline (**95**, IC_50_ = 2.6 nM) [112].

Also inspired by the structure of phthalonitriles, Ali and co-workers synthesized a new series of benzylimines (Figure 15) as potential MAO inhibitors. In vitro data suggested a tendency for selective MAO-B inhibition, especially for compound **97** (IC_50 MAO-A_ = 55.62 µM, MAO-B = 0.74 µM) which exhibited a selective index of 75.16, good in vitro BBB permeability, and adequate drug-like properties, but a 37-fold lower potency than selegiline (**10**, IC_50 MAO-B_ = 0.02 µM) [113].

#### 3.1.6. Alkaloids

**Figure 16:**
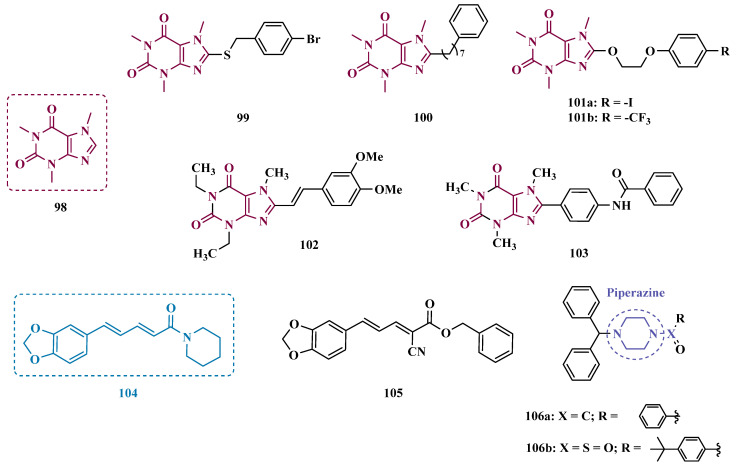
Chemical structures of caffeine (**98**) and its most active derivatives **99** and **100**; 8-(2-phenoxyethoxy)-caffeine analogues **101a**,**b**, KW-6002 (**102**) and its analogue **103**; piperine (**104**) and its MAO-B selective derivative **105** and phenyl- and benzyl-piperazine derivatives **106a**,**b**.

Caffeine (**98**, Figure 16) is a xanthine derivative whose ability to inhibit MAO has been described in the literature, especially when substituted at C8 position, which leads to an increase in its inhibitory potency against MAO-B [114,115]. Thus, taking its structure as a prototype core, Booysen and co-workers designed new reversible MAO inhibitors as new amino caffeine analogues (Figure 16). These compounds were evaluated for their ability to inhibit recombinant human MAO-A and MAO-B, and compound **99** (IC_50 MAO-A_ = 2.62 µM, MAO-B = 0.167 µM) stood out as the most active ligand among a set of sulfanyl-caffeine derivatives, with a *p*-Br-benzyl-thioether as side chain. These findings were corroborated by further in silico studies, suggesting that longer side chain attached to C8, as well as halogen substituents on the phenyl system in the same fragment, contribute to increasing the inhibitory potential against MAO-B [116].

Similarly, Petzer and colleagues synthesized other xanthine analogues substituted at the C8 position with phenylalkyl moieties (Figure 16), leading to the discover of compound **100** as the most potent in the series, operating as a selective, reversible and competitive inhibitor of MAO-B (IC_50_ = 0.086 µM), being equipotent to lazabemide (IC_50_ = 0.091 µM). Notably, this analogue also showed significant activity against MAO-A (IC_50_ = 3.01 µM) and, despite being 35-fold more potent for MAO-B, it can be compared to the reference MAO-A inhibitor toloxatone (IC_50_ = 3.92 µM) [115].

Considering that 8-benzyloxycaffeine analogues have shown to be potent and reversible MAO-A inhibitors, Strydom’s group synthesized a series of 8-(2-phenoxyethoxy)-caffeine, exploring the contributions of diverse *para*-substituents of the phenoxy system ring against MAO inhibition. In vitro results highlighted **101a** and **101b** (Figure 16) as the most potent compounds with IC_50_ values of 0.924 µM and 0.061 µM for MAO-A and MAO-B, respectively, both acting as reversible inhibitors [114].

Beyond caffeine, other xanthine-based compounds with MAO inhibitory properties, such as the dimethoxy-styrene KW-6002 (**102**, Figure 16), are representants of this chemical space and reinforce the importance of aza-heterocyclic compounds for MAO-B inhibition. Thus, Song and co-workers synthesized and evaluated a series of new xanthine derivatives with an 8-(benzamido)-phenyl substituent attached to the xanthine system. Among all tested compounds, derivative 103 (Ki = 0.26 µM) showed the best in vitro MAO-B inhibition, with significantly higher affinity than the parent prototype 102 (Ki = 11 µM), which was used as a reference [117].

Another alkaloid of interest is piperine (**104**, Figure 16), one of the most abundant secondary metabolites in chili pepper, which have been reported by its wide range of biological properties, including antioxidant, anti-inflammatory, anti-apoptotic and competitive, reversible and non-selective inhibition of MAO. Therefore, based on previous studies in which bioisosters of piperine have shown MAO inhibition and confirmed the relevance of the piperidine amide subunit for enzymatic interaction [118], Chavarria and co-workers synthesized new piperine derivatives to evaluate their neuroprotective effects and MAO inhibition. Biological data results evidenced compound **105** (IC_50 MAO-B_ = 0.0474 µM, Figure 16) as the most promising derivative and as a selective and competitive MAO-B inhibitor, with no significant cytotoxicity against SH-SY5Y and CACO-2 cells and good BBB permeability [119].

Piperazine is a privileged structure with a good pharmacodynamic and pharmacokinetic profile, which is present in several FDA-approved drugs such as antidepressants, anti-ketonergics, and tranquilizers, and is a subunit present in several psychoactive compounds with MAO inhibitory activity [120]. Inspired in such a structure, Kumar and co-workers synthesized new phenylpiperazine and benzhydrylpiperazine compounds designed as potential MAO inhibitors. Biological results evidenced a general selectivity for MAO-B, highlighting compound **106a** (Figure 16, IC_50_ = 80 nM) as a nanomolar and reversible inhibitor. On the other hand, the *tert*-butylphenyl analogue **106b** (IC_50 MAO-A_ = 120 nM) showed a nanomolar potency, but its selectivity was significantly higher for the MAO-A isoform. Moreover, both compounds showed a safe cytotoxicity profile towards SH-SY5Y and IMR-32 cells, with good BBB permeability, and **106b** significantly reduced intracellular ROS levels [121].

#### 3.1.7. Benzopyrone Derivatives

**Figure 17:**
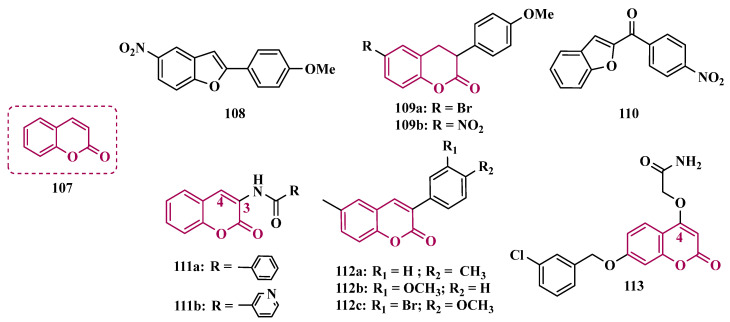
Chemical structures of coumarin (**107**), 2-arylbenzofuran derivatives **108**, **109a**, **109b**, 2-aroylbenzofuran **110**, 3-benzamidocoumarin **111a**, 3-heteroarylamidocoumarin **111b**, 3-arylcoumarins **112a**–**c**, and oxymethylene-amide derivative **113**.

Coumarin (**107**, Figure 17) is a privileged structure in medicinal chemistry, and its multitarget profile has been described for wide pharmacological properties, including anti-inflammatory, antidepressant, anticonvulsant, antibacterial, and neuroprotection for NDs, [122,123]. Thus, the coumarin core has attracted attention as a promising structural framework for the development of antioxidant compounds and enzyme inhibitors, such as MAO [124,125]. Ferino and co-workers synthesized a series of 2-arylbenzofurans and 3-arylcoumarins designed as potential MAO-B inhibitors. Biological studies led to identification of **108** (Figure 17) as the most active benzofuran derivative (IC_50_ = 0.14 µM), showing good selectivity (SI > 714), and a reversible inhibition of MAO-B. Among the coumarins, compounds **109a** (IC_50_ = 6 nM; SI > 16,667) and **109b** (IC_50_ = 3 nM; SI = 390, Figure 17) exhibited the best pharmacological profile against MAO-B, with a highlighted low-nanomolar potency range, and a reversible inhibition mode [124].

Guglielmi and colleagues, aiming to develop potent and selective MAO-B inhibitors, designed a new series based on two distinct structural motifs: 2-aroylbenzofuran-3-ol and 2-aroylbenzofuran. In general, the 2-aroylbenzofuran-3-ol derivatives proved incapable of inhibiting MAO isoforms, whereas the 2-aroylbenzofurans, in contrast, acted as potent inhibitors, with compound **110** (IC_50__MAO-B_ = 0.0082 μM, SI > 1221) emerging as the lead of the series. Regarding cytotoxicity against two different cell lines, SH-SY5Y and hGF, derivative **110** displayed a safe profile, not significantly interfering with cell viability, in addition to exhibiting neuroprotective activity in SH-SY5Y cells treated with 6-OHDA. Finally, in the in silico ADMET profile prediction, the derivative was shown to be capable of crossing the BBB [126].

In the search for multifunctional compounds capable of slowing down the progression of NDs, Matos and co-workers also explored the coumarin core in the design and synthesis of a set of 3-amidocoumarin derivatives. The rational design was based on literature data suggesting that adequate substitutions at the C3 and C4 positions could lead to improved multiple inhibition of cholinesterases, β-secretase, and MAOs. Thus, the authors proposed the substitution of the C3 position with an amide group and the C4 position with either a hydroxyl group or a hydrogen. As a result, in series of 3-benzamidocoumarins, compound **111a** (IC_50_ = 0.76 μM) stood out in terms of selective MAO-B inhibition, whereas among the 3-heteroarylamido coumarin series, compound **111b** (IC_50 MAO-B_ = 21.1 μM) was not as pronounced as compound **111a**, but showed significant neuroprotective and non-cytotoxic capacity in rat cortical neurons better selectivity, besides good ability to cross BBB, adequate ADME and druggability parameters [123]. In another approach, the same research group designed a series of 6-methyl-3-arylcoumarins, which were evaluated for their ability to inhibit both MAO isoforms. Compounds **112a** (IC_50_ = 0.31 nM, SI > 3300), **112b** (IC_50_ = 0.80 nM), and **112c** (IC_50_ = 0.74 nM, Figure 17) were identified as the most active coumarin derivatives, selectively inhibiting MAO-B with nanomolar potency. Notably, compound **112a** exhibited 64-fold greater selectivity than the reference drug selegiline [127]. Bearing in mind that the C4 position of coumarins plays a crucial role in the binding mode of these inhibitors to the active site of MAO-B, Pisani’s group synthesized a series of new 4-substituted coumarin derivatives. In vitro data demonstrated that smaller polar and hydrophobic substituents at the C4 position resulted in an improved pharmacological profile, as observed for the most active and selective MAO-B inhibitor, the oxymethylene-amide derivative **113** (IC_50_ = 3.1 nM, SI = 7244, Figure 17) [128].

**Figure 18:**
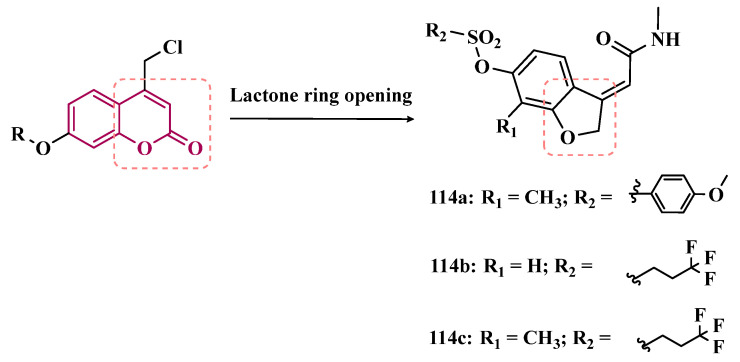
Structural representation of the lactone ring opening of 7-sybstituted coumarins, leading to the discovery of the new (*E*)-2-(benzofuran-3(*2H*)-ylidene)-*N*-methylacetamide derivatives **114a**–**c** as selective and nanomolar MAO-A inhibitors.

As a result of an unexpected lactone-opening reaction of 7-substituted coumarins and primary amines, Pisani and cols. discovered new MAO-inhibiting (*E*)-2-(benzofuran-3(*2H*)-ylidene)-*N*-methylacetamide derivatives. In vitro pharmacological studies on their inhibitory activity against MAO isoforms demonstrated a general selective effect on MAO-A. Considering that a methyl substituent in the structure of the 4,7-disubstituted coumarin considerably increases the affinity for the MAO isoforms, and that the C7 position of the benzofuran core is topologically equivalent to the C8 position of the coumarin system, a methyl group was inserted at C7 in the structure of the new compounds. Biological data highlighted compound **114a** (Figure 18, IC_50_ = 7.0 nM) as the most active MAO-A inhibitor, showing a 1430-fold higher potency than the reference drug moclobemide (**47**, IC_50_ = 10 μM), followed by the analogues **144b** (IC_50_ = 9.1 nM) and **114c** (IC_50_ = 11 nM), which showed a comparable nanomolar potency, besides potential safer pharmacological and toxicological profile. Molecular modeling studies suggested that structural geometry plays a crucial role in molecular recognition, and that the *E* configuration maintains the molecule in a bent arrangement, which is important for its binding to the MAO binding site, especially MAO-A, which has a wider and less flat binding site compared to MAO-B [129].

Additional literature data suggest that substitutions at the C3 position of the coumarin core with electron-donating or electron-withdrawing groups are important for modulating inhibitory activity and selectivity toward MAO-B. Based on this, Meletli et al. synthesized and evaluated a new series of benzocoumarin derivatives for their inhibitory activity. The most active and selective compound for MAO-B was **115** (IC_50 MAO-B_ = 0.013 μM, SI > 7693.31), acting as a reversible mixed-type inhibitor. Furthermore, the lead compound **115** exhibited favorable ADMET properties, including blood–brain barrier permeability, high gastrointestinal absorption, and no violations of Lipinski’s rules [130].

**Figure 19:**
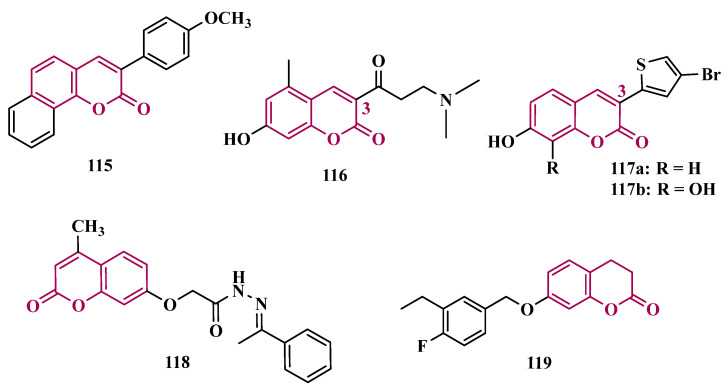
Chemical structures of benzocoumarin derivative **115**, 3-carboxydimethylethylamine-7-hydroxyl coumarins derivative **116**, thiophenylcoumarin derivatives **117a** and **117b**, and the coumarin derivatives **118** and **119**.

Following the same strategy, Tao’s group synthesized new Mannich base derivatives of 3-acetyl-7-hydroxyl coumarins, aimed at identifying novel MAO inhibitors with improved hydrophilicity and biological properties. Among all tested derivatives, **116** (Figure 19, IC_50__MAO-B_ = 3.66 µM; SI > 100) was identified as the most potent MAO inhibitor, with additional neuroprotective and anti-inflammatory properties in SH-SY5Y and BV2 cells, respectively. Moreover, compound **116** was submitted to in vivo studies with MPTP-induced PD models, showing a significant improvement in motor symptoms and an increase in tyrosine hydroxylase expression [131].

Following a similar proposal, Rodríguez-Enríquez and cols. synthesized and evaluated new 3-thiophenyl coumarin compounds as MAO-B inhibitors. In their previous studies, it was observed that substituents at the C8 position of the coumarin structure play a role in the modulation of MAO-B inhibition [132,133], especially for hydroxyl groups that led to additional increased antioxidant and neuroprotective properties of the compounds. Thus, exploring a bioisosterism-based structural design, the authors exchanged the aryl ring for a heteroaryl thiophene ring at the C3 position of the coumarin core. Compound **117a** (Figure 19, IC_50__MAO-B_ = 0.14 µM, SI = 65.43) showed the best selective and reversible inhibitory activity of MAO-B. Further in vivo assays evidenced its ability to improve motor activity more effectively than selegiline (**10**). On the other hand, despite being 155-fold less potent in the inhibition of MAO-B, compound **117b** (IC_50_ _MAO-B_ = 21.75 µM) also showed neuroprotective, significant DPPH radical scavenging ability and inhibition of ROS formation, adequate drug-like physicochemical properties, without neurotoxicity [134].

In another approach to explore the coumarin architecture to predict intermolecular interactions with MAO binding sites and select potential selective inhibitors, Siju and co-workers designed a series of five coumarin derivatives for subsequent molecular modeling studies. As a result of its higher binding affinity, derivative **118** (Figure 19) was then selected for synthesis and further in vitro studies, showing non-selective equipotent inhibition of human MAO-A and MAO-B with an IC_50_ values of 3.70 and 3.90 μM, respectively. In addition, this compound showed significant radical scavenging activity in the DPPH assay, and in in vivo ability to reverse reserpine-induced rigidity, which suggests a monoaminergic mechanism with particular importance against Parkinson’s disease [135].

Liu et al. proposed new 3,4-dihydrocoumarin derivatives as potential MAO inhibitors. Pharmacological screening led to the identification of compound **119** (Figure 19, IC_50_ = 0.37 nM) as a promising low nanomolar selective, reversible and competitive MAO-B inhibitor. Furthermore, studies on PC12 cells demonstrated that compound **119** was effective in the protection of dopaminergic neurons against rotenone and 6-OHDA-induced damage, with no significant cytotoxicity. Neuroprotective effects were also evidenced in vivo, with compound **119** being capable to prevent motor deficits in the MPTP-induced PD model, without apparent acute toxicity, with good oral absorption and BBB permeability [125].

**Figure 20:**
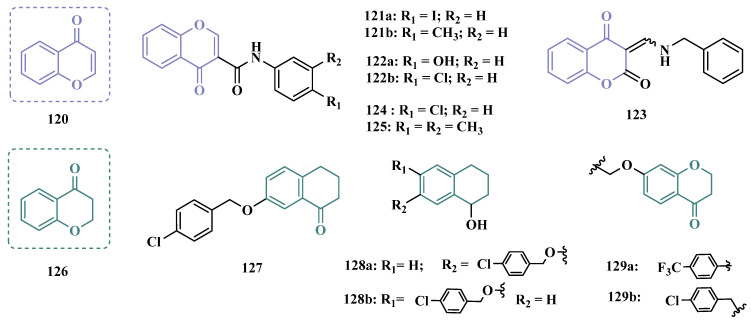
Chemical structures of the chromone **120** and its selective MAO-B inhibitor analogues **121a**,**b**, **122a**,**b**, **123** and **125**; chromone-3-carboxamide derivative **124**, 4-chromanone **126**, 1-tetralone **127**, 1-tetralol derivatives **128a**,**b**, and 4-chromanone **129a**,**b**.

Chromones (**120**, Figure 20) are coumarin isomers widely distributed in nature, with a wide range of biological properties. Due to their large occurrence in plants, chromones are commonly included in the human diet and are less likely to be toxic. Diverse pharmacological properties have been reported for this chemical class, including antibacterial, antifungal, antioxidant, and MAO inhibition [136,137]. Based on these findings, Gaspar and co-workers designed a set of 2- and 3-carboxamide chromone derivatives capable of establishing H-bond interactions with the MAO enzyme. Different substituents were introduced at the *para*-position of the arylamide fragment, and the biological results showed that chromones substituted with a carboxamide at the C3 position of the γ-pyrone nucleus act preferentially as MAO-B inhibitors. Additionally, the nature of the substituents in the arylamide nucleus play a crucial role in the selectivity and affinity for the enzyme isoform. Among all tested compounds, derivatives **121a** (Figure 20, IC_50_ = 0.069 µM) and **121b** (IC_50_ = 0.068 µM) stood out for their selective MAO-B inhibition, showing no activity against MAO-A at 100 µM [138]. Based on their previous results, Gaspar and co-workers proposed two additional series of 2- and 3-carboxamide chromone derivatives, and the biological data corroborated the auxophoric contribution of substituents at the C3 position of the γ-pyrone moiety. As a result, compounds **122a** (Figure 20, IC_50_ = 0.064 µM) and **122b** (IC_50_ = 0.063 µM) were identified as the most active MAO-B inhibitors, acting in a quasi-reversible manner. Regarding selectivity, only **122a** significantly inhibit MAO-A (IC_50_ = 4.76 µM), whereas **122b** showed a selectivity index higher than 1,585 in favor to MAO-B. Notably, despite structural modifications proposed for this new series of 2- and 3-carboxamide chromone derivatives, these most active compounds **122a** and **122b** exhibited the same range of potency that have been already obtained for the parent compounds **121a** and **121b** [139].

Mpitimpiti and co-workers also explored the chromone scaffold in the design and synthesis of a new family of potential MAO inhibitors, seeking to study the effects of introducing more flexible substituents. Several compounds were subjected to in vitro biological screening for their ability to inhibit both MAO isoforms, with chromone-2,4-diones being the most promising core, showing significant MAO-B selectivity. Compound **123** (Figure 20, IC_50_ = 0.638 µM) was highlighted as the most active, reversible and competitive MAO-B inhibitor. Interestingly, molecular docking studies showed that the two geometric isomers of **123** may bind with good affinity to the enzyme cavity and contribute to the inhibition of MAO-B [136].

Following a similar strategy, Cagide and cols. synthesized a set of chroman-2,4-dione and chromone-3-carboxamide derivatives, to obtain new MAO-B inhibitors. In vitro biological data indicated a selective and potent inhibitory profile for hMAO-B. In addition, it was observed that an amide spacer subunit between the chromone system and the benzyl ring, as well as a Cl or CH_3_ substituents in the *para*-position of the phenyl ring, favored inhibitory activity and selectivity. Among the two classes, the chromone-3-carboxamide showed a more pronounced activity against MAO-B, with compound **124** (Figure 20, IC_50_ = 2.9 nM) standing out, showing a low nanomolar 6.7-fold higher potency than the reference drug deprenyl [140].

In previous studies with chromones as potential MAO inhibitors, Reis and co-workers observed that the introduction of a phenylcarboxamide at the C3 position of the γ-pyrone ring resulted in higher MAO-B selectivity [138,139]. To improve the pharmacological profile of chromone-3-phenylcarboxamide-based derivatives, a new set of chromones were synthesized, and biological studies revealed compound **125** (Figure 20, IC_50_ = 0.67 nM) as an impressive reversible and competitive sub-nanomolar MAO-B inhibitor. In addition, this compound did not show significant cytotoxicity towards SH-SY5Y neuroblastoma cells, with predicted favorable physicochemical properties, suggesting adequate BBB permeability, [137].

Derivatives of 1-tetralone (**68**, Figure 12) have been reported as potential MAO inhibitors, as well as 4-chromanones (**126**, Figure 20), which are the corresponding pyranone analogues of **61**. Thus, Cloete and co-workers synthesized and evaluated several new 1-tetralone and 4-chromanone-based derivatives, leading to the identification of 1-tetralone analogue **127** (Figure 20, IC_50 MAO-A_ = 0.036 µM, MAO-B = 1.1 nM), as one of the most promising ligands, showing a nanomolar potency and 32.7-fold higher selective inhibition of MAO-B, being even more potent than the reference selective inhibitors toloxatone (IC_50 MAO-A_ = 3.92 µM) and lazabemide (IC_50 MAO-B_ = 0.091 µM). Among the 1-tetralol series, compounds **128a** (IC_50 MAO-A_ = 0.785 µM) and **128b** (IC_50 MAO-B_ = 7.5 nM) stood out for their potent inhibitory activity of MAO-A and MAO-B, respectively, as well as acting in a competitive and reversible mode for both isoforms. Notably, besides the 1-tetralone analogue **127**, 4-chromanone derivatives also showed a pronounced inhibitory profile of MAO, represented by the selective nanomolar MAO-B inhibitor **129a** (IC_50 MAO-B_ = 3.8 nM) and the selective MAO-A inhibitor **129b** (IC_50 MAO-A_ = 0.286 µM) [141]. These results highlight that the nature and steric hindrance of substituents on the phenyl ring, as well as the regiochemistry, play a crucial role in the selectivity and potency related to both MAO isoforms.

Taking into account that C2 and C3-substituted chromone derivatives have shown selective MAO-B inhibition, and that C6 and C7-substituted congeners have been reported for their activity on both MAO isoforms, Legoabe’s group proposed several new chromone derivatives substituted at the C6 position with alkyloxy substituents, which were rationally selected due to previous results indicating this type of substitution as responsible for increased MAO affinity. Biological evaluation revealed very significant inhibitory activity against both isoforms, especially MAO-B. The best results were observed for derivatives **130a** (IC_50__MAO-A_ = 0.095 µM, MAO-B = 0.33 nM, SI = 287.8) and **130b** (IC_50__MAO-A_ = 0.879 µM, MAO-B = 2.0 nM, SI = 440), which showed a nanomolar potency range with expressive selectivity for MAO-B [142].

**Figure 21:**
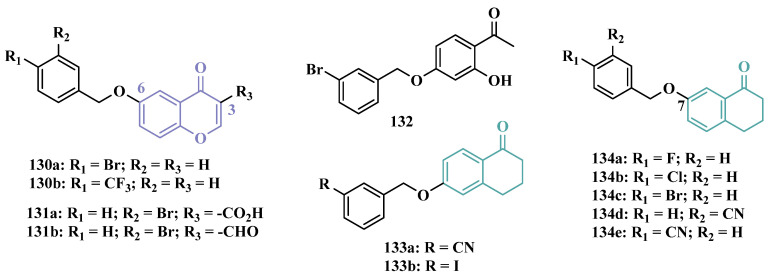
Chemical structures of chromone derivatives **130a**,**b**, **131a**,**b**, and **132**, and the most active α-tetralone derivatives **133a**,**b** and **134a**–**e**.

Literature data have pointed out that variations in the substituents at the C6 position of the chromone core could contribute to the modulation of potency in MAO-B inhibition but lack in selectivity towards the MAO-A isoform. Other evidence has suggested that carboxylic acid substituents at the C3 position of **γ**-pyrone result in potent MAO-B inhibitors, with no effects on MAO-A [143]. Based on these findings, Legoabe’s team designed a new family of chromones substituted at the C3 position of the **γ**-pyrone subunit and the C6 position of the benzo-**γ**-pyrone ring, besides *orto*-acetylphenol analogues of chromone, aiming at the discovery of novel potent and selective MAO-B inhibitors. As a result, compounds **131a** (Figure 21, IC_50__MAO-B_ = 2.8 nM, MAO-A = 1.04 µM), **131b** (IC_50__MAO-B_ = 3.7 nM, MAO-A = 2.20 µM), and **132** (IC_50__MAO-B_ = 4 nM, MAO-A = 15.8 µM) were successfully identified as highly potent and selective MAO-B inhibitors. Moreover, kinetic studies showed that **131a** and **132** act in a quasi-reversible mode of MAO-B inhibition [144]. In another strategy, the same research group considered the structural similarity between the chromone core and α-tetralone in the design of new C6-substituted-α-tetralone architecture, exploring a diversity of benzyloxy, phenylethoxy, and phenylpropoxy substituents aiming at SAR analysis. Pharmacological evaluation led to the identification of 3-nitrile-benzyloxy derivative **133a** as the best MAO-A inhibitor (IC_50 MAO-A_ = 0.024 µM, _MAO-B_ = 0.078 µM), showing higher potency than the reference MAO-A inhibitor toloxatone. Despite its modest 3-fold selectivity for MAO-A, compound **133a** showed significant activity against MAO-B, with potency comparable to the reference compound lazabemide. Conversely, the 3-iodo-benzyloxy analogue **133b** exhibited higher selectivity and a nanomolar potency against MAO-B (IC_50_ = 4.5 nM). Further kinetic studies demonstrated that compound **133a** acts as a reversible and competitive inhibitor of MAO-A, whereas **133b** is a competitive and quasi-reversible MAO-B inhibitor [145] (Figure 21).

Recently, in a continuing effort to identify optimized MAO inhibitors, Legoabe’s group proposed another generation of α-tetralone derivatives, exploring a diverse substitution pattern at the C7 position. Biological results highlighted five potent ligands with a nanomolar range of MAO-B inhibition. Compound **134a** (IC_50__MAO-A_ = 0.012 µM; MAO-B = 0.8 nM) showed the highest sub-nanomolar potency and selectivity (SI = 15) against MAO-B, followed by **134e** (IC_50__MAO-A_ = 0.010 µM; MAO-B = 1.2 nM), **134d** (IC_50__MAO-A_ = 0.026 µM; MAO-B = 3.1 nM), **134c** (IC_50__MAO-A_ = 0.034 µM; MAO-B = 3.5 nM), and **134b** (IC_50__MAO-A_ = 0.033 µM; MAO-B = 4.1 nM), which showed 8 to 9.7-fold lower selectivity in favor of the MAO-B isoform, with an apparent competitive and reversible inhibition mode [146] (Figure 21).

**Figure 22:**
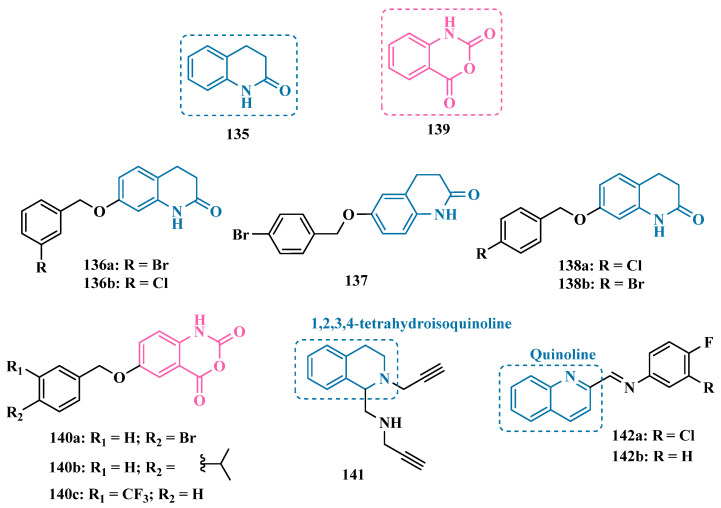
Chemical structures of quinolinone **135** and the leading derivatives 3,4-dihydro-2(1H)-quinolinone **136a**,**b**; quinolinone derivatives **137** and **138a**,**b**, isatoic anhydride (**139**) and its most active derivatives **140a**–**c**, 1-aminomethyl-1,2,3,4-tetrahydroisoquinoline derivative **141**, and halogenated quinoline derivatives **142a**,**b**.

Based on the structural similarity between coumarin and 3,4-dihydro-2(1H)-quinolinone (**135**, Figure 22), Meiring and co-workers designed and synthesized a set of new 3,4-dihydro-2(1*H*)-quinolinone derivatives, aiming at exploring the introduction of alkoxy substituents in the C6 and C7 positions of the coumarin core and their contribution in the selective inhibition of MAO-B. As expected, most of the target compounds exhibited significant selectivity against MAO-B, with derivatives **136a** (Figure 22, IC_50_ = 2.9 nM) and **136b** (IC_50_ = 6.2 nM) standing out as the most potent, acting in a reversible mode, and exceeding the inhibitory potency of lazabemide (IC_50_ = 0.091 μM), used as a reference reversible MAO-B inhibitor. Notably, compound **136b** showed no inhibitory activity against MAO-A, in contrast to **136a** (IC_50__MAO-A_ = 7.98 μM) that was capable of inhibiting this isoform but exhibited a 2751-fold higher selectivity for MAO-B [147]. More recently, in a continued effort to obtain improved MAO inhibitors, Meiring and co-workers proposed a second generation of 3,4-dihydro-2(1*H*)-quinolinones, resulting in 14 new derivatives. Biological studies revealed compounds **137** (IC_50_ = 5.4 nM), **138a** (Figure 22, IC_50_ = 1.4 nM), and **138b** (IC_50_ = 2.5 nM) as the most potent MAO-B inhibitors, with higher potencies than the reference inhibitors lazabemide (IC_50_ = 0.091 µM) and safinamide (IC_50_ = 0.048 µM). Compound **137** was used for enzyme kinetics studies in which it was observed that it acts reversibly and competitively against MAO-B [148].

Isatoic anhydrides (**139**, Figure 22) are considered structurally similar to 3,4-dihydro-2(*1H*)-quinolinones and were explored by Hitge’s group in the design of new MAO inhibitors. Pharmacological studies also included the evaluation of inhibitory effects against cholinesterases and *D*-amino acid oxidase, an enzyme responsible for the degradation of *D*-amino acids that can act as co-agonists in NMDA receptors and whose inhibition could be useful for the treatment of schizophrenia. However, when tested in vitro, the new isatoic anhydride-based derivatives only exhibited significant activity on MAOs, particularly for MAO-A. Compounds **140a** (Figure 22, IC_50_ = 9.5 nM) and **140b** (IC_50_ = 10 nM) were almost equipotent to harmine (IC_50_ = 4.1 nM) and significantly more potent than toloxatone (IC_50_ = 1.64 µM), with good predicted BBB permeability and gastrointestinal absorption. Regarding MAO-B, compound **140c** emerged as the most potent and selective inhibitor (IC_50_ = 4.7 nM), surpassing curcumin (IC_50_ = 2.58 µM) which was used as the reference inhibitor [149].

Based on literature data suggesting that appropriate modifications to the 1,2,3,4-tetrahydroisoquinoline pattern can modulate the activity of MAOs, Guo and co-workers designed and synthesized a set of new 1-aminomethyl-1,2,3,4-tetrahydroisoquinoline derivatives, whose biological evaluation resulted in the identification of compound **141** (Figure 22, IC_50 MAO-A_ = 39.8 µM; MAO-B = 92.3 µM) as the best MAO-inhibitor, but with poor selectivity [150].

Quinoline and its derivatives constitute a structural family with multiple pharmacological applications, recognized for their antibacterial, antihypertensive, anticancer, and MAO-inhibitory potential. Based on this evidence, Oladipo et al. designed and synthesized two quinoline derivatives incorporated into a Schiff base, **142a** and **142b**, aiming to develop new potent MAO inhibitors. Docking and molecular dynamics studies showed that both **142a** and **142b** fit favorably within the MAO-A and MAO-B cavities, establishing predominantly hydrophobic interactions, with **142a** exhibiting higher affinity and stability than **142b**, along with better binding energy. Physicochemical and pharmacokinetic predictions indicated that both derivatives possess favorable properties, complying with Lipinski’s Rule of Five, displaying good lipophilicity, gastrointestinal absorption, and blood–brain barrier permeability. Accordingly, compounds **142a** and **142b** emerged as promising candidates for the development of novel MAO inhibitors, with **142a** standing out for its greater stability and inhibitory potency [151].

**Figure 23:**
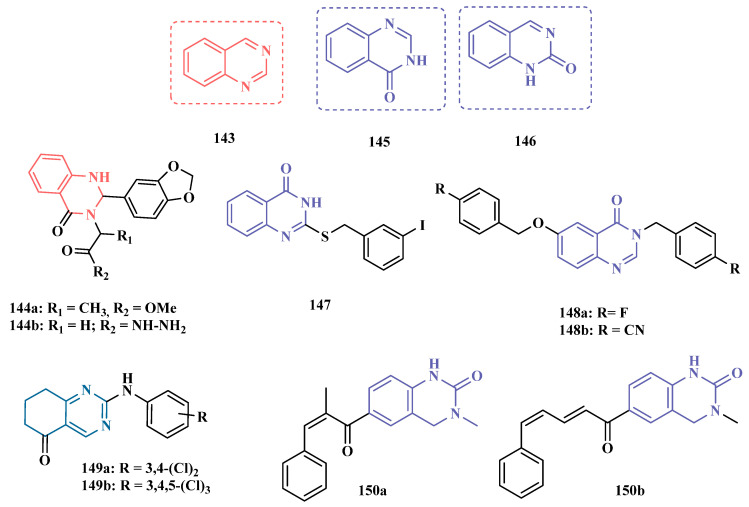
Chemical structures of quinazoline (**143**) and its derivatives **144a**,**b** and **150a**,**b**, 4-quinazolinone (**145**), 2-quinazolinone (**146**) and their most active derivative **147**, 2-(Phenylamino)-7,8-dihydroquinazolin-5(6*H*)-one derivatives **149a**,**b**, and 4(3H)-quinazoline derivatives **148a**,**b** with potent and selective inhibitory effects on MAO-B.

Quinazolinones and their derivatives belong to the alkaloid class and are known for their vast biological and biochemical properties. Consequently, they have been widely studied for the treatment of various diseases, and some approved drugs have already incorporated their structural features into their scaffolds. So, searching for new scaffolds that could result in improved and innovative MAO inhibitors Khattab and co-workers rationally explored the quinazoline core (**143**, Figure 23) in the design of potential MAO-A inhibitor candidates. Among all synthetic derivatives screened for their selective inhibition of MAO isoforms, compounds **144a** and **144b** exhibited the highest low nanomolar inhibitory potencies (IC_50_ = 2.8 and 2.1 nM, respectively), with impressive 30,714- and 39,524-fold higher selectivities for MAO-A, respectively, being comparable to clorgyline (IC_50_ = 2.9 nM, SI = 33,793), used as reference MAO-A inhibitor, with no significant in vivo toxic effects [152].

Besides inhibition of MAO, quinazolinone-based analogues also have inhibitory activity against acetylcholinesterase (AChE), particularly those derivatives containing hydrazine and pyrazoline subunits [153]. In addition, quinazoline nucleus may occur as two possible regioisomers, i.e., 4-quinazolinone (**145**) and 2-quinazolinone (**146**, Figure 23), but the 4-quinazolinone core is the most commonly found in pharmacologically active compounds. Based on this, Qhobosheane et al. proposed new 4-(*3H*)-quinazolinone-based derivatives, functionalized at the C2 position with a thiobenzyl subunit, due to its structural similarity with the benzyloxyl fragment, which could act as bioisosters in potential MAO-B inhibitors. As a result of biological evaluation, derivative **147** (IC_50_ = 0.142 µM) was identified as the best selective, reversible, and competitive MAO-B inhibitor, with a comparable potency of lazabemide (IC_50_ = 0.091 μM) [154]. In another work, the same group synthesized an additional series of 4(*3H*)-quinazolinone derivatives, aimed at evaluating the effect of substituents at the C6 position of the aromatic ring on the MAO inhibition. Surprisingly, the new ligands did not show significant activity against MAO-A, but exhibited a selective moderate inhibitory effect for MAO-B, particularly compounds **148a** (IC_50_ = 0.685 μM) and **148b** (IC_50_ = 0.847 μM, Figure 23), acting as reversible and competitive inhibitors [153].

In the search for novel ligands capable of selectively inhibiting the MAO-B isoform while simultaneously targeting the kinases GSK3β and DYRK1A, enzymes involved in neuroinflammatory processes associated with neurodegenerative diseases, Paolo and colleagues designed a series of 2-(phenylamino)-7,8-dihydroquinazolinone derivatives. Their goal was to optimize the substituent at position 7 of the dihydroquinazolinone core and the phenylamino moiety. The inhibitors **149a** (IC_50 MAO-B_ = 0.004 μM) and **149b** (IC_50 MAO-B_ = 0.008 μM) emerged as the most selective in the series, with **149a** acting as a reversible and competitive inhibitor. In vitro assays showed that these derivatives exhibited no significant cytotoxicity in four human tumor cell lines (A2780, MSTO-211H, HT-29, and HepG2) and displayed blood–brain barrier permeability in PAMPA assays. Additionally, kinase inhibition studies revealed that compound **149a** weakly inhibits GSK3β [155].

Still exploring the quinazolinone structural pattern, Marais and co-workers proposed a new family of 3-methyl-3,4-dihydroquinazolin-2(*1H*)-one derivatives, substituted at the C6 and N1 positions. Despite their close structural similarity, the most active compounds **150a** and **150b** (Figure 23) exhibited an opposed selectivity for the MAO isoforms, with IC_50_ values of 7.43 µM and 0.269 µM for the inhibition of MAO-A and MAO-B, respectively. Moreover, compound **150a** exhibited a comparable potency to toloxatone (IC_50_ = 3.92 µM), an MAO-A inhibitor, and kinetic studies evidenced a reversible and competitive inhibition of this isoform [156].

#### 3.1.8. Benzyloxy-Based Analogues

**Figure 24:**
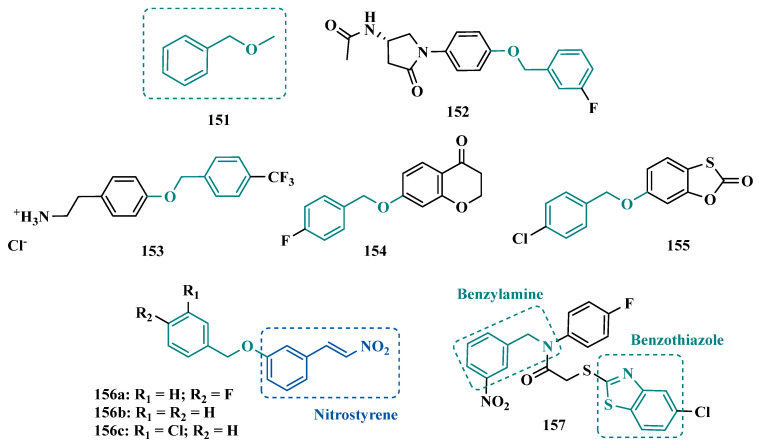
Chemical structures of the benzyloxy subunit (**151**), sembragiline (**152**), benzyloxy analogues **153** and **154**, benzoxathione derivative **155**, 3-benzyloxy-ꞵ-nitrostyrene analogues **156a**–**c**, and the benzothiazole derivative **157** with highlighted selective MAO-B inhibitory activities.

The benzyloxy subunit (**151**, Figure 24) is a common fragment in the structure of potent and reversible MAO-B inhibitors, such as safinamide and sembragiline (**152**, Figure 24). Thus, Yeon and co-workers designed novel 4-(benzyloxy)phenyl and biphenyl-4-yl derivatives to evaluate their pharmacophoric contributions to MAO inhibition. As a result, pharmacological evaluation led to identification of compound **153** (Figure 24) as a promising competitive and highly potent inhibitor of MAO-B (IC_50_ = 9 nM), showing stronger inhibitory activity than the reference inhibitors selegiline (**10**, IC_50_ = 0.625 µM), safinamide (IC_50_ = 0.017 µM), and sembragiline (**152**, IC_50_ = 0.016 µM). In addition, this compound was able to ameliorate biochemical and behavioral imbalances associated with MPTP-induced PD, and significant neuroprotection of dopaminergic neurons against tyrosine hydroxylase [157].

Inspired by the same pharmacophoric moiety, Wang and co-workers synthesized a series of benzyloxy derivatives and evaluated their potential dual neuroprotective and MAO inhibitory activity. Most of the target compounds showed excellent ability and selectivity to inhibit MAO-B, particularly the benzyloxy-tetralone **154** (Figure 24) that exhibited an IC_50_ value of 12.34 nM against MAO-B, with a selectivity index over 8104. In addition, this compound showed balanced neuroprotection in PC12 cells treated with 6-OHDA and rotenone, without significant cytotoxicity, also reducing intracellular ROS, preventing neurotoxin-induced apoptosis, with good permeability in the BBB (PAMPA) and low acute toxicity in vivo [158].

In another approach, Mostert et al. studied a series of benzyloxy-*2H*-1,3-benzoxathiol-2-one to evaluate whether the structural similarity of the benzoxathiole system could result in a bioisosteric contribution to MAO inhibition. Overall, the synthesized compounds showed significant selectivity and potency to inhibit MAO-B. Notably, compound **155** (Figure 24) exhibited a low nanomolar potency (IC_50 MAO-B_ = 3 nM, IC_50 MAO-A_ = 0.189 µM) and a 63-fold higher selectivity in favor of MAO-B isoform, exceeding the potency of the reference drugs toloxatone (**25**, IC_50 MAO-A_ = 3.92 µM), lazabemide (**26**, IC_50 MAO-B_ = 0.091 µM), and safinamide (**12**, IC_50 MAO-B_ = 0.048 µM), [159].

Walt and co-workers also explored the biological contribution of the benzyloxyl subunit in the molecular architecture of new 3-benzyloxy-β-nitrostyrene analogues. Pharmacological studies revealed the moderate selective ability of compound **156a** (Figure 24, IC_50_ = 0.039 µM, SI = 166) in the inhibition of MAO-B, with a comparable potency to selegiline (IC_50_ = 0.020 µM), rasagiline (IC_50_ = 0.070 µM), and safinamide (IC_50_ = 0.080 µM). Further enzyme kinetics studies demonstrated that compound **156a** apparently has a strong binding interaction with the enzyme’s active site and is not readily reversed by dialysis. Regarding MAO-A, derivatives **156b** (IC_50_ = 3.64 µM) and **156c** (IC_50_ = 3.52 µM) were identified as the most potent inhibitors, similar to the reference drug toloxatone (IC_50_ = 3.26 µM) [160].

MedChem is an attractive and common tool among drug design strategies used for exploring the structural similarity of known bioligands. One of the structural subunits commonly present in the scaffold of MAO inhibitors and endogenous amines is benzylamine, whose potential to inhibit MAO has been reported. Therefore, the benzothiazole and benzylamine subunits were included in the new derivatives synthesized by Kaya et al. as MAO inhibitors, resulting in the discovery of compound **157** (Figure 24, IC_50 MAO-A_ = 17.00 µM, MAO-B = 2.95 µM) as a novel MAO inhibitor, acting as a mixed inhibitor, with good BBB permeability, despite its low selectivity [161].

In a similar strategy, Sağlık’s group used the previously studied compound **158** [161]. as a bioactive prototype to propose structural modifications to the design of new benzylamine-sulfonamide derivatives as novel MAO inhibitors. Biological evaluation revealed compounds **159a** (IC_50_ = 0.041 µM) and **159b** (IC_50_ = 0.065 µM) as the most promising selective MAO-B inhibitors, acting by a reversible and non-competitive mode. In addition, these compounds showed no cytotoxicity (IC_50_ > 1000 μM) in the MTT tests carried out on the NIH3T3 cell line, and good predicted pharmacokinetic profile [162].

**Figure 25:**
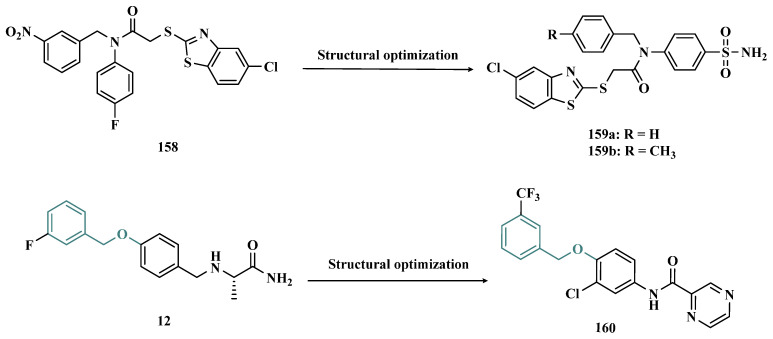
Chemical structures of the new benzylamine-sulfonamide derivatives **159a**,**b** designed from optimization of the derivative **158**, and compound **160** designed as a genuinely optimized drug candidate prototype form the structure of safinamide (**12**).

Safinamide (**12**, Figure 25) is an approved multifunctional drug for PD treatment, acting as a selective MAO-B inhibitor while also preventing glutamate release and dopamine and serotonin reuptake. Despite its clinical efficacy, it can induce hepatic risk and retinopathy, which has been demonstrated in vivo. Taking safinamide as a structural prototype and aimed at optimizing its pharmacological profile, Elkamhawy’s group proposed a novel series of benzyloxyl-pyrazinamide derivatives, resulting in the discovery of compound **160** (IC_50_ = 3.9 nM, SI > 25,641) as a standing out selective and highly potent MAO-B inhibitor, showing a 28.7-fold higher potency than safinamide (IC_50_ = 112 nM; SI > 892), with additional high selectivity. Further in vivo studies evidenced that compound **160** also exhibits neuroprotective effects, reducing MPTP-induced motor dysfunction by oral administration. To determine whether this effect was related to DpA modulation, a tyrosine hydroxylase expression-based assay was carried out, showing that **160** could act in restoring DpA in the SN and striatum, highlighting its therapeutic potential against PD as a multi-functional ligand [47]. 

**Figure 26:**
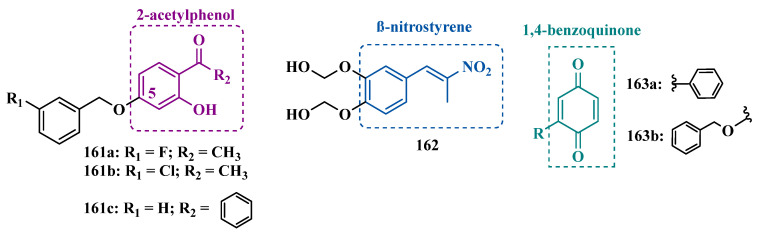
Chemical structure of the 2-acetylphenol derivatives **161a**–**c**, 3,4-bis-oxy-dimethanol-β-methyl-β-nitrostyrene derivative **162** and1,4-benzoquinone derivatives **163a**,**b**.

In previous studies, Legoabe et al. studied a series of 2-acetylphenols for their potential inhibition of MAO-B and observed that substituents at the C5 position favored the inhibitory activity. Thus, further 2-acetylphenols were synthesized, introducing diverse functionalized aromatic substituents at the C5 position. As expected, biological data corroborated the auxophore contribution of the substituted benzyloxy system, as already mentioned in the literature, and led to identification of the halogenated benzyloxy analogues **161a** (Figure 26, IC_50_ = 2.9 nM, SI: 17,482) and **161b** (IC_50_ = 1.3 nM, SI: 13,615) as the most promising nanomolar selective MAO-B inhibitors. Additional modifications in the 2-acetylphenol nucleus were also investigated, revealing compound **161c** (IC_50_ = 4 nM) as another equipotent MAO-B inhibitor, showing a 30-fold higher potency than the reference drug lazabemide (IC_50_ = 0.091 µM) [163].

β-methyl-β-nitrostyrenes have been reported for their wide spectrum of biological properties, including anticancer, antibacterial, and selective enzyme inhibition, especially for phosphatases and telomerases. It is speculated that the nitro-olefin side chain conjugated to the aromatic ring plays a role in their biological activities, and literature data reinforce that the β-nitrostyrene subunit contributes to adequate physicochemical characteristics to overcome the BBB. For this reason, Reis et al. explored a series of new nitrostyrene derivatives to evaluate the pharmacophore contribution of the β-methyl-β-nitrostyrene subunit, also analyzing the structure-activity relationship among different *meta*- and *para*-substituents on the phenyl ring. Biological screening evidenced the 3,4-bis-oxy-dimethanol derivative **162** (IC_50_ = 8.32 µM, SI > 12) as the best selective MAO-B inhibitor [164].

In a different approach, inspired by the structure of quinones that have shown ability to inhibit MAO, Mostert et al. synthesized 4 new monosubstituted 1,4-benzoquinones, including some benzyloxy derivatives. The inhibitory potency of compounds was assessed in vitro, evidencing compounds **163a** (Figure 26) as the most active MAO-A inhibitor (IC_50_ = 5.03 µM) and **163b** (IC_50_ = 3.69 µM), as a selective MAO-B inhibitor. Kinetic studies showed that **163a** act as an irreversible inhibitor, whereas **163b** is a partial reversible inhibitor of the corresponding MAO isoforms [165].

#### 3.1.9. Azole-Based Derivatives

**Figure 27:**
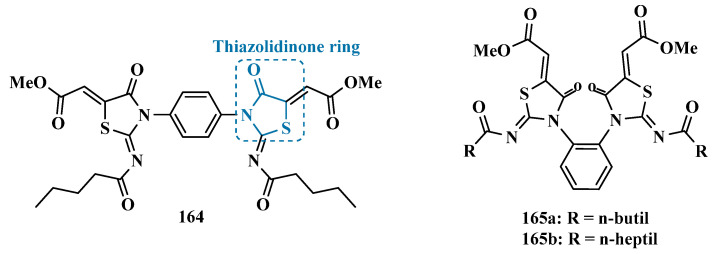
Chemical structures *bis*-iminothiazolidinone derivatives **164**, **165a** and **165b** with selective MAO-A and MAO-B inhibitory activities.

The thiazolidinone nucleus is another privileged structure reported by its pharmacophoric contribution in ligands with a wide spectrum of biological and pharmacological properties. Thus, searching for novel scaffolds with potential MAO inhibition, Abbas and co-workers synthesized *bis*-iminothiazolidinone compounds linked to symmetrical aryl chains. In vitro evaluation stood out the 1,4-aryl-*bis*-thiazolinone derivative **164** (Figure 27, IC_50 MAO-A_ = 1.0 nM) as a highly potent MAO-A inhibitor, with a 4-fold higher potency than clorgyline (**95**, IC_50_ = 4.5 nM). Interestingly, changes in the regiochemistry of the substituents attached to the phenyl ring led to opposed selectivity, as observed for the 1,2-aryl-*bis*-thiazolidinone analogues **165a** (Figure 27, IC_50 MAO-B_ = 0.21 µM) and **165b** (IC_50 MAO-B_ = 0.20 µM) stood out as selective MAO-B inhibitors [166].

**Figure 28:**
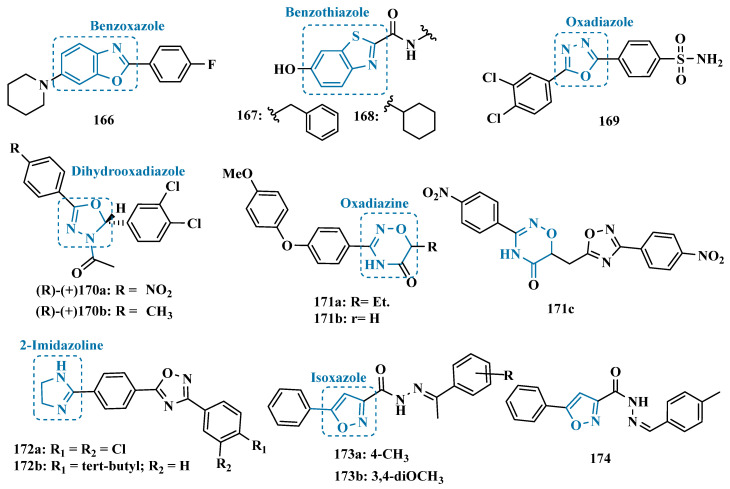
Chemical structures of benzoxazole derivative **166**, 6-hydroxybenzothiazol-2-carboxamides derivatives **167** and **168**, oxadiazole-based derivatives **169** and **170a**,**b**, 1,2,4-oxadiazin-5(6*H*)-one derivatives **171a**–**c**, 2-imidazolines **172a**,**b**, isocarboxazides **173a**,**b** and phenylisoxazole-carbohydrazide derivative **174**.

In previous studies, Sawant’s group had already described benzoxazole and benzothiazole derivatives containing an indole substituent as MAO-B inhibitors and observed that the benzoxazole subunit is crucial for a selective and reversible inhibition [62]. Based on these findings, and seeking to optimize the MAO-B inhibitory and selective profile, a new series of benzoxazole derivatives, substituted with piperidinyl or pyrrolidinyl subunits, was synthesized. Most of the compounds showed selectivity against MAO-B, particularly derivative **166** (Figure 28, IC_50_ = 103 nM), which exhibited the highest potency, comparable to safinamide (**12**, IC_50_ = 51 nM), acting as a reversible and competitive inhibitor [167].

Also inspired by the benzothiazole scaffold, Al-Saad et al. designed and synthesized a series of analogs of compound **167**, a potent and selective MAO-B inhibitor identified through internal library screening. Structural optimizations of the initial derivative revealed that the cyclohexylamide of derivative **168** increased potency 50-fold compared to the benzyl derivative **167**, resulting in the most potent compound of the series, with an IC_50_ of 0.011 μM and competitive inhibition. Furthermore, additional studies showed that the lead compound exhibited significant neuroprotective effects against hyperphosphorylated tau-induced toxicity in SH-SY5Y cells, displayed no cytotoxicity in the same cell line, and demonstrated moderate blood–brain barrier permeability in PAMPA assays [168].

Oxadiazole and sulfonamide derivatives have also been described as excellent MAO inhibitors. Thus, Shetnev et al. explored the combination of these two chemical functionalities to design a series of 5-aryl-1,3,4-oxadiazole-2-ylbenzenesulfonamide derivatives as MAO inhibitors. Biological data evidenced these new compounds as selective for MAO-B, standing out compound **169** (Figure 28, IC_50_ = 2.7 nM) that exhibited a reversible inhibitor of this isoform with a 33-fold higher potency than the reference inhibitor lazabemide (**26**, IC_50_ = 0.091 µM) [169].

The oxadiazole core was also explored by Distinto et al. in the synthesis and evaluation of new derivatives that were designed to study the effects of introducing a 3,4-dichlorophenyl in the C2 position of the dihydroxadiazole system since previous results indicated that a second chlorine atom could contribute to flavine-adenine dinucleotide (FAD) cofactor. In addition, they aimed to study the activity of different enantiomers after molecular docking studies indicated that the *R*-configuration would be preferential for binding to the MAO-B active site. As a result, it was confirmed that both the introduction of a second chlorine atom in the phenyl ring attached to the stereogenic carbon and the *R* configuration led to improved inhibitory potency against MAO-B. Notably, the derivative **170a** (Figure 28, IC_50_ = 7.61 nM) was identified as the most potent ligand; however, it showed unfavorable ADME parameters. In contrast, its methyl-analogue **170b** (IC_50_ = 19.35 nM) showed almost 2-fold weaker potency but possessed much more favorable drug-like properties, including good oral absorption and CNS permeability [170].

In the search for new heterocyclic agents as potential MAO inhibitors, Presnukhina and colleagues synthesized a series of 1,2,4-oxadiazin-5(6H)-one derivatives, which were evaluated for their inhibitory activity. Among the series, analogs **171a** and **171c** were the most potent inhibitors of the MAO-B isoform, with IC_50_ values of 0.900 and 0.371 μM, respectively, whereas for MAO-A, derivative **171b** was the most notable, exhibiting an IC_50_ of 3.75 μM. Kinetic studies demonstrated a competitive inhibition profile for compounds **171a** and **171b** toward MAO-B and MAO-A, respectively [171].

The 2-imidazolines are considered privileged structures due to their ability to bind to various biological targets, their ability to offer bioisosteric relationships to other heterocyclic systems, and the possibility to generate different substituted analogues. Their pharmacological properties are attributed to the ability to bind to imidazoline binding sites, including the I2 site present in MAO, where imidazole ligands can bind in a competitive or non-competitive manner. Thus, Shetnev et al. designed a series of derivatives containing 2-imidazolines as a pharmacophore to access new MAO inhibitors. Overall, the new compounds exhibited selectivity for MAO-B, especially for compound **172a** (IC_50_ = 12 nM, SI = 92), which showed a potency comparable to the reference inhibitors lazabemide (**26**, IC_50_ = 0.091 µM) and safinamide (**12**, IC_50_ = 0.048 µM). Regarding MAO-A inhibition, the most active compound was **172b** (IC_50_ = 0.751 µM). Notably, despite its selectivity for MAO-B isoform, this compound also showed to be a potent MAO-A inhibitor (IC_50_ = 1.11 µM), with a potency in the same order of toloxatone (**25**, IC_50_ = 3.92 µM), a known MAO-A inhibitor [172].

Another noteworthy subunit is isocarboxazid (**50**, Figure 10), a non-selective MAO inhibitor with a polyfunctionalized scaffold consisting of an isoxazole system linked to a hydrazide group. Thus, taking isocarboxazid as a structural prototype, Agrawal et al. proposed a novel series of isoxazole-*N*-acylhydrazones, resulting in the discovery of compounds **173a** (IC_50_ = 5.1 nM, Figure 28) and **173b** (IC_50_ = 6.8 nM), which showed an impressive nanomolar potency to inhibit MAO-B without significant activity toward MAO-A. Further studies revealed that both compounds act as reversible and competitive inhibitors, with good predicted oral bioavailability and BBB permeability. In vivo results showed that these compounds were able to prevent MPTP-induced neurodegeneration, without neurotoxicity and good safety profile (LD_50_ = 2 g/kg) [173]. In another approach, the same group continued to explore the isocarboxazide feature in the design of new phenylisoxazole-carbohydrazides. Interestingly, none of the compounds exhibited significant MAO-A inhibition, and it appears that selectivity for MAO-B depends on increased lipophilicity. Derivative **174** (Figure 28, IC_50_ = 5.3 nM) was identified as the most potent, reversible, and competitive inhibitor of MAO-B, with a predicted good oral bioavailability and BBB permeability. Additional in vivo assays demonstrated its effect on mitigating MPTP-induced motor impairment, without evidence of neurotoxicity, and very low acute toxicity (LD_50_ = 2 g/kg) [174].

**Figure 29:**
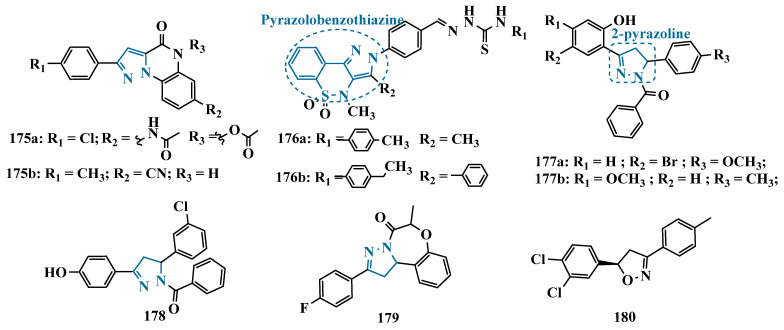
Chemical structures of the pyrazolo[1,5-a]quinoxalin-4-ones derivatives **175a**,**b**, pyrazolobenzothiazine derivatives **176a**,**b**, 2-pyrazoline derivatives **177a**,**b** with MAO-A selectivity, triphenylpyrazoline derivative **178** with multifunctional and selective inhibitory activity of MAO-B, pyrazole derivative **179**, and 3,5-diaryl-4,5-dihydro-isoxazole derivative **180**.

Based on previous reports on MAO inhibitors constituted by a fused tricyclic ring system, Panova and co-workers proposed a new pyrazolo[1,5-a]quinoxalin-4-one scaffold. Biological studies revealed a different selectivity profile against MAO isoforms, with compound **175a** (IC_50_ = 28 nM, Figure 29) identified as the most potent MAO-A inhibitor, but also showing a remarkable activity against MAO-B (IC_50_ = 1.40 µM). In contrast, the analogue **175b** (IC_50_ = 0.617 µM) exhibited a highly selective inhibition of MAO-B, with good in silico-predicted abilities to cross the BBB and gastrointestinal absorption [175].

In another approach, inspired by the structure of pyrazolobenzothiazine and its pharmacological properties, such as its anti-inflammatory, analgesic, and antidepressant potential, Abid et al. synthesized a new series of pyrazolobenzothiazine-based thiocarbazones to identify derivatives **176a** (Figure 29, IC_50_ = 3.0 nM) as a potent and selective nanomolar MAO-A inhibitor, besides derivative **176b** (IC_50_ = 0.02 µM), which exhibited higher selectivity against MAO-B, with a comparable potency to the reference drug deprenyl (**10**, IC_50_ = 0.0196 µM) [176].

The 2-pyrazoline system is a biophore associated with antidepressant activity and MAO inhibitory activity of some bioactive compounds and was explored by Evranos-Aksoz and co-workers to generate racemic 2-pyrazolamide derivatives, which were evaluated in vitro. Biological data highlighted compounds **177a** (Figure 29, Ki = 4 nM) and **177b** (Ki = 5 nM) that exhibited the most pronounced selective MAO-A inhibition in a nanomolar range. Notably, compound **177a** showed a higher potency, but a 126-fold lower selectivity, than moclobemide (**47**, Ki = 10 nM, SI = 0.007), a reference MAO-A inhibitor. Further studies showed that **177a** acts as competitive and reversible inhibitor, without significant cytotoxicity on HepG2 cell lines (MTT test) [177]. The pyrazoline system was also used by Cheng and cols. in the design of a new singular triphenylpyrazoline core. As a result, some ligands were identified as potential neuroprotective agents, but no significant inhibitory of MAO was observed. Notably, the derivative triphenyl-chlorophenolketone pyrazoline **178** (Figure 29) exhibited a multifunctional neuroprotective activity, preventing both 6-OHDA-induced and ROS-induced damage in human neuroblastoma cells (SH-SY5Y), and a significant selective and reversible MAO-B inhibition (IC_50_ = 12.2 µM), despite being about 200-fold less potent than rasagiline [178].

Based on their previous studies, in which 3,5-diaryl-2-pyrazoline-1-ethanone derivatives were identified as potent and selective MAO-A inhibitors, Chen and cols. synthesized new tricyclic pyrazolo[1,5-*d*][1,4]benzoxazepin-5(6*H*)-one derivatives. In this work, it was observed that the intramolecular cyclization of compounds led to a selective inhibition of MAO-B, with compound **179** (IC_50_ = 221 nM, SI = 271) exhibiting the best potency and selectivity, showing a slightly high potency than selegiline (**10**, IC_50_ = 321 nM), which was used as MAO-B reference inhibitor [179].

Taking into account literature data pointing out that the presence of two aryl subunits attached to the dihydro-(1*H*)-pyrazole system appears to be crucial for selectivity and potency against MAO-B [180,181,182,183], Meleddu and co-workers synthesized several diarylisoxadiazole and diarylisoxadiazoline derivatives designed by bioisosteric introduction of an oxygen atom in the dihydro-(1*H*)-pyrazole ring, and exploring different substituents at the phenyl rings. Experimental data revealed new highly potent and selective MAO-B inhibitors [170,184,185,186], encouraging the group to explore other potential 3,5-diaryl-4,5-dihydroisoxazoles as bioisosters. Pharmacological evaluation demonstrated increased selectivity for MAO-B, and Fe^2+^ and Fe^3+^ chelating ability, despite compound **180** (IC_50_ = 11.97 nM) showing the most pronounced nanomolar and selective MAO-B inhibition but lacks metal chelation ability [187].

**Figure 30:**
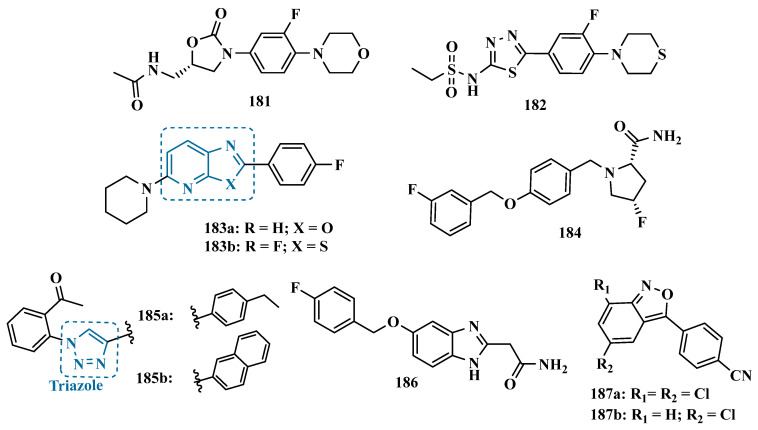
Chemical structures of the thiazolopyridine derivatives **181**; structure of the linezolid (**182**) and its derivative **183** pyrrolidine derivative **184** and keto-triazole derivatives **185a** and **185b**, benzimidazole derivative **186**, and 2,1-benzisoxazole derivatives **187a**,**b** with pronouncing activity against MAO-B.

Linezolid (**181**, Figure 30) is an oxazolidinone antibiotic widely used for its antimicrobial activity against resistant strains of *Mycobacterium tuberculosis*. However, studies have shown that this compound is associated with serotonergic toxicity, particularly serotonin syndrome, as it can inhibit MAO. In this context, the Dhangar group aimed to explore the pharmacological properties of linezolid by designing and synthesizing a series of new analogs through a “toxicity-to-activity optimization” strategy, seeking to develop derivatives that retain MAO-B inhibitory potential while eliminating antibacterial activity. The lead compound **182** demonstrated potent MAO-B inhibition, with an IC_50_ of 0.03 μM, showing improved inhibition and selectivity compared to linezolid. Enzyme kinetics evaluation revealed that analog **182** acts as a reversible and competitive MAO-B inhibitor [188].

Oxazolopyridine derivatives with aryl and heteroaryl substituents have been used as MAO-B inhibitors, but other similar scaffolds, such as thiazolopyridines, have not been studied for this purpose. Thus, Park’s group synthesized and evaluated new oxazolopyridine and thiazolopyridine derivatives for their inhibitory capacity against human MAO-B. As a result, within the 2-phenyloxazolopyridine series, derivative **183a** (Figure 30, IC_50_ = 1.373 mM) exhibited the best activity against MAO-B, though with very low potency in the millimolar range. In contrast, the thiazolopyridine series yielded compound **183b**, which demonstrated significantly higher inhibitory activity (IC_50_ = 26.5 µM) and selectivity for MAO-B [189]. Based on previous results, Wang and cols. recently synthesized and evaluated a series of new chiral fluorinated pyrrolidine derivatives in vitro and in vivo. The structural design involved modifying the *N*-heterocycle fragment by adding different chiral substituents, resulting in the identification of compound **184** (Figure 30), which not only showed a pronounced and selective inhibition of MAO-B (IC_50_ = 0.019 µM, SI = 2,440), surpassing the reference inhibitor safinamide (**12**, IC_50_ = 0.163 µM, SI = 172), but also exhibited excellent metabolic and pharmacokinetic parameters in animals. The superior activity of compound **184** compared to safinamide, observed in vitro, was also maintained in vivo. It was capable of reducing MPTP-induced DpA deficits in a PD model in rats, enhancing the effect of levodopa on the concentration of DpA in the striatum, and significantly reducing galantamine-induced jaw tremors in animal models [190].

Triazole is another heterocyclic substructure widely explored in MedChem, being part of the structural architecture of several drugs, such as anticonvulsants and antibiotics. In addition, this biophore has been reported as playing a crucial role in the biological properties of numerous compounds studied as potential neuroprotective, antinociceptive, and anti-inflammatory agents. Thus, Costa and co-workers performed in silico, synthetic, and biological studies with a family of 2′-(1,2,3-triazol)-acetophenones, leading to the identification of compounds **185a** (IC_50 MAO-A_ = 2.64 µM, Figure 30) and **185b** (IC_50 MAO-B_ = 41.47 µM), as interesting selective MAO-A and MAO-B inhibitors, respectively [191].

Structure–activity relationship studies indicate that the presence of an amide bond in benzo N-heterocyclic scaffolds is critical for establishing key interactions within the MAO-B active site, while the incorporation of hydrophobic groups enhances both potency and selectivity. In this context, Lv and colleagues developed two series of benzimidazole derivatives, in which the scaffold was linked via an amide bond to a substituted hydrophobic phenyl group. Among these compounds, **186** emerged as the most potent hMAO-B inhibitor, with an IC_50_ of 64.3 nM, comparable to safinamide (12, IC_50_ = 42 nM), acting as a reversible and competitive inhibitor. In vitro assays demonstrated that compound **186** was non-cytotoxic in PC12 cells and capable of reducing H_2_O_2_-induced oxidative damage. Furthermore, in vivo studies revealed favorable pharmacokinetic properties, a good safety profile in cellular toxicity assays, and effective blood–brain barrier permeability in a murine MPTP-induced Parkinson’s disease model. Notably, **186** significantly alleviated motor impairment, particularly muscle relaxation and coordination, in mice [192].

Aiming to develop new MAO inhibitors, Shetnev et al. synthesized a series of 2,1-benzisoxazole derivatives, evaluating the impact of various substituents and substitution patterns on structure–activity relationships. The most potent MAO-B inhibition was observed for derivatives **187a** (IC_50_ = 0.017 μM) and **187b** (IC_50_ = 0.098 μM), both substituted with p-benzonitrile on the isoxazole ring. Kinetic studies further revealed that derivative **187a** acts as a reversible and competitive MAO-B inhibitor [193].

#### 3.1.10. Diverse Molecular Hybrids

**Figure 31:**
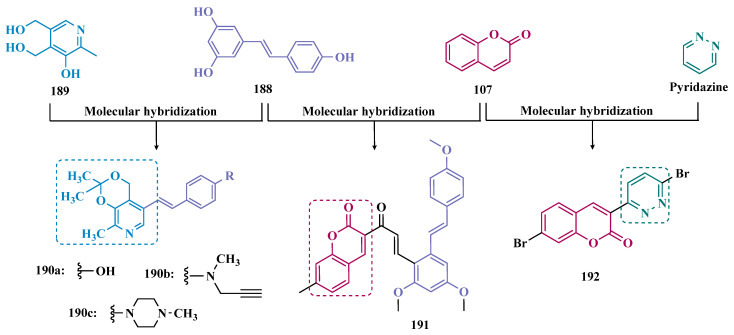
Representation of the rational design of molecular hybrids based on the structure of resveratrol (**188**) and pyridoxine (**189**) leading to derivatives **190a**–**190c**; resveratrol (**188**) and coumarin (**107**) generating derivative **191**, and coumarin (**107**) and pyridazine to furnish the pyrido-coumarin derivative **192**.

Resveratrol (**188**, Figure 31) is a polyphenol found in plants, such as red grapes, blackberries, and mulberries, and its vast biological properties have been intensively studied in the last 2 decades, including antioxidant, anti-inflammatory, anti-apoptotic, and neuroprotective effects. In addition, some studies reported resveratrol derivatives as potential MAO-B inhibitors [194,195,196]. On the other hand, pyridoxine (**189**, Figure 31), is an enzyme cofactor that can act as an antioxidant and prevent radical production. Based on this information and continuing previous studies, Li et al. synthesized a series of pyridoxine-resveratrol hybrids aimed at inhibiting MAOs and neuroprotection. Biological evaluation revealed that **190a** (Figure 31, IC_50_ = 0.01 µM), **190b** (IC_50_ = 0.01 µM) and **190c** (IC_50_ = 0.02 µM) were the most promising compounds, with selective and significant inhibitory potencies against MAO-B. Kinetic studies demonstrated that compounds **190a** and **190c** act as reversible inhibitors, whereas **190b** is an irreversible one. In addition, these compounds exhibited neuroprotective effect, being able to reverse H_2_O_2_-induced neuronal damage by 20% in PC-12 cells, with no cytotoxicity at a concentration of 10 µM, assessed using the MTT assay. They also showed antioxidant capacity and the ability to cross the BBB, which indicates good druggability properties [197].

Coumarin (**107**, Figure 31) is a promising structure for the development of new drugs, given the wide range of biological properties presented by its derivatives, including [198]. Thus, inspired by the structure of resveratrol and coumarin, Ruan and cols. proposed a new hybrid structural architecture, potentially suitable for MAO inhibition. Biological results evidenced compound **191** (IC_50_ = 2.78 µM, SI = 20.93) for its selectivity for MAO-B and significant low-micromolar activity, being equipotent to the reference drug selegiline (**10**, IC_50_ = 2.89 µM). Interestingly, SAR analysis suggested that bulky substituents at the C7 of the coumarin core favor MAO-A inhibition, while smaller substituents appear to favor MAO-B selectivity. Further in vivo experiments indicated a very low toxicity (LD_50_ > 1000 mg/kg) for compound **191** [199].

In another strategy, inspired by studies indicating that 3-heteroarylcoumarins with a pyridazine moiety at the C3 position resulted in excellent MAO-B inhibitors with a good pharmacological profile, Rodríguez-Enríquez and co-workers synthesized a family of 3-pyridazinylcoumarin hybrids. The authors hypothesized that this new scaffold could modify polarity and improve the lipophilicity of coumarin, thereby enhancing its interaction with the enzyme. In vitro evaluation against both MAO isoforms stood out compound **192** (IC_50_ = 0.06 µM), with a comparable potency to selegiline (IC_50_ = 0.02 µM), with high selectivity and reversible mode of MAO-B inhibition. This compound also showed no toxicity in SH-SY5Y cells. Further in vivo assays demonstrated that compound **192** was also able to enhance the L-dopa/benserazide effects on motor activity, with no evident toxicity and an adequate druggability profile [198].

**Figure 32:**
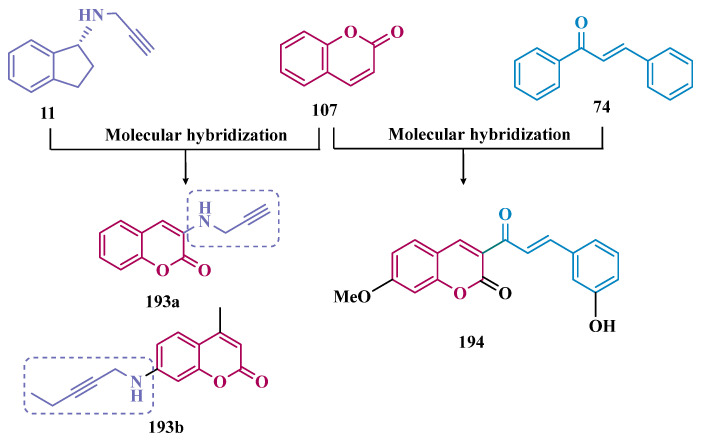
Representation of molecular hybridization between rasagiline (**11**) and coumarin (**107**), resulting in the compounds **193a**,**b**, and between coumarin (**107**) and chalcone (**74**) to furnish the 3-hydroxy-cinamoyl-coumarin **194**, with selective MAO-B inhibition.

Also using the coumarin core as a structural model and considering that rasagiline (**11**) is a potent irreversible MAO-B inhibitor, Matos and cols. synthesized new coumarin-rasagiline hybrids as potential MAO inhibitors. Compound **193a** (IC_50 MAO-B_ = 0.95 µM, Figure 32) stood out as the most potent derivative, followed by its superior homologue **193b** (IC_50 MAO-B_ = 3.97 µM). Both acted as selective and partially reversible MAO-B inhibitors, also exhibiting antioxidant and neuroprotective properties [200].

Literature data suggest that appropriate substitutions in the coumarin scaffold could enhance selectivity and affinity on MAO inhibition. Moreover, coumarin and chalcones (**74**) are known for their broad spectrum of biological activity, including neuroprotection. Therefore, Moya-Alvarado et al. proposed the molecular hybridization of these two pharmacophores to generate potential MAO inhibitors with an innovative structural feature. Biological screening of these new analogues revealed that they were active only against MAO-B isoform. In particular, compound **194** (IC_50_ = 0.76 μM) exhibited the highest inhibitory potency, along with good solubility and high gastrointestinal absorption [201].

**Figure 33:**
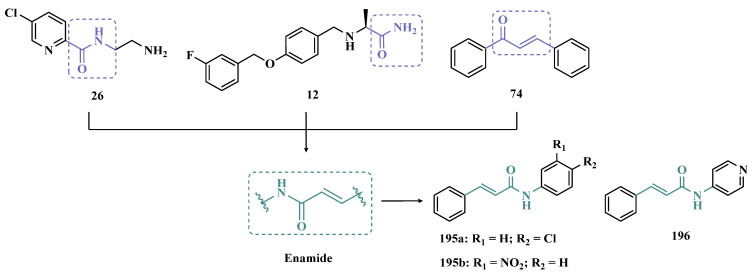
Structural representation of the resulting enamide hybrids **195a**,**b** and **196** designed by molecular hybridization of the structures of lazabemide (**26**), safinamide (**12**), and chalcone (**74**).

Recent literature suggests that the presence of a lone electron pair on the nitrogen atom of enamides enhances the nucleophilicity of Cγ due to its delocalization through the α,β-unsaturated ketone subunit (Figure 33). Additionally, studies have shown that MAO inhibitors should contain at least one hydrophobic ring, a H-bond donor and a H-bond receptor in their structures. Based on these findings, Kavully et al. proposed combining the amide substructures of lazabemide (**25**) and safinamide (**12**) with the conjugated ketone moiety of chalcone to generate an enamide scaffold featuring two hydrophobic aromatic rings connected to an H-bond acceptor/donor enamide subunit. As a result, compounds **195a** (IC_50_ = 0.11 µM, SI > 363.3, Figure 33) and **195b** (IC_50_ = 0.10 µM) were identified as the most potent, selective, reversible and competitive MAO-B inhibitors, with no cytotoxicity towards VERO cell line. In contrast, the 4-amidopyridine derivative **196** (IC_50_ = 5.95 µM) exhibited the best potency and selectivity against MAO-A [202].

Inspired by thiosemicarbazone derivatives, which have demonstrated the ability to inhibit human MAO at low concentrations, and benzofuran/benzothiophene-based pharmacophores, which appear to contribute to MAO inhibition, Osmaniye et al. synthesized molecular hybrids of these two biophore subunits. In vitro biological evaluation identified compounds **197a** (IC_50 MAO-B_ = 42 nM) and **197b** (IC_50 MAO-B_ = 56 nM) as highly potent and selective MAO-B inhibitors, with comparable potencies to the reference drug selegiline (IC_50_ = 37 nM). Moreover, both compounds exhibited no cytotoxicity in MTT assays on the NIH/3T3 cell line, and kinetic studies revealed their reversible and non-competitive mechanism of enzymatic inhibition [203].

**Figure 34:**
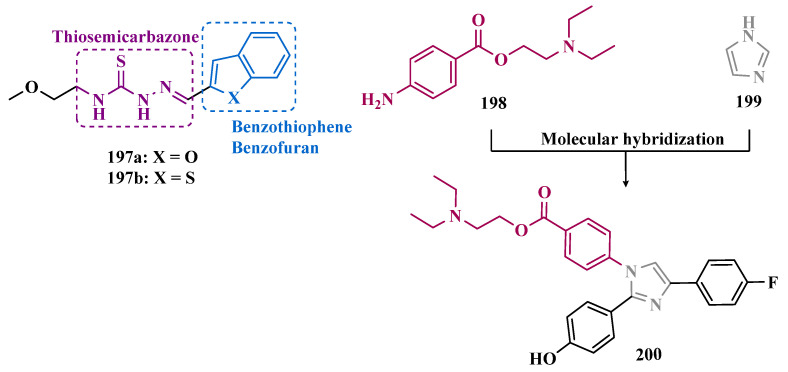
Chemical structures of the molecular hybrids benzofuran-thiosemicarbazone **197a** and benzothiophene-thiosemicarbazone **197b**, procaine-imidazole **200**, with selective activity against MAO-B.

Procaine (**198**, Figure 34) and imidazole (**199**) are two additional structural scaffolds that have been explored in the design of MAO inhibitors. Procaine, in particular, has been reported as a reversible and competitive MAO inhibitor. Thus, in pursuit of novel and improved MAO inhibitors, Wu et al. synthesized a series of procaine-imidazole hybrids and identified compound **200** (IC_50 MAO-A_ = 15.22 µM; MAO-B = 0.032 µM) as a highly selective MAO-B inhibitor (SI = 475). Further in vivo pharmacological studies demonstrated a good safety profile, with no acute oral toxicity at the maximum dose of 2000 mg/kg, and no neurotoxic effects. In addition, compound **200** was able to counteract MPTP-induced motor impairment in rats and improve antioxidant enzymes levels in the striatum [204].

**Figure 35:**
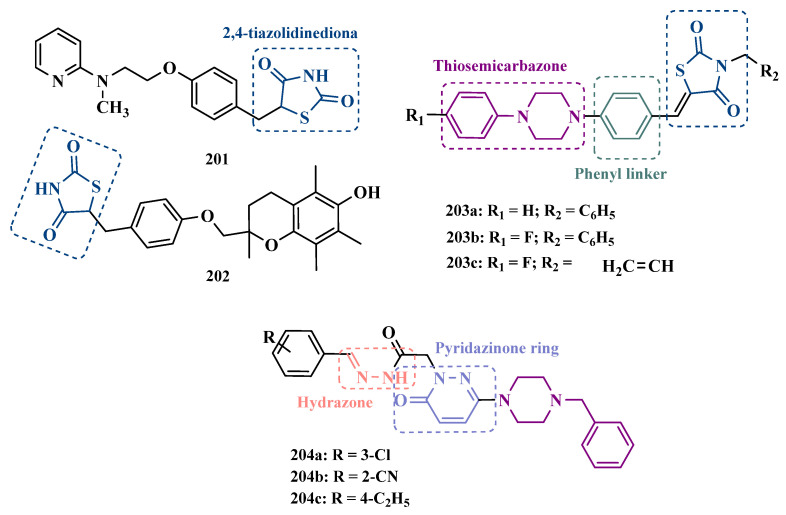
Chemical structures of the rosiglitazone (**201**) and troglitazone (**202**), molecular hybrids phenylpiperazine-thiazolidine-2,4-dione **203a**–**c**, and pypidazinobenzylpiperidine derivatives **204a**–**c**.

Piperazine is a heterocyclic structural scaffold with a wide range of pharmacological properties. Numerous piperazine derivatives have been reported in the literature as effective psychoactive agents, while many have also been investigated for MAO inhibitory potency. Concurrently, 2,4-thiazolidinedione has emerged as a structural motif in various biological profiles and is present in drugs such as rosiglitazone (**201**) and troglitazone (**202**, Figure 35), described as inhibitors of both MAO isoforms. Building on this, and in continuation of previous work, El-Halaby et al. designed new hybrid compounds by fusing phenylpiperazines with 2,4-thiazolidinedione moieties, connected via a lipophilic phenyl spacer (Figure 35), aiming to increase compound lipophilicity and thereby optimize pharmacokinetics and CNS bioavailability. The most active compounds for hMAO-A were hybrids **203a** and **203b**, with IC_50_ values of 0.194 and 0.188 μM, respectively, whereas for hMAO-B, compound **203c** exhibited the most potent inhibitory activity, with an IC_50_ of 0.330 μM. Analog **203b** was further evaluated for enzyme kinetics, showing a mixed-type inhibition, and for cytotoxicity in normal SH-SY5Y cells and neuroprotective activity in the MTT assay in PC12 cells, demonstrating non-cytotoxicity and favorable antioxidant properties. Finally, ADME profiles of the most active hybrids, **203a** and **203b**, indicated good CNS permeability, supporting their potential as successful drug candidates [205].

Pyridazinone and its derivatives constitute a class of compounds with reported anti-inflammatory, anticancer, antioxidant, and antihypertensive activities. Based on this premise, Jong Min Oh and colleagues synthesized and evaluated a series of benzylpiperidinopyridazinone analogs. These derivatives can be described as hybrid molecules resulting from the combination of pyridazinone, benzylhydrazone, and substituted phenyl scaffolds (Figure 35), strategically designed to optimize pharmacokinetic properties. Among the series, compounds **204a** and **204b** showed the most potent MAO-B inhibition, with IC_50_ values of 0.203 and 0.979 μM, respectively, whereas for MAO-A, compound **204c** (IC_50_ = 3.691 μM) was the most promising. Furthermore, derivatives **204a** and **204b** exhibited reversible and competitive inhibition and demonstrated good blood–brain barrier permeability in PAMPA assays [206].

**Figure 36:**
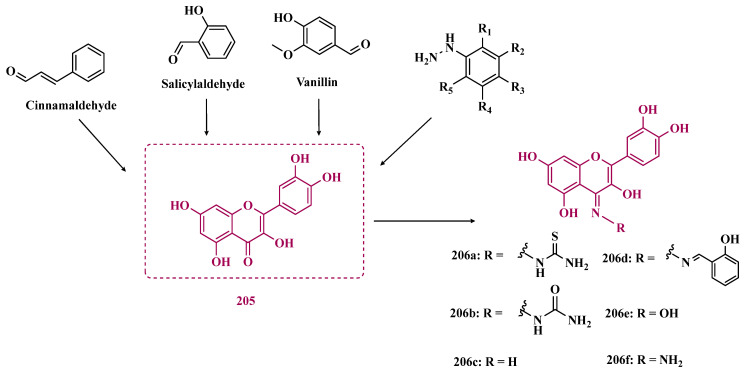
Representative use of quercetin’s structure and other small natural molecules in the design of new hybrid MAO inhibitors **206a**–**f**.

Dhiman et al. studied a series of hybrid quercetin (**205**, Figure 36) derivatives, considering that their structural scaffold could represent a natural hybrid of several bioactive small molecules such as cinnamaldehyde, salicylaldehyde and vanillin. Moreover, quercetin is reported by its broad spectrum of biological properties, including antioxidant, neuroprotective, anti-inflammatory and MAO-A inhibition effects, corroborated by in vitro, pre-clinical, molecular docking and SAR studies. The synthetic quercetin derivatives were obtained by introduction of various functional groups at the C4 position of quercetin structure, leading to the identification of semicarbazides **206a** (IC_50_ = 12.70 μM), **206b** (IC_50_ = 18.87 μM), and imine **206c** (IC_50_ = 19.56 μM) as the most potent and selective MAO-B inhibitors. In contrast, derivatives **206d** (IC_50_ = 13.10 μM), **206e** (IC_50_ = 16.03 μM), and **206f** (IC_50_ = 19.36 μM) exhibited their highest inhibitory activity within the same potency range, but with opposite selectivity for MAO-A. Furthermore, compounds **206a**, **206b** and **206f** exhibited high antioxidant activity, being able to counteract H_2_O_2_-induced cellular oxidative stress [207].

Overall, the studies reviewed in this section demonstrate that diverse chemical scaffolds, particularly indoles, indazoles, hydrazones, phthalimides, indanones, and chalcones, have yielded highly potent and selective MAO-B inhibitors with favorable pharmacokinetic properties and neuroprotective effects, reinforcing the relevance of this isoform as a strategic target in the development of novel therapies for PD.

### 3.2. Dual and Multi-Target MAO Inhibitors

Multitarget inhibitors are rationally designed molecules intended to modulate multiple pathological targets simultaneously. These compounds have emerged as a promising concept to address the multifactorial nature of neurodegenerative diseases, as they can combine MAO activity regulation with complementary actions such as cholinesterase inhibition, adenosine or histamine receptor modulation, and antioxidant effects. Generally, the design of multitarget ligands can involve: (i) the combination of two or more pharmacophores, each acting on biochemical pathways underlying neurodegeneration or (ii) the synthesis of a single molecule exhibiting promiscuous activity, interacting with multiple targets influencing disease progression [208]. This approach aims to enhance therapeutic efficacy by acting on different neurotransmitter pathways, reduce the need for multiple drugs and, consequently, minimize drug–drug interactions and toxicity, while improving adherence and quality of life [209].

#### 3.2.1. Dual Histamine Receptor Modulators and MAO Inhibitors

Histamine H3 receptors (H3R) belong to the G protein-coupled receptors family and are widely expressed in the brain, particularly in regions associated with cognitive processes. These receptors regulate the release of neurotransmitters beyond histamine, such as DpA and ACh, increasing their levels in the synaptic cleft. Thus, studies suggest that blocking this receptor could enhance the treatment of various diseases, including PD [210,211].

**Figure 37:**
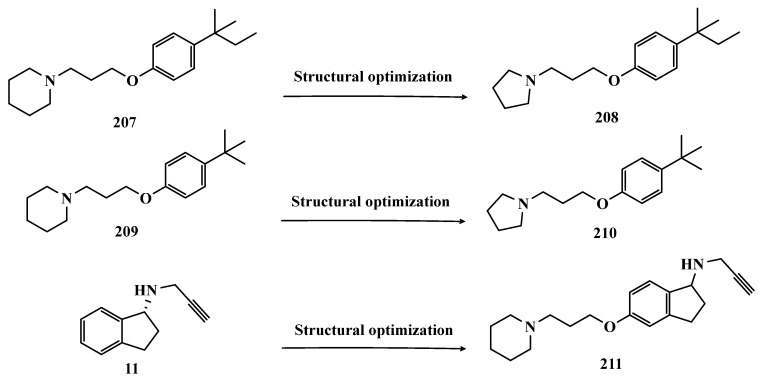
Representation of strategies used to obtain the optimized dual MAO-B and H3R antagonists **208**, **210**, and **211** from the corresponding precursor prototypes **207**, **209**, and **11**, respectively.

In previous works, Łażewskaa et al. described compound **207** (Figure 37) as a high-affinity ligand for the H3R in vitro and in vivo [212], with pro-cognitive effects [213] and anticonvulsant activity [214]. Based on these results, the authors synthesized new analogues of **207** with different alkyl and heterocyclic amines, aiming to optimize their affinity for H3R and their ability to inhibit MAO-B. In general, all new analogues showed higher affinity for H3R and greater effectiveness in the selective inhibition of MAO-B, particularly compound **208** (Figure 37), which showed Ki_H3R_ = 63 nM and IC_50 MAO-B_ = 4.5 nM, demonstrating a non-competitive and reversible inhibition of MAO-B [211]. More recently, the same group explored the structure of compound **209** (Figure 37), which is a high-affinity H3 ligand (Ki = 38 nM) and a potent and selective MAO-B inhibitor (IC_50_ = 48 nM), as a structural prototype for the design of new 4-*tert*-butylphenoxyl analogues (Figure 37). As a result of pharmacological evaluation, none of the compounds showed higher affinity for the H3R than **209**. However, despite its low H3R affinity (Ki = 371 nM), compound **210** was identified as a selective and low-nanomolar MAO-B inhibitor (IC_50_ = 2.7 nM), surpassing the potency of the reference inhibitors rasagiline (**11**, IC_50_ = 15 nM) and safinamide (**12**, IC_50_ = 7.7 nM). Kinetic studies demonstrated that both compounds act as reversible inhibitors of MAO-B, with analogue **209** showing a mixed-type mode of inhibition. Additionally, compound **209** exhibited the best dual activity against both targets of interest; however, no neuroprotective effect against H_2_O_2_ was observed in the SH-SY5Y cell line, while showing no cytotoxicity against HEK-293 and SH-SY5Y cells. Further in vivo studies demonstrated that compound **209** was also able to reduce the duration of haloperidol-induced catalepsy [107].

In another approach, aiming to develop compounds with a multifunctional profile, Lutsenko et al. developed new rasagiline derivatives by introducing a benzyloxy-alkylamine subunit as an H3 receptor antagonist pharmacophore. In their rational design, the authors proposed a new hybrid rasagiline structure fused to a 3-piperidinopropyloxy subunit, leading to derivative **211**, which exhibited the most promising dual activity against MAO-B (IC_50_ = 256 nM) and hH3R (Ki = 2.6 nM). This compound acted as an irreversible MAO-B inhibitor, with low significant cytotoxicity in neuronal cells [210].

#### 3.2.2. Dual Cholinesterase and MAO Inhibitors

**Figure 38:**
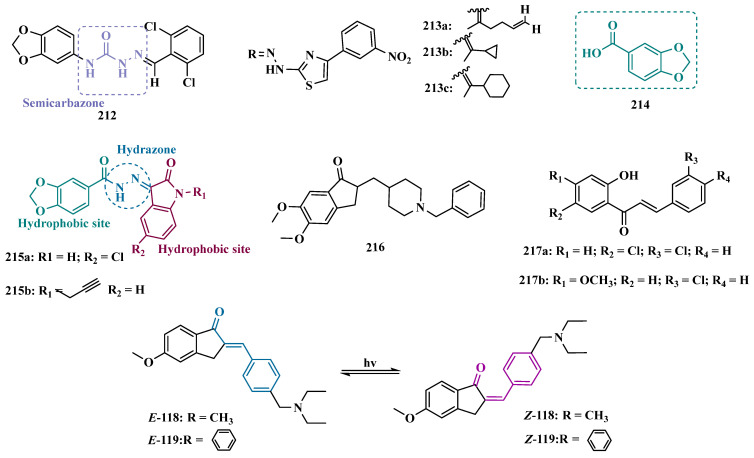
Chemical structures of the piperonyl-semicarbazone derivative **212** and hydrazone derivatives **213a**–**213c** with dual inhibitory activity of MAO-B and AChE; piperonyl acid (**214**), piperonyl-acyl-hydrazones **215a**,**215b** and donepezil (**216**); 2-hydroxychalcone derivatives **217a**, **217b**; and E/Z 1H-inden-1-one derivatives **218** and **219** with multifunctional activity against MAO-B and ChEs.

Semicarbazones have been reported as promising scaffolds for drug candidates targeting NDs, being suitable for diverse modifications at the amine and imine terminals to enhance pharmacokinetic and pharmacodynamic properties. Moreover, their inhibitory activity of MAOs and AChE has been previously reported. With this in mind, Tripathi et al. synthesized 3,4-(methylenedioxy)aniline semicarbazone derivatives, intending to develop multitarget compounds for MAOs and AChE. Among the compounds synthesized, the piperonyl-semicarbazone derivative **212** (Figure 38, IC_50 MAO-A_ = 4.52 µM, MAO-B = 0.059 µM, AChE = 0.0087 µM) stood out for its more balanced multitarget profile, acting competitively and reversibly for both MAO isoforms and in a mixed manner for AChE [215].

Inspired by the structure of hydrazones, Carradori et al. synthesized a series of new 4-(3-nitrophenyl)thiazol-2-ylhydrazones designed as multitarget inhibitors of MAOs and ChEs, with additional antioxidant potential. The synthesized compounds were evaluated in vitro, leading to identification of compounds **213a** (Figure 38, IC_50_: MAO-A = 3.99 µM; MAO-B = 0.101 µM), **213b** (IC_50_: MAO-A = 2.66 µM; MAO-B = 0.0053 µM), and **213c** (IC_50_: MAO-A = 29.1 µM; MAO-B = 0.0072 µM), which exhibited the best dual inhibitory activities, showing selectivity for MAO-B and AChE. Regarding AChE inhibition, these compounds were capable to inhibit 47%, 44%, and 41% of the enzyme activity, respectively, at a concentration of 3 µM. Additionally, these compounds exhibited in vitro antioxidant activity comparable to that of trolox, which was used as reference compound. They also demonstrated good predicted oral absorption, and adequate ability to penetrate the BBB [216].

Similarly, Vishnu et al. designed new hydrazone derivatives designed as structural hybrids of piperonyl acid (**214**, Figure 38) and Isatin (**54**). Isatin is a notable bioactive compound with significant pharmacological properties, particularly in neuroprotection, making it relevant for the treatment of CNS-related diseases, such as AD and PD. It has been reported as a selective MAO-B inhibitor, and it has a well-accepted pharmacological profile in humans. In their rational design, the authors incorporated a hydrazone subunit to introduce an electron-rich fragment capable of forming H-bond interactions, along with hydrophobic aryl groups attached to the carbimine and amide terminals of the hydrazone moiety. In vitro results evidenced **215a** (IC_50_: AChE = 0.052 µM; BuChE = 0.96 µM; MAO-A = 5.164 µM; MAO-B = 0.89 µM) and **215b** (IC_50_: AChE = 0.85 µM; BuChE = 0.88 µM; MAO-A = 1.73 µM; MAO-B = 0.034 µM) as the most promising compounds, whose values for BuChE inhibition were comparable to those of donepezil (**216**, Figure 38, IC_50_ = 0.78 µM). Both compounds also inhibited AChE in a reversible and competitive manner, while inhibiting MAO-B reversibly. Notably, compound **191b** exhibited the highest selective potency against MAO isoforms, particularly MAO-B, with a potency comparable to that of selegiline (IC_50 MAO-B_ = 0.02 µM) [217].

In another approach, Kamecki’s group explored the structure of chalcones in designing a series of 2-hydroxychalcones, which were evaluated for their multifunctional ability to concomitantly inhibit MAOs, ChEs, βA1-42 aggregation, as well as their ability to bind to benzodiazepine receptors. Compounds **217a** and **217b** (Figure 38) stood out for their optimal multi-target profile, exhibiting significant reversible in vitro inhibition of MAO-B with IC_50_ values of 0.084 µM and 0.111 µM, respectively. These results were confirmed in vivo, with no observed cytotoxicity against neuronal cells. Notably, compound **217b** also demonstrated the ability to inhibit AChE (IC_50_ = 15.17 µM) and βA1-42 aggregation (75.7%) in vitro. Further assays revealed its good affinity (Ki = 5.0 µM) for the benzodiazepine site of γ-aminobutyric acid (GABA) receptors, leading to sedative effects in rats [95].

Photopharmacology involves the use of light as an external stimulus to precisely modulate the activity of pharmacologically relevant molecules, employing two main strategies: (1) conjugation of the bioactive structure with a photolabile caging group that can be released in situ upon light irradiation and (2) conjugation with well-characterized molecular photoswitches. Based on this approach, Paolino et al. developed a small library of cinnamic acid analogs with light-modulated activity, aiming to synthesize multitarget derivatives capable of selectively inhibiting AChE and MAO-B, while exhibiting reduced activity toward BuChE and MAO-A. The group analyzed the propensity of 5-methoxy-indanone derivatives to undergo isomerization upon UV-B irradiation, confirming via UV–Vis spectra the *E*-to-*Z* diastereoisomer interconversion without degradation during the photoreaction. Furthermore, the *E* diastereoisomers displayed good inhibitory activity against AChE and MAO-B, particularly influenced by methoxy substituents. Among these molecules, compound **218** (Figure 38) exhibited notable inhibitory activity, with IC_50_ values of 0.113 μM and 0.26 μM for AChE and MAO-B, respectively. Upon UV-B irradiation, the *E* isomers converted into *E/Z* racemic mixtures, resulting in a significant reduction of activity, as the *Z* isomer showed markedly lower potency [95].

Continuing their studies, Paolino et al. expanded the series of 5-methoxy-indanone derivatives to investigate the effect of the amino-ammonium core on diastereoisomer-influenced activity and to develop pharmacological agents with high affinity and selectivity that can be reversibly modulated. The 2-benzylidenoindan-1-one derivatives were initially synthesized as E isomers, which exhibited good selectivity for AChE and MAO-B. Among these, compound **219** (Figure 38), containing a para *N*-benzyl(ethyl)amino substituent on the benzylidene ring, was the most potent, achieving an IC_50_ of 39 nM for AChE, approximately three times more active than analog **218**. UV-B exposure generated the *Z* isomers, yielding derivatives with reduced inhibitory potency [218].

More recently, the same group conducted further studies on the previously synthesized 2-benzylidenoindan-1-one derivative 219, revealing additional findings. The photochemical and photophysical properties of 219 were analyzed, showing that photoconversion between the active (E) and inactive (Z) forms occurs efficiently under physiological conditions (PBS, pH 7.4) and can be interconverted by UV (330 nm) or visible light (400 nm) irradiation. Moreover, the derivative demonstrated moderate solubility and the ability to cross the blood–brain barrier in PAMPA assays. Cytotoxicity studies in endothelial and neuronal cells indicated toxicity only at concentrations far exceeding those required for enzymatic inhibition, suggesting an adequate safety margin [219].

**Figure 39:**
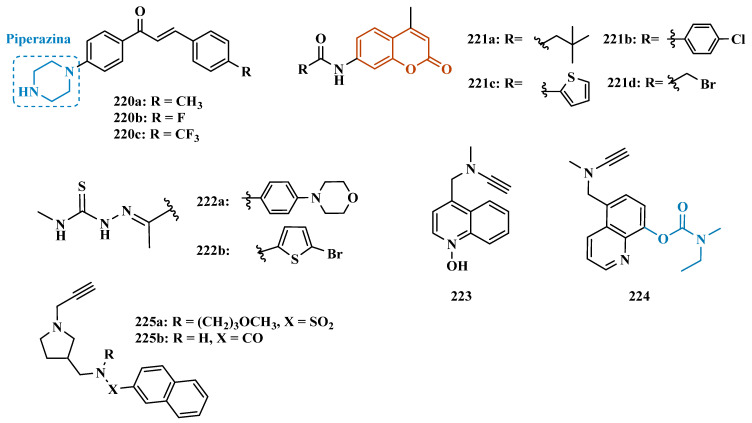
Chemical structures of piperazine-chalcone hybrids **220a**–**220c**, 7-amine-coumarin derivatives **221a**, **221b** with elective MAO-B inhibitory activity, and **221c** and **221d** with dual selective inhibitory activity of MAO-B/BACE-1 and MAO-B/AChE, respectively, aryl-thiosemicarbazones **221a** and **221b** with selective inhibitory activity of MAO-B and AChE, respectively, and N-propargylpyrrolidine derivatives **225a**, **225b**, with inhibitory activity of BuChE and MAO Structure of the iron chelator M30 (**222**) and proto-chelator derivative **223** with AChE inhibitory activity.

Considering that many synthetic chalcones have demonstrated selectivity for MAO-B inhibition, acting as competitive, selective and reversible inhibitors, and that the introduction of substituents on one or both nitrogen atoms of the piperazine system has led to dual MAO and AChE inhibitors, Mathew and co-workers designed a novel series of substituted piperazine-chalcone derivatives. These compounds were aimed at multifunctional inhibition of MAOs, ChEs and β-secretase (BACE-1), an enzyme responsible for the cleavage of the β-amyloid precursor protein. In general, all compounds tested in vitro exhibited selective inhibition of MAO-B and low inhibition of ChEs, except for compound **220a** (Figure 39). Particularly, compounds **220a** (IC_50_: MAO-A = 29.4 µM, MAO-B = 2.72 µM, AChE = 8.77 µM; BACE-1 = 15.5 µM), **220b** (IC_50_: MAO-A = 31.4 µM, MAO-B = 0.65 µM; AChE = 28 µM; BACE-1 = 14.9 µM;) and **220c** (IC_50_: MAO-A = 34.9 µM, MAO-B = 0.71 µM; AChE = 26.3 µM; BACE-1 = 15.3 µM) stood out for showing the best balanced multifunctional inhibitory profile on all four molecular targets of interest. Additionally, compounds **220b** and **220c** demonstrated to inhibit MAO-B in a selective, competitive and reversible manner, with good ability to cross the BBB and good passive gastrointestinal absorption [120].

Rodríguez-Enríquez and cols. explored the structure of substituted coumarins as potential multifunctional MAO, AChE, BuChE, BACE-1 inhibitors, as well as neuroprotective agents. Thus, they synthesized new 7-amide-coumarins, leading to compounds **221a** (Figure 39, IC_50_ = 0.25 μM) and **221b** (IC_50_ = 0.31 μM) as the most selective MAO-B inhibitors. On the other hand, compound **221c** (IC_50_: MAO-B = 1.59 μM; BACE-1 = 34.49 μM) stood out as the best dual inhibitor of MAO-B and BACE-1, while derivative **221d** (IC_50_: MAO-A = 78.16 μM; AChE = 3.78 μM) exhibited the best dual selectivity against MAO-A and AChE. Additionally, none of these compounds showed significant neurotoxicity in rat cortex motor neurons. Moreover, kinetic studies demonstrated that **221b** act as a reversible inhibitor, while compounds **221b**, **221c**, **221d** showed adequate predicted ADME properties, including BBB permeability [122].

The thiosemicarbazone functional group is a pharmacophore with structural characteristics that enhance MAO inhibition, such as the presence of a relatively acidic S = C-NH group, along with H-bond acceptor and donor sites. In addition, studies indicate that aryl-thiosemicarbazones typically exhibit increased MAO-B inhibition. Based on these findings, Mathew and co-workers synthesized new thiosemicarbazone derivatives to develop new dual MAO/AChE inhibitors. Biological screening led to the identification of compound **222a** (IC_50_ = 5.48 μM, SI > 7.30) as the most active and selective MAO-B inhibitor. This derivative also demonstrated to act as a competitive and reversible inhibitor, with no toxicity on Vero cells. Differently, compound **222b** (IC_50_ = 12.9 µM) stood out as the most potent AChE inhibitor, without significant effect on MAO activity [220].

Several previous studies conducted by Youdim et al. [221,222,223,224,225,226] reported the synthesis and pharmacological evaluation of a series of iron-chelators, and compound M30 (**223**, Figure 39) stood out for its promising and singular properties. This compound had been rationally designed by the combination of an iron-chelating hydroxyquinone fraction with a propargyl fragment, inspired by the structure of the selective MAO-B inhibitors rasagiline (**11**) and selegiline (**12**), approved as anti-Parkinson drugs [225]. In vitro studies revealed M30 as a potent MAO-A (IC_50 MAO-A_ = 37 nM) and MAO-B IC_50 MAO-B_ = 57 nM) inhibitor, aside from an iron-dependent inhibition of lipid peroxidation (IC_50_ = 9.22 μM), and antioxidant activity. In addition, compound M30 enhanced 85% of the cellular viability of PC12 cells, and was able to attenuate cell death induced by serum deprivation and 6-OHDA (at 0.1 μM) [226]. More recently, this compound was optimized, leading to a new series of multifunctional site-activated chelators with dual inhibition of AChE and MAO. As a result, the carbamoyl derivative **224** was identified as the best dual selective inhibitor of MAO-A (IC_50_ = 7.7 nM, showing a 1,026-fold higher selectivity for MAO-A (IC_50 MAO-B_ = 7.90 μM), and a time-dependent inhibition of AChE. In addition, compound **224** exhibited low affinity for metal ions such as Fe, Cu, and Zn until being activated by AChE, releasing the iron-active M30. Moreover, compounds **223** and **224** did not exhibit toxicity against neuroblastoma cells (SH-SY5Y) in low concentration, although derivative **224** has shown limited cytotoxicity at higher concentrations [227]. 

Butyrylcholinesterase (BuChE) is a serine hydrolase involved in various physiological functions, including the co-regulation of cholinergic neurotransmission, drug metabolism, and neuroinflammation. Although most BuChE inhibitors described in the literature have been designed for Alzheimer’s disease, these agents also show potential in other neurological disorders, such as Parkinson’s disease. In this context, in the search for inhibitors capable of simultaneously modulating BuChE and MAO isoforms, the Košak group synthesized and evaluated new N-propargylpyrrolidine-based analogs, demonstrating that the naphthalen-2-yl and N-propargyl moieties are essential for hBuChE and hMAO-B inhibition, respectively. The sulfonamide derivative **225a** (Figure 39) was the most potent hBuChE inhibitor (IC_50_ = 0.203 μM) yet exhibited weak MAO inhibition (IC_50_ > 100 μM). In contrast, the carboxamide analog **225b** (Figure 39) displayed MAO inhibitory activity, with IC_50_ values of 6.42 and 7.83 μM for MAO-A and MAO-B, respectively, and kinetic studies revealed that it acts as an irreversible inhibitor. Against BuChE, compound **225b** showed moderate activity (IC_50_ = 56.3 μM). Finally, in vitro evaluations indicated that compounds **225a** and **225b** are capable of crossing the blood–brain barrier in PAMPA assays, exhibit no cytotoxicity in SH-SY5Y and HepG2 cells, and reduce β-amyloid-induced neuronal cell death [228].

#### 3.2.3. Dual Adenosine Receptors Antagonists and MAO Inhibitors 

**Figure 40:**
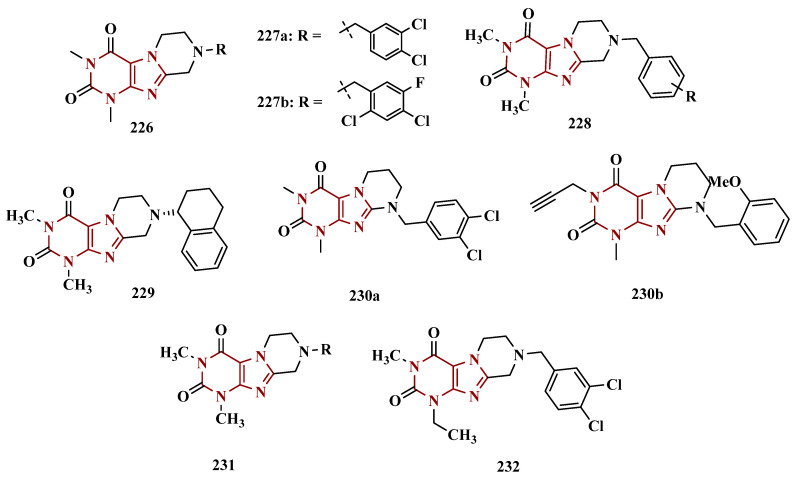
Chemical structures of compound **226**, used as a prototype for the design of its *N*-benzyl analogues **227a** and **227b**; xanthine derivative **228** and its derivative **229**; tetrahydropyrimido[2,1-f]purinodione derivatives **230a**, **230b**; and dimethylxanthine scaffold **231**, which led to the design of the multifunctional derivative **232**.

Adenosine A_2A_ receptors are mainly present in the striatum, and their antagonists have been shown to enhance neurotransmitter signaling via the DpA receptor. In contrast, the blockade of A_1_ receptor leads to increased DpA release and potentiates its effects in CNS [229]. Therefore, concomitant inhibition of MAO and adenosine receptors has been described as beneficial for both symptomatic treatment and neuroprotection [230]. Among the four adenosine receptor types (e.g., A_1_, A_2A_, A_2B,_ and A_3_), receptors A_1_ and A_2A_ predominate in the CNS. Thus, A_1_ antagonists can be used in treatments aimed at recovering cognitive deficits, while blocking A_2A_ receptors produces antiparkinsonian and neuroprotective effects [231,232]. In this context, xanthine derivatives have been described as adenosine receptor antagonists, and several research groups have explored its structure as a suitable scaffold for the development of novel adenosine antagonists with multifunctional properties aimed at the treatment of NDs, such as PD [230,233]. In previous studies, Brunschweiger and co-workers investigated a series of 8-benzyltetrahydropyrazino[2,1-f]purinedione derivatives, which exhibited antagonist activity for adenosine receptor and MAO inhibition. In another study, they identified certain tetrahydropyrimido[2,1-f]purinedione analogues as potent A_2A_ antagonists, while other analogues were selective for A_1_ antagonists [233,234,235,236,237]. However, all these active compounds showed poor water solubility, leading the authors to propose new optimized tetrahydropyrazino-purinedione derivatives based on the structure of the prototype **226** (Figure 40). As a result, they obtained a series of water-soluble derivatives at pH 1, with compounds **227a** (rat AR Ki A_1_ = 351 nM, A_2A_ = 322 nM; rat MAO-B: IC_50_ = 260 nM, Figure 40) and **227b** (human AR: Ki A_1_ = 217 nM, A_2A_ = 268 nM; human MAO-B: IC_50_ = 508 nM) standing out due to their nanomolar balanced multifunctional potency against A1/A2A receptors and MAO-B. Further pharmacokinetic studies demonstrated good oral bioavailability and favorable BBB permeability for compound **227a** [231]. In another study, still inspired by the xanthine scaffold, the same group had previously synthesized compound **228** (Figure 40), an *N*-benzyl tricyclic xanthine derivative that exhibited antagonist effects on A_1_/A_2A_ receptors and MAO-B inhibition [231]. Thus, in the search for new optimized xanthine-based dual adenosine receptor antagonists and MAO inhibitors, the authors identified compound **229** as a multi-target nanomolar antagonist of adenosine receptors (Ki A_1_ = 393 nM, A_2A_ = 595 nM) and selective MAO-B inhibitor (IC_50 MAO-B_ = 210 nM).

Koch and co-workers also explored the xanthine scaffold with the goal of discovering new neuroprotective agents, especially A_2_ antagonists. Taking into account that caffeine (**98**, Figure 16) may exhibit protective effects against PD and AD, the authors investigated a new series of tetrahydropyrimido[2,1-f]purinodiones, which were evaluated for their inhibitory activity against MAO-B and their potential interaction with the A_4_ adenosine receptor subtype. Among all tested compounds, the 3,4-dichlorobenzyl derivative **230a** (IC_50 MAO-B_ = 62.9 nM) stood out due to its highest selective MAO-B inhibition. In contrast, the *N*-propargyl derivative **230b** exhibited the best-balanced multi-target activity, blocking A_1_ and A_2_ receptors and inhibiting MAO-B (Ki: A_1_ = 0.605 µM, A_2A_ = 0.417 µM, IC_50 MAO-B_ = 1.80 µM) [232]. In a continuing effort addressed to new multi-target inhibitors of the MAO and adenosine receptors, Koch’s group synthesized a series of tetrahydropyrazino[2,1-f]purinedione derivatives. These compounds were designed based on the structure of dimethylxanthine **231** (Figure 40), which was modified by replacing the 1,3-dimethyl groups by other alkyl substituents. As a result, compound **232** was identified as the most effective dual nanomolar A_1_/A_2A_ antagonist (Ki: A_1_ = 396 nM; A_2A_ = 1620 nM) and MAO-B inhibitor (IC_50 MAO-B_ = 106 nM). Additionally, despite its lower potency, this compound also exhibited high affinity for the adenosine subtypes A_2B_, A_3_, as well as MAO-A inhibition [238].

**Figure 41:**
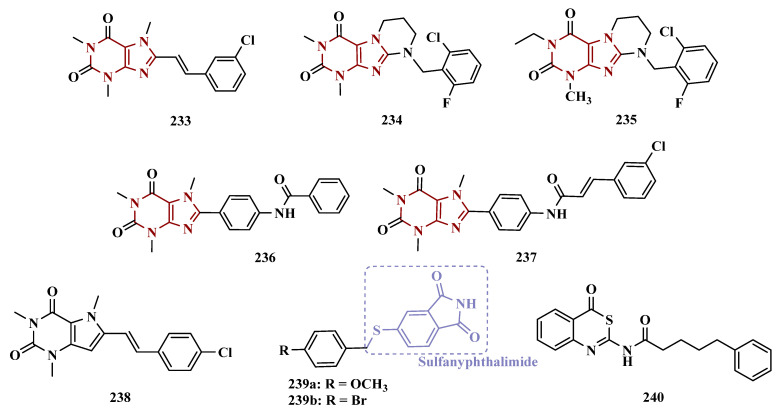
Chemical structures of 8-chlorostyrylcaffeine (**233**) and its tricyclic dimethyl-xanthine-based derivative **234**; 1-methyl-3-ethyl-xanthine derivative **235** and phenylamide-xanthine derivatives **236**–**237**; (*E*)-8-(3-chlorostyrene)-caffeine derivative **238**, sulfanylphthalimide derivatives **239a**, **239b** and the non-xanthine derivative **240** with dual inhibitory activity against MAO-B and antagonist activity on adenosine A_2A_ receptors.

Among the xanthine derivatives, 8-styrylxanthine group includes compounds such as istradefylline (**5**, Figure 2), an A_2A_ receptor antagonist used as an adjunct therapy for PD, and 8-chloro-styrylcaffeine (**233**, Figure 41), which also act as an A_2A_ antagonist and MAO-B inhibitor. In addition, tricyclic xanthine analogues, featuring a third ring connected to the f-bond of the 2,6-purinedione system and substituted by a benzyl group, have been considered as a bioisoster of (*E*)-8-styrylxanthine. Thus, Załuski et al. synthesized new fused-tricyclic xanthine derivatives with aromatic substituents at the tetrahydropyrimidine moiety (Figure 41), aiming at new dual MAO-B inhibitors and A_2A_ antagonists. Biological results revealed no significant activity against MAO-A, standing out compound **234** (Ki A_2A_AR = 189 nM; IC_50 MAO-B_ = 570 nM), which exhibited strong affinity for A_2A_ receptor and potent and selective inhibitory activity against MAO-B, with low hepatotoxicity (HepG2 cells) [233].

Following a similar strategy, Kuder et al. also explored the 1,3-dimethylxanthine scaffold to design new 1,3-dialkylxanthine derivatives as potential MAO-B inhibitors and adenosine receptor antagonists. As a result, the 1-methyl-3-ethyl-xanthine analogue **235** was identified as a promising nanomolar A_2A_ antagonist and a selective nanomolar MAO-B inhibitor (Ki_A2A_ = 264 nM, MAO-B IC_50_ = 243 nM); however, it exhibited poor predicted ADMET properties [230].

In another proposal, Wang et al. hypothesized that the phenyl-xanthine fragment could play a role in the A_2A_ antagonist effect of xanthine derivatives. Thus, they synthesized a series of phenyl-xanthines, leading to the identification of compounds **236** (Ki_A2A_ = 0.33 µM, IC_50 MAO-B_ = 0.29 µM) and **237** (Ki_A2A_ = 0.85 µM; IC_50 MAO-B_ = 0.63 µM) as the most potent dual A_2A_ antagonists and MAO-B inhibitors. These two compounds also demonstrated adequate in vivo ability to cross the BBB, with no relevant cytotoxicity on SH-SY5Y cells. Moreover, compound **236** was capable of reducing in vivo haloperidol-induced catalepsy in a dose-dependent manner, while analogue **237** exhibited similar effects only at higher doses [239].

In the search of optimized dual adenosine receptors antagonists and selective MAO-B inhibitors, Rivara and cols. proposed structural modifications on the structure of (*E*)-8-(3-chlorostyryl)-caffeine (**233**, Figure 41). Biological screening revealed compound **238** (Figure 41) as the most effective and selective MAO-B inhibitor (IC_50 MAO-A_ = 10 µM, MAO-B = 200 nM), showing a 50-fold higher selectivity for the MAO-B isoform. In addition, this compound was also capable to neutralize the haloperidol-induced catalepsy in vivo. However, regarding the antagonist effect on adenosine receptors, compound **238** exhibited low selectivity for A_2A_ (Ki = 260 nM), also blocking the A_1_ and A_3_ subtypes [240].

Recent studies have shown that 5-sulfanylphthalimides could act as potent MAO inhibitors. Thus, in a different approach, Walt et al. explored the phthalimide scaffold to design new 4- and 5-sulfanylphthalimide analogues as multifunctional ligands with potential neuroprotective properties against PD and AD. Biological evaluation focused on MAO inhibition and antagonist effect on adenosine receptors, highlighted compounds **239a** (Ki A_1_ = 0.369 µM) and **239b** (Ki A_1_ = 0.676 µM), as the most potent A_1_ antagonists. Notably, the bromobenzyl-sulfanyl derivative **239b** (IC_50 MAO-A_ = 0.273 µM; MAO-B = 0.0074 µM, SI = 36.9), which showed 1.8-fold lower affinity for A_1_ receptor than its methoxybenzyl analogue **239a**, also exhibited lower selectivity for MAO isoforms than **239a** (IC_50_: MAO-A = 1.63 µM, MAO-B = 0.020 µM), which showed a 81.5-fold higher selectivity for MAO-B [229].

In another study, searching for non-xanthine-based neuroprotective compounds, Stößel et al. investigated new 4*H*-3,1-benzothiazin-4-one derivatives designed to target both the A_2A_ adenosine receptor and MAO-B. Among them, compound **240** (Ki A_2A_ = 39.5 nM) exhibited the highest selectivity and nanomolar affinity for the A_2A_ receptor. Additionally, this compound demonstrated the greatest potency in the selective inhibition of MAO-B, acting in a reversible and competitive manner [241].

#### 3.2.4. Dual MAO and Catechol O-Methyltransferase Inhibitors

**Figure 42:**
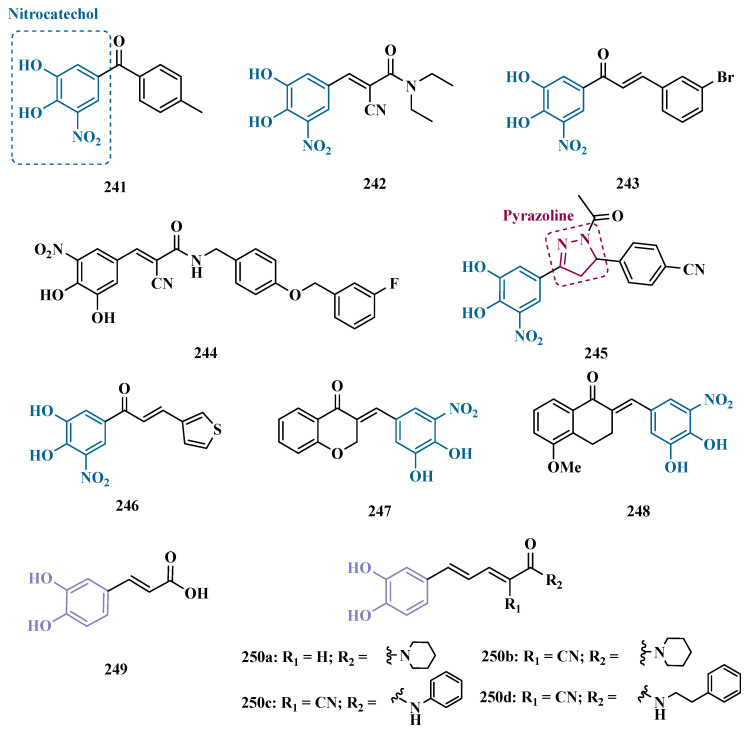
Chemical structures of the nitrocatecholic drugs tolcapone (**241**) and entacapone (**242**), the nitrocatechol-chalcone derivatives **243**–**248**, caffeic acid (**249**) and its homologues **250a**–**250d**.

The Nitrocatechol system is a pharmacophore subunit found in the structure of some FDA-approved catechol *O*-methyltransferase (COMT) inhibitors, such as tolcapone (**241**) and entacapone (**242**, Figure 42). This enzyme plays a key role in the metabolism of DpA and levodopa, and COMT inhibitors are commonly used as adjunctive treatments for PD. Thus, inspired by the structural features of chalcone and nitrocatechol, Engelbrecht and co-workers synthesized a series of nitrocatechol chalcones as potential dual inhibitors of MAO and COMT. In vitro biological studies identified the bromoaryl derivative **243** as the most well-balanced dual inhibitor of both target enzymes, with IC_50_ values of 13.9 µM for MAO-B, and 0.29 µM for COMT. Additionally, this compound exhibited a reversible and competitive inhibitory mode against MAO-B [242].

Considering the involvement of COMT and MAO in dopamine metabolism, Zou et al. developed a series of novel dual inhibitors inspired by the nitrocatechol scaffold, aiming to identify multitarget candidates for Parkinson’s disease treatment. Among the synthesized compounds, derivative **244** (Figure 42) proved promising, with IC_50_ values of 0.37 μM and 15.98 μM for COMT and MAO-B, respectively. In vitro assays showed that the lead compound was non-cytotoxic and exerted protective effects against MPP^+^-induced damage in SH-SY5Y cells. Furthermore, in vivo pharmacological studies demonstrated that **244** is blood–brain barrier permeable and that oral administration increased dopamine levels and improved motor performance in MPTP-induced PD mice [243].

In a similar approach, Hitge et al. investigated another series of nitrocatechol-chalcones and their pyrazoline analogues. Among all tested compounds, the nitrocatechol-pyrazoline derivative **245** (Figure 42) exhibited the most effective inhibition of COMT (IC_50_ = 0.048 µM), while the thiophene-nitrocatechol derivative **246** (Figure 42) stood out due to its best well-balanced inhibition of MAO and COMT (IC_50 MAO-A_ = 41.4 µM; MAO-B = 42.1 µM, COMT = 0.23 µM), but with no MAO-A/B selectivity [94]. Following the same hypothesis, Beer and co-workers also synthesized nitrocatechol-chalcones. Biological evaluation revealed that both 4-chromanone derivative **247** (Figure 42) and its methoxylated analogue **248** act as dual COMT (IC_50_ = 0.57 µM, 0.42 μM, respectively) and MAO-B inhibitors (IC_50_ = 7.26 μM, 7.83 μM, respectively), exhibiting similar inhibitory potencies for both target enzymes. In silico studies demonstrated that neither compound can cross BBB adequately, despite their good predicted gastrointestinal absorption, suggesting that they could be suitable for peripheral inhibition of COMT, but would not be effective MAO-B inhibitors in vivo [244].

The structure of caffeic acid (**249**, Figure 42), a natural phenolic compound with remarkable antioxidant properties, was explored by Chavarria and co-workers. They designed a series of structurally modified caffeic acid-based derivatives as potential MAO and COMT inhibitors. Biological evaluation revealed that none of the compounds was effective against MAO-A. However, compounds **250a** (IC_50_: MAO-B = 2.50 µM; COMT = 1.65 µM), **250b** (IC_50_: MAO-B = 4.27 µM; COMT = 1.33 µM), **250c** (IC_50_: MAO-B = 4.55 µM; COMT = 2.41 µM) and **250d** (IC_50_: MAO-B = 4.38 µM; COMT = 1.27 µM) stood out due to their balanced low-micromolar potencies as dual inhibitors of MAO-B and COMT. Moreover, these compounds showed the ability to protect neuronal cells against oxidative damage in the ORAC-FL assay, with no significant cytotoxicity in SH-SY5Y cells at 10 µM. Particularly, compounds **250a** and **250b** exhibited adequate BBB permeability by passive diffusion in the PAMPA assay [245].

#### 3.2.5. Caspase and MAO-Inhibitors

**Figure 43:**
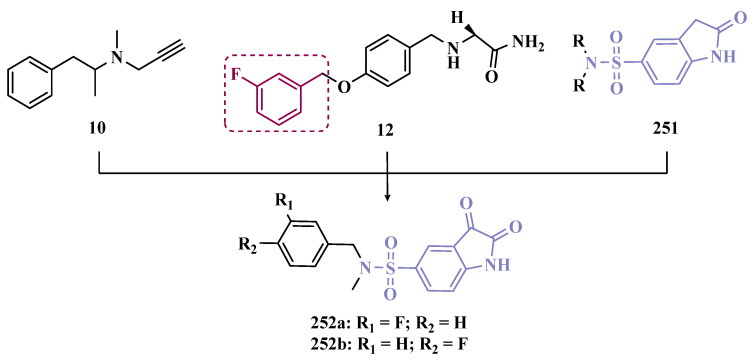
Representation of the rational design of a new series of hybrid isatin-*N*-disubstituted sulphonamides, based on the structures of selegiline (**10**), safinamide (**12**), and isatin sulfonamide **251**, resulting in the multifunctional MAO-A/B and caspase-3 inhibitors **252a** and **252b**.

Caspases are intracellular enzymes activated during the process of cell death. In particular, caspases-3 plays a crucial role in several apoptotic-related pathogenesis, including neurodegenerative diseases. Thus, Tavari and cols. explored the structural features of selegiline (**10**) and safinamide (**12**), two approved MAO inhibitors, as well as isatin sulfonamide **251** (Figure 43), a selective caspases-3 inhibitor, to design a new series of isatin-*N*-disubstituted sulphonamides. In vitro evaluation for their multifunctional inhibitory properties against MAO-A, MAO-B and caspase-3 led to identification of compounds **252a** (IC_50_: MAO-A = 22.75 µM; MAO-B = 8.32 µM; caspase-3 = 25.08 µM) and **252b** (IC_50_: MAO-A = 8.26 µM; MAO-B = 5.96 µM; caspase-3 = 29.26 µM) as promising dual MAO/caspase-3 inhibitors. In particular, compound **252a** exhibited 2.7-fold greater selectivity for MAO-B, and an almost equipotent inhibition of caspase-3 compared to **252b**. Additionally, both compounds were shown to act as reversible inhibitors with good predicted BBB permeability. Structure-activity analysis suggested that the fluorobenzylamine moiety plays a crucial role in the multitarget action, while propargylamine fragment appears to contribute only to increased MAO-A inhibition [246].

Taken together, in this section we highlighted the rational design of multifunctional and multitarget-directed compounds capable of inhibiting both MAOs in addition to concomitant modulation of other molecular targets, including cholinesterases, COMT, caspases, and adenosine and histamine receptors, aiming at neuroprotection in neurodegenerative diseases. Semicarbazone derivatives, hydrazone-based analogues, and chalcone-inspired molecules stood out for their balanced dual or multitarget profiles, with some compounds showing nanomolar potency, selectivity for MAO-B and other dual or multiple targets, in addition to antioxidant properties, and good predicted BBB permeability. These findings highlight promising scaffolds for developing multifunctional agents in neurodegenerative pathologies therapy, particularly for Parkinson’s disease.

## 4. Discussion

Among all synthetic MAO inhibitors analyzed in this brief review, resulting from the efforts of medicinal chemists from 2010 to 2025, some exhibited promising pharmacological properties to the development of novel selective MAO-B. Additionally, a smaller group of compounds demonstrated interesting dual or multi-target-directed pharmacological profile, positioning them as innovative neuroprotective agents against neurodegenerative diseases, particularly PD.

For a more objective analysis focused on identifying the most potent MAO-B inhibitors with better druggability profile, we conducted a comparative evaluation, including SI values and some selected predicted key-ADME parameters, of the most relevant compounds exhibiting selective MAO-B inhibition, as presented in Table 2. The full detailed table with all MAO-B inhibitors is available in the Supplementary Material.

Among the indole and indazole classes, Tzevetkov’s group stood out due to the development of compounds with high selectivity to MAO-B isoform. Among them, the indazole derivatives **16a**–**c**, **18** e **19a** exhibited the highest SI, evidencing their potential for the development of new MAO-B drug candidates. This series shows a disubstituted phenyl moiety (e.g., Cl and F) linked to a heterocyclic nucleus by a carboxamide (**16a**–**c e 19a**) or an imine functionality (**18**). These features were shown to play a relevant role in the potency and the affinity with the target-enzyme active site, favoring hydrophobic π-π interactions [55,56]. The presence of dichloro-substituents at the *meta*- and *para*-positions of the phenyl ring on compounds **16a**, **16b**, and **18** seems to contribute to enhanced hydrophobic interactions with the enzyme, resulting in SI values of 17,064, 25,906, and 16,339, respectively. In contrast, the introduction of other electron donor substituents (e.g., OMe and OH) at the same positions resulted in a lower MAO-B inhibition. Regarding compounds **16a** and **16b**, their difference is related to the *N*-substituent of the indazole ring, which is a hydrogen atom for **16a**, and a methyl group for **16b**. This slight difference seems to be sufficient to change the lipophilicity and the affinity for the target, as observed for the most lipophilic compound **16a** (LogP = 3.60), suggesting its higher probability to permeate lipid membranes than compound **16b** (LogP = 2.67). Additionally, methylation at the N-1 position of the indazole system in **16b** resulted in a slight increase in potency [55]. On the other hand, the 3,4-difluorophenyl substituent in compound **16c** and the 3-chloro-4-fluorophenyl group in **19a** enhanced the electrophilic character, influencing their potential interactions in the biophase and their bioavailability. Interestingly, derivative **16c** exhibited a higher LogP value (LogP = 3.72) when compared to the other analogues. However, despite this, it showed lower, albeit still high, selectivity for MAO-B (SI = 6,289). In contrast, derivative **19a**, which features a methyl group at the N-1 position of the indazole system, displayed significantly higher selectivity for MAO-B (SI = 15,105) [55,56].

Compounds **44a** and **48b** stood out as substituted hydrazones, a chemical class extensively studied for its potential as selective MAO-B inhibitors. Compound **44a**, exhibited an IC_50_ value of 4.4 nM and an SI of 19,977, making it one of the most selective and potent MAO-B inhibitors reported to date. Its structure features the strategic introduction of a methylene spacer, which promotes a more efficient fit into the enzyme’s active site, directly contributing to its high affinity and selectivity [70]. Conversely, compound **48b**, part of the 4-(3-nitrophenyl)thiazole-2-yl-hydrazone series, also displayed high inhibitory activity and selectivity for MAO-B. In this case, the thiophene nucleus and a NO_2_ group on the aromatic ring play a crucial role in the enzyme interaction [74]. A comparison between these compounds highlights that substituted hydrazones, particularly those containing electronegative groups and heterocyclic nuclei, represent a promising strategy for developing new selective MAO-B inhibitors.

Among the series of indanone derivatives, compounds **70**, **73c** and **73d** exhibited the highest selective indices (SI), although these values were lower compared to the other chemical classes and diverse structural scaffolds. Compound **70** contains a functionalized indanone core, which has been shown to enhance affinity for a specific biological target, resulting in a good selectivity index (SI = 980), as well as a reversible and competitive mode of action. Additionally, high-potency inhibitors were identified among *meta*- and *para*-substituted compounds bearing halogens in the benzyloxy ring [88]. Notably, compounds **73c** and **73d** are modified derivatives of rasagiline, an irreversible MAO-B inhibitor, and share a propargylamine nucleus. Substitution of the phenyl ring proved advantageous in enhancing MAO-B inhibitory activity. The presence of small substituents, such as hydroxyl in **73c** and acetoxy in **73d**, contributes to hydrophobic interactions within the active site, strengthening these interactions and increasing selectivity (SI = 934 and SI = 909, respectively) [91]

The chalcone scaffold also contributed to the discovery of promising selective MAO-B inhibitors. Among all accessed studies with this class of compounds, **82b** and **85a** exhibited the highest potency and selective indices, acting as reversible inhibitors of MAO-B. Compound **82b** features a hydroxy-substituted chalcone nucleus, which conferred the ability of more effective H-bond interactions and, in turn, improved selectivity (SI = 1,354) and affinity [102]. On the other hand, compound **85a** is a cinnamic acid derivative, featuring one bromine atom as substituent in each phenyl ring. This study revealed that a bromine atom as a substituent in the *para*-position led to a higher contribution to the enhanced MAO-B inhibition compared to Cl, F, or H. It was suggested that the presence of a *para*-Br as substituent in the phenyl ring is relevant to hydrophobic interactions within the enzyme active site, resulting in enhanced selectivity (SI = 9,387) [105].

The phtalonitrile derivative **96a** was identified as another particularly promising selective MAO-B inhibitor. This *para*-Br-substituted compound exhibited optimized properties for interaction with the target enzyme, demonstrating high selectivity (SI = 8,720) for MAO-B [112].

Among the alkaloid-based compounds investigated, the piperine-derived ligand **105** stood out for its good selectivity, as well as its reversible, competitive inhibition and good BBB permeability. However, its selectivity was lower compared to other chemical classes. SAR analysis revealed that the presence of an α-carbonyl-nitrile substituent and an α,β,γ,δ-unsaturated ketone linked to the benzyl ester moiety enhance both its selectivity and inhibitory activity against MAO-B [119].

The coumarin derivatives **112a**–**c** stood out for their high selectivity toward MAO-B. The series of 3-aryl-coumarins demonstrated that both the nature and position of substituents in the 3-phenyl ring significantly influence biological activity. For instance, the *para*-methyl-substituted derivative **112a** exhibited the highest potency (IC_50_ = 0.31 nM) and selectivity for MAO-B (SI > 333,300), outperforming its analogues substituted with methoxy and hydroxy groups at the same position. Conversely, in the *meta*-methoxy substituted analogue **112b**, a notable reduction in both potency (IC_50_ = 0.80 nM) and selectivity was observed, suggesting that weaker electron-donating groups may favor enzyme interaction. Notably, in compound **112c**, the combination of a halogen (Br) in the *para*-position with a methoxy group in the meta-position of the 3-aryl-coumarin scaffold improved both affinity and selectivity compared to **112b**, although it remained less potent and selective than **112a**. These findings reinforce the idea that an electron-donating group in the *para*-position, combined with a small lipophilic and electron-withdrawing substituent in the *meta*-position, can partially restore selectivity, albeit with a slight enhance in potency [127]. Compound **119** is another coumarin-based derivative distinguished by its remarkably high selectivity for MAO-B. The series of 3,4-dihydrocoumarins demonstrated that smaller substituents in the 7-benzyloxy moiety are more suitable for fitting and interacting with the MAO-B binding pocket. Additionally, the eletronic nature and position of substituents play a crucial role in modulating both potency and selectivity. For instance, the presence of a fluorine atom in the *ortho*-position of **119** significantly enhanced both inhibitory potency and selectivity (SI > 270,270) compared to its *meta*- or *para*-substituted analogues. Conversely, the introduction of larger and stronger electron-withdrawing substituents, such as Br and NO_2_, in the same position resulted in the reduction of MAO-B inhibition. These findings highlight that the F atom, a small and highly electronegative substituent, is ideal for optimizing volume adjustment within the binding site and improving enzyme interaction [125]. 

When further exploring benzopyrones, other compounds that demonstrated great potential for MAO-B inhibition were compounds **125** and **138a**,**b**. Compound **125**, a disubstituted chromone derivative, showed that small and appropriately positioned substituents, particularly in the *meta*-position, have the ideal profile for interactions within the enzyme’s active site, leading to high selectivity (SI > 149,254) and maximum activity [132]. On the other hand, compounds **138a** and **138b** are quinolinone derivatives with halogenated substituents in the *para*-position of the aromatic ring. Based on a SAR analysis, the study highlighted that halogenated substituents are essential for the biological activity of these compounds, as they enhance hydrophobic interactions within the enzyme’s active site and influence the dipole moment of the molecules. It was also demonstrated that halogen size and polarizability impact both potency and selectivity. Compound **138b**, substituted with a Br atom, exhibited higher affinity and inhibitory potency against MAO-B (SI > 40,000) compared to its chlorine-substituted analogue **138a** (SI = 20,643), as bromine is larger and more polarizable than chlorine [148].

The benziloxy-based derivatives **151**, **152**, **158**, and **159a**–**c** contain a substituted benzoxyl subunit in the phenyl-benzyloxy system, which serves as the key factor for their high inhibitory activity and selectivity toward MAO-B. In compound **151**, a CF_3_ group is present as a substituent, while in compound **152**, the substituent is a fluorine atom. The introduction of these substituents in the *para*-position contributes to hydrophobic interactions within the active site of MAO-B, leading to good inhibitory activity [157,158]. In compound **158** (SI > 25,641), a CF_3_ group was introduced in the meta-position of the benzyloxy ring, along with a chlorine atom at the *meta*-position of the phenylamide moiety, which was shown to increase the compound’s binding stability with the target enzyme [47]. Finally, derivatives **159a**–**c** exhibited a gradual increase in activity in the order **159c** < **159b** < **159a**. Despite their similar low-nanomolar potencies, the two *meta*-halogenated benzyloxy derivatives **159a** (F) and **159b** (Cl) exhibited higher inhibitory potency and selectivity (SI = 17,482 and SI = 13,615, respectively) compared to bulkier substitutions, such as the phenyl substituent in **159c** (SI = 9550) [163]. Thus, halogens and smaller substituents are essential for the high activity and selectivity observed in benzoxyl derivatives.

Regarding azoles, compounds **169** (SI = 17,111) and **180** (SI > 8354) stood out due to their high potency and good selectivity. The oxadiazole derivative **169**, features a sulfonamide substitution at the 4-position of the phenyl ring, which is crucial for more effective enzyme inhibition compared to derivatives substituted at the 3-position. Furthermore, both compounds contain two ortho-chlorine atoms as substituents in the phenyl ring, significantly enhancing their affinity for MAO-B by strengthening hydrophobic interactions within the enzyme’s active site. Notably, the isoxazole derivative **180** exhibited both lower potency and selectivity compared to **169**, suggesting a possible auxophoric contribution of the central heterocyclic system to biological activity [169,187].

Among the molecular hybrids designed as selective MAO inhibitors, compounds **192** (SI = 1,666.67), **19a** (S > 363), and **200** (SI = 475) exhibited the highest MAO-B selectivity. Compound **192**, with a coumarin-pyridazine hybrid architecture, demonstrated the highest potency in its series. The presence of a Br atom at the C7 position of the coumarin core conferred greater activity and selectivity compared to derivatives substituted with a Cl atom at C6 or C8 or a Br atom at the C8 position [198]. Compound **195a** was structurally designed by incorporating an aromatic and a heteroaromatic core connected by an enamide bond, inspired by the structures of lazabemide (**26**), safinamide (**12**), and chalcone (**74**). The chlorine atom at the *para*-position of the arylamide moiety provided the highest inhibitory potency, followed by CH_3_, H, Br, and F, respectively [202]. Meanwhile, compound **200** was rationally designed through molecular hybridization of procaine and imidazole cores. The *para*-hydroxyl group in the phenolic ring stabilizes H-bond interactions, while the F atom enhances affinity for hydrophobic targets. The combination of these features resulted in remarkable potency and the highest selectivity among its analogues [204].

Given the multifactorial nature of PD, multitarget approaches have emerged as a promising strategy for the design of new inhibitors directed at multiple targets. Among the dual inhibitors targeting MAO-B and AChE, compounds **213b** and **213c** stood out. The molecular structures of these inhibitors were rationally designed through molecular modeling, aiming for inhibitory activity against MAO and ChEs, which led to the identification of 4-(3-nitrophenyl)thiazol-2-yl hydrazones as a key scaffold. Molecular docking analyses and scoring functions revealed that the interaction between the nitrogen atom of the triazole ring and Cys172 in MAO-B is responsible for the isoform selectivity of these compounds. In MAO-A, the corresponding residue at this position is Asn181, which prevents this interaction. Furthermore, the target interaction energies higher than 0 kcal/mol for compound **213c** suggest that the hydrazone moiety introduces an unfavorable steric hindrance in MAO-A, further supporting the selectivity for the desired isoform. Finally, the addition of a cyclopropylethylidene group in **213b** and a cyclohexylethylidene group in **213c** contributed to the remarkable MAO-B inhibition, exhibiting IC_50_ values of 53 nM and 72 nM, respectively, as well as effective AChE inhibition. [216].

Among the derivatives evaluated as potential inhibitors of MAO and adenosine receptors, compound **240** exhibited promising properties. The introduction of a 4-phenylbutyryl substituent in this derivative resulted in an extended spacer, which contributed positively to its affinity for the human A_2_A receptor. In contrast, other inhibitors in the series demonstrated that the addition of a 3-methoxy group, as well as halogen substitutions (F, Cl, and Br) on the phenyl ring, reduced adenosine receptor affinity or led to a complete loss of binding, as observed in disubstituted compounds. A similar trend was observed for MAO-B inhibition, where compound **240** showed remarkable inhibitory activity (IC_50_ = 34.9 nM), while phenyl ring substitution significantly decreased affinity [242]. 

## 5. Conclusions

Monoamine oxidases are enzymes located in the outer membrane of mitochondria and exist in two isoforms, MAO-A and MAO-B, with the latter being predominantly expressed in the CNS. These enzymes have been implicated in the pathophysiology of PD, as their excessive enzymatic activity promotes the formation of ROS, hydrogen peroxide, ammonia, and aldehydes, which, in turn, contribute to dopaminergic neuronal death and progression of PD. The search for MAO inhibitors has grown significantly in recent years, and MAOs have been recognized as important targets for the development of disease-modifying drugs against PD. Over the past decade, Medicinal Chemistry has achieved remarkable progress in the development of novel MAO inhibitors, especially MAO-B, which are selective compounds with nanomolar potency, neuroprotective properties, and favorable pharmacokinetic profiles. A key example of this strategy is safinamide, a recently approved drug for PD treatment that acts as a selective and reversible MAO-B inhibitor, offering fewer side effects compared to non-selective and irreversible inhibitors. In this context, numerous research groups, primarily from academia, have dedicated significant efforts to the design, evaluation, and optimization of new synthetic chemical entities, aiming to discover novel chemotherapeutic alternatives with enhanced pharmacological effectiveness and drug-like properties. The increasing number of publications on this topic highlights its growing relevance in medicinal chemistry, contributing to a significant expansion of chemical space and structural diversity while also yielding many promising ligands for drug development. These advances highlight their therapeutic potential as monotherapies in early-stage Parkinson’s disease and as adjuvants to levodopa in advanced cases. Nevertheless, most findings remain confined to preclinical studies, and unresolved issues such as long-term safety, off-target effects, and clinical translation still pose significant challenges. Importantly, the emergence of multifunctional agents, combining MAO inhibition with antioxidant, anti-inflammatory, or cholinesterase-blocking activities, represents a particularly promising avenue to address the multifactorial nature of Parkinson’s pathology. Among the various strategies of the rational design of new scaffolds and optimized drug candidate prototypes, several ligands have been identified as selective MAO inhibitors. Additionally, numerous studies have focused on the search of dual, multifunctional or multi-target-directed ligands, leading to the discovery of bioactive compounds that exhibit other pharmacological properties beyond MAO inhibition. These compounds have demonstrated the ability to modulate cholinesterase activity, histamine and adenosine receptors, catechol-*O*-methyltransferase (COMT), and caspase, among other molecular targets. Therefore, the ongoing search for innovative drug candidates, aimed not only at exploring novel structural architectures but also at identifying new mechanisms of action, remains crucial for advancing PD therapeutics and medicinal chemistry as a whole. This continuous effort could lead to the development of potential new drugs with fewer side effects and greater therapeutic effectiveness for neurodegenerative diseases, such as PD.

Future efforts should prioritize the optimization of drug-like properties, including CNS permeability and metabolic stability, alongside comprehensive in vivo validation. Greater emphasis on translational studies and clinical trials will be essential to bridge the gap between preclinical promise and therapeutic reality. Ultimately, the design of multifunctional and highly selective MAO-B inhibitors could pave the way for safer, more effective, and disease-modifying therapies for Parkinson’s disease.

## Figures and Tables

**Table 2:** Evaluation of the druggability profile of the most potent and selective MAO-B inhibitors.

Inhibitor	IC_50_ MAO-A (nM)	IC_50_ MAO-B (nM)	SI	Inhibitory Profile	LogP	TPSA	BBB Penetration	Toxicity
**16a**	>10,000	0.586 ± 0.087	17,064	Rev/Comp	3.60	57.8	-	-
**16b**	>10,000	0.386 ± 0.052	25,906	-	2.67	57.8	-	-
**16c**	>10,000	1.59 ± 0.16	6289	-	3.72	46.9	-	-
**18**	>10,000	0.612 ± 0.065	16,339	-	4.38	41	-	-
**19a**	>10,000	0.662 ± 0.059	15,105	-	-	46.92	Yes	-
**44a**	87,900 ± 4780	4.4 ± 0.2	19,977	-	-	-	-	-
**48b**	6470 ± 1250	2.5 ± 0.15	2588	-	-	-	-	-
**70**	3920 ± 827	4.0 ± 1	980	Rev/Comp	-	-	-	-
**73c**	25,2200 ± 20,400	270 ± 20	934	-	-	-	-	-
**73d**	436,500 ± 40,300	480 ± 4	909	-	-	-	-	-
**82b**	15,370	11.35	1354	Rev	-	-	-	-
**85a**	5820 ± 720	6.2 ± 0.9	938.7	Rev/Comp	-	-	-	-
**96a**	218,000	25	8720	Rev	-	-	-	-
**105**	-	47.4	>211	Rev/Comp	-	68.55	-	Low
**112a**	NA	0.31 ± 0.02	>333,333	-	-	-	-	-
**112b**	NA	0.80 ± 0.05	>125,000	-	-	-	-	-
**112c**	NA	0.74 ± 0.02	>135,870	-	-	-	-	-
**119**	99,999 ± 0.53	0.37 ± 40	>270,270	Rev/Comp	-	35.53	Yes	Low
**125**	NA	0.67 ± 0.13	>149,254	Rev/Comp	3.69	59.31	Yes	Low
**138a**	28,900 ± 4220	1.4 ± 0.3	20,643	-	-	-	-	-
**138b**	>100,000	2.5 ± 0.7	>40,000	-	-	-	-	-
**151**	>100,000	9 ± 1	110,000	Comp	-	-	-	-
**152**	>100,000	12.34 ± 1.62	>8104	-	3.66	35.53	Yes	Low
**158**	-	3.9 ± 0.7	>25,641	-	-	-	-	-
**159a**	50,700 ± 4450	2.9 ± 0.3	17,482	-	-	-	-	-
**159b**	17,700 ± 2940	1.3 ± 0.3	13,615	-	-	-	-	-
**159c**	38,200 ± 3130	4 ± 1	9550	-	-	-	-	-
**169**	46,200 ± 11,200	2.7 ± 0.64	17,111	Rev	-	-	-	-
**180**	NA	11.97 ± 0.37	>8354	-	-	-	-	-
**192**	>100,000	60 ± 4	1666.67	Rev	-	-	Yes	-
**195a**	>40,000	110 ± 24	>363	Rev/Comp	-	-	-	-
**200**	15,220 ± 3400	32 ± 2	475	-	-	-	-	Low
**213b**	2660 ± 51	5.3 ± 0.8	501	Rev/Comp	-	-	Yes	-
**213c**	29,100 ± 2520	7.2 ± 1.8	4041	-	-	-	Yes	-
**240**	>10,000	34.9 ± 2.5	286	Rev/Comp	-	-	-	-

**IC_50_ MAO-A/MAO-B:** ‘NA’ = not active compounds. **SI:** Selectivity index calculated from the ratio IC_50 MAO-A_/IC_50 MAO-B_. **Inhibition profile**: **Rev** = Reversible, **Comp** = Competitive. **TPSA (Â^2^):** topological polar superficial area; Values < 140 Â^2^ indicate good oral absorption; values < 90 Â^2^ suggest high probability of penetrating BBB. **LogP**: Lipophylic partition coefficient; ideal values are 0–3 log mol/L. **BBB penetration**: Indicates the ability to cross BBB (Yes or Not). **Toxicity**: Classified as low, moderate, or high. - indicates data not shown in the original paper.

## Data Availability

Not applicable.

## Supplementary Materials

The following supporting information can be downloaded at https://www.mdpi.com/article/10.3390/ph18101526/s1.

## Abbreviations

The following abbreviations are used in this manuscript:

PD	Parkinson’s disease
AD	Alzheimer’s disease
ATP	Adenosine triphosphate
ATP synthase	Adenosine triphosphate synthase
BBB	Blood–brain barrier
CI	Complex I
CIII	Complex III
LB	Lewy’s bodies
COMT	Catechol *O*-methyltransferase
DpA	Dopamine
ND	Neurodegenerative disease
DNA	Deoxyribonucleic acid
DOPAC	3,4-dihydroxyphenylacetic acid
DOPAL	3,4-dihydroxyphenylacetaldehyde
FAD	Flavine-adenine dinucleotide
ROS	Reactive oxygen species
OS	Oxidative stress
RNS	Reactive nitrogen species
GSH	Glutathione
LAS	Lysosomal autophagic system
LDH	Lactate dehydrogenase
CSF	Cerebrospinal fluid
MAO	Monoamine oxidase
MnSOD	Manganese superoxide dismutase
MPTP	1-methyl-4-phenyl-1,2,3,6-tetrahydropyridine
NM	Neuromelanin
NMDA	*N*-methyl-*D*-aspartate
NO	Nitric oxide
PTP	Permeability transition pore
SN	Substantia nigra
CNS	Central nervous system
SAR	Structure-activity relationship
GABA	Gamma-aminobutyric
AChEs	Acetylcholinesterases
α-SYN	α-synuclein
