# Supplementary material for: Monoamine Oxidase Inhibitors in Drug Discovery Against Parkinson’s Disease: An Update
Source: Pharmaceuticals (Basel). 2025 Oct 10;18(10):1526. doi: 10.3390/ph18101526 (PMC12567508; doi:10.3390/ph18101526)
Supplement: Supplementary file 1 [file pharmaceuticals-18-01526-s001.zip › pharmaceuticals-3857755-supplementary.pdf]

Review

Monoamine Oxidase Inhibitors in Drug Discovery Against Parkinson's Disease: An Update

Luana Vergueiro Ribeiro [1], Larissa Emika Massuda [1], Vanessa Silva Gontijo [1], and Claudio Viegas Jr. [1,*]

[1] PeQuiM – Laboratory of Research in Medicinal Chemistry, Institute of Chemistry, Federal University of Alfenas, Alfenas-MG, 37133-840, Brazil.

Table S1 - Evaluation of the Druggability profile of Indazole and Indole/Melatonin-like Inhibitors.

Indazole and Indole/Melatonin-*like* Inhibitors

Inhibitor	Selectivity	IC_{50} MAO-A (nM)	IC_{50} MAO-B (nM)	SI	Inhibitory profile	Toxicity	Molecular weith	TPSA	LogP	BBB penetration	Ki MAO-B (nM)	Ki MAO-A (nM)
**14a**	MAO-B	>100,000	1,410	>71	-	Low	270.71	44.89	-	Yes	-	-
**14b**	MAO-B	>100,000	910	>109	-	Low	315.16	44.89	-	Yes	-	-
**15**												
**16a**	MAO-B	>10,000	0.586 ± 0.087	17,065	R/C	-	306	57.8	3.6	-	-	-
**16b**	MAO-B	>10,000	0.386 ± 0.052	25,906	-	-	320	57.8	2.67	-	-	-
**16c**	MAO-B	>10,000	1.59 ± 0.16	6,289	-	-	273	46.9	3.72	-	-	-
**17**	MAO-B	1,300 ± 68	0.227 ± 0.039	5,727	-	-	305	44.9	4.37	-	-	-
**18**	MAO-B	>10,000	0.612 ± 0.065	16,340	-	-	290	41	4.38	-	-	-
**19a**	MAO-B	>10,000	0.662 ± 0.056	15,106		-	303.72	46.92	-	Yes	-	-
**19b**	MAO-B	562 ± 62	8.08 ± 1.05	70		-	303.72	46.92	-	Yes	-	-
**20a**	MAO-B	>10,000	1.111 ± 0.16	>9,009	R/C	-	306	57.8	-	Yes	0.50 ± 0.07	-
**20b**	MAO-B	>10,000	3.27 ± 0.04	>3,058	R/C	-	289	57.8	-	Yes	1.45 ± 0.02	-
**23a**	MAO-B	>100,000	1,650	>60	-	-	-	-	-	-	-	-
**23b**	MAO-B	>100,000	780	>120	R/C	-	-	-	-	-	-	-
**24a**	NS	4 ± 1	20 ± 6	0,2	-	-	-	-	-	-	-	-
**24b**	NS	14 ± 1	17 ± 3	0,8	R/C	-	-	-	-	-	-	-
**27**	MAO-A	147 ± 33	>100,000	>680	-	-	-	-	-	-	-	-
**28a**	MAO-A	250 ± 99	>100,000	400	-	-	-	-	-	-	-	-
**28b**	MAO-B	1,750 ± 106	581 ± 66	3	-	-	-	-	-	-	-	-
**29**	NS	23 ± 8	178 ± 26	0.1	-	-	-	-	-	-	-	-

**30**	NS	813 ± 8	532 ± 37	2	-	-	-	-	-	-	-	-
**31a**	MAO-A	6 ± 0,5	196 ± 44	33	-	-	-	-	-	-	-	-
**31b**	MAO-B	20 ± 3	58 ± 13	3	-	-	-	-	-	-	-	-
**32**	MAO-B	>10,000	28 ± 1	>357	R	-	-	-	-	-	-	-
**33a**	MAO-B	>100,000	65 ± 6	>1,538	R/C	Low	-	-	-	-	24.74 ± 2	-
**33b**	MAO-B	>100,000	62 ± 6	>1,613	R/C	Low	-	-	-	-	33.74 ± 1.73	-
**33c**	MAO-B	>100,000	130 ± 8	>769	R/C	Low	-	-	-	-	76.50 ± 3.67	-
**35**	MAO-B	>100,000	42.1	>2,375	R/C	Low	305.16	44.89	4.06	Yes	7.335	-
**36**	MAO-B	>100,000	327.8	>305	-	-	-	-	-	-	-	-
**37a**	MAO-B	>100,000	27.4	>3,649	R/C	-	-	-	-	-	10.34	-
**37b**	MAO-B	>100,000	30.5	>3,278	R/C	-	-	-	-	-	6.631	-
**37c**	MAO-B	>100,000	453.2	>220	-	-	-	-	-	-	-	-

Selectivity: determined based on the inhibitor's preference for MAO-A, MAO-B, or NS (non-selective). **SI**: Selectivity index calculated from the ratio IC_{50}, considering IC_{50} MAO-A / IC_{50} MAO-B for MAO-B selective and non-selective inhibitors, and IC_{50} MAO-B / IC_{50} MAO-A for MAO-A selective inhibitors. **Inhibition profile**: **R** = Reversible, **C** = Competitive. **Ki MAO-A/MAO-B**: Inhibition constants for the MAO isoforms. **TPSA (Â^2):** topological polar superficial area; Values < 140 Â^2 indicate good oral absorption; values< 90 Â^2 suggest high probability of penetrating BBB. **LogP**: Lipophylic partition coefficient; ideal values are 0-3 log mol/L. **BBB penetration**: Indicates the ability to cross BBB (Yes or Not). **Toxicity**: Classified as low, moderate, or high. – indicates data not shown in the original paper.

Table S2 - Evaluation of the Druggability profile of Hidrazide and Hidrazone-based analogues.

Hidrazide and Hidrazone-based analogues

Inhibitor	Selectivity	IC_{50} MAO-A (nM)	IC_{50} MAO-B (nM)	SI	Inhibitory profile	Toxicity	Molecular weith	TPSA	LogP	BBB penetration	Ki MAO-B (nM)	Ki MAO-A (nM)
**38**	MAO-B	-	-	0.3744	R/C [b]	-	-	-	-	-	29,660 ± 1,900	79,220 ± 5,030
**39a**	MAO-A	-	-	99	R/C [b]	-	-	-	-	-	990 ± 8	10 ± 1
**39b**	MAO-A	-	-	148	R/C [b]	-	-	-	-	-	1,480 ± 100	10 ± 1
**40a**	MAO-A	6,120	-	-	-	Low	-	-	-	-	-	-
**40b**	MAO-A	6,250	9,300	1.5	-	Low	-	-	-	-	-	-
**41a**	MAO-B	-	-	1.71	R/C [b]	-	-	-	-	-	35.4 ± 2	60.8 ± 4.0
**41b**	MAO-B	-	-	147.11	R/C [b]	-	-	-	-	-	24.2 ± 3	3.560 ± 0.2
**42**	MAO-B	NA	190 ± 10	-	-	-	-	-	-	-	-	-
**43**	MAO-B	1,380 ± 2	1.8 ± 0.3	766.66	R/C [b]	-	-	-	-	-	1,970	5,310
**44a**	MAO-B	8,790 ± 4,760	4.4 ± 1.2	19,977	-	-	-	-	-	-	1,950	1,830
**44b**	MAO-B	420 ± 3	97 ± 2	4.33	R/C [a]	-	-	-	-	-	10,140	2,710
**45**	MAO-B	9,586 ± 2,236	75 ± 9	127.813	NC [b]	Low	379.42	72.18	3.7	Yes	150	-
**46a**	MAO-A	342 ± 15	-	-	R/C [b]	Low	293.41	27.63	6.1	Yes	-	188
**46b**	MAO-A	28 ± 1	-	-	R/C [b]	Low	294.40	105.11	4.76	Yes	-	11
**48a**	MAO-B	1,660 ± 133	1.8 ± 0.12	922	PR [b]	-	-	-	-	-	2.6	-
**48b**	MAO-B	6,470 ± 1,250	2.5 ± 0.15	2,588	-	-	-	-	-	-	-	-
**49**	MAO-B	6,571 ± 296	72.2 ± 5.7	94	IM [b]	-	-	-	-	-	48	-
**50a**	MAO-B	6,630 ± 667	127.4 ± 2.8	51	R/C [b]	-	-	-	-	-	48	-
**50b**	MAO-B	2,670 ± 82	13 ± 1.2	208	R/C [b]	-	-	-	-	-	11	-
**53a**	MAO-B	-	350.03	385.69	-	-	-	-	-	-	-	-

**53b**	MAO-B	-	851.32	117.46	-	-	-	-	-	-	-	-
**55a**	MAO-B	32,711 ± 210	124 ± 15	263.80	R/C [b]	Low	-	-	-	Yes	68 ± 22	-
**55b**	MAO-B	19,176 ± 5,960	82 ± 10	233.85	R/C [b]	Low	-	-	-	Yes	44 ± 2	-
**55c**	MAO-B	22,107 ± 63	104 ± 5	212.57	R/C [b]	Low	-	-	-	Yes	61 ± 1	-
**56a**	MAO-B	>200,000	692	>289	-	Low	-	-	-	-	-	-
**56b**	MAO-B	>200,000	425	>471	-	Low	-	-	-	-	-	-
**56c**	NS	821	710	1.16	-	Low	-	-	-	-	-	-

Selectivity: determined based on the inhibitor's preference for MAO-A, MAO-B, or NS (non-selective). **IC_{50} MAO-A / MAO-B:** 'NA'= not active compounds. **SI**: Selectivity index calculated from the ratio IC_{50}, considering IC_{50} MAO-A / IC_{50} MAO-B for MAO-B selective and non-selective inhibitors, and IC_{50} MAO-B / IC_{50} MAO-A for MAO-A selective inhibitors. **Inhibition profile**: **R** = Reversible, **PR** = Partially reversible, **C** = Competitive, **NC** = Non-competitive, **IM** = Mixed inhibitor; Modes of inhibition related to MAO-A are indicated with **[a]**, and those related to MAO-B with **[b]**. **Ki MAO-A/MAO-B**: Inhibition constants for the MAO isoforms. **TPSA (Â^2):** topological polar superficial area; Values < 140 Â^2 indicate good oral absorption; values< 90 Â^2 suggest high probability of penetrating BBB. **LogP**: Lipophylic partition coefficient; ideal values are 0-3 log mol/L. **BBB penetration**: Indicates the ability to cross BBB (Yes or Not). **Toxicity**: Classified as low, moderate, or high. – indicates data not shown in the original paper.

Table S3 - Evaluation of the Druggability profile of Phthalide, Phthalimide and Indanone derivatives.

Phthalide, Phthalimide and Indanone derivatives

Inhibitor	Selectivity	IC_{50} MAO-A (nM)	IC_{50} MAO-B (nM)	SI	Inhibitory profile	Toxicity	Molecular weith	TPSA	LogP	BBB penetration	Ki MAO-B (nM)	Ki MAO-A (nM)
**60a**	MAO-B	13,200 ± 786	57 ± 10	232	R/C [b]	-	-	-	-	-	-	-
**60b**	MAO-B	16,900 ± 600	45 ± 9	375	R/C [b]	-	-	-	-	-	41	-
**60c**	MAO-B	27,800 ± 4,050	48 ± 12	579	R/C [b]	-	-	-	-	-	-	-
**62**	MAO-B	1,920 ± 172	4.5 ± 0.4	427	QR [b]	-	-	-	-	-	-	-
**63a**	MAO-B	172 ± 10	2.8 ± 0.2	61	-	-	-	-	-	-	-	-
**63b**	MAO-B	96 ± 6	6.2 ± 0.3	16	R [a]	-	-	-	-	-	-	62
**70**	MAO-B	3,920 ± 827	4 ± 1	980	R/C [b]	-	-	-	-	-	4.5	-
**71a**	MAO-B	32 ± 4	2 ± 0.1	16	R [a]	-	-	-	-	-	47	1.7
**71b**	MAO-B	84 ± 9	2 ± 0.03	42	R [a]	-	-	-	-	-	-	-
**71c**	MAO-B	39 ± 1	3 ± 0.5	13	R [a]	-	-	-	-	-	-	-
**72a**	MAO-A	61 ± 6	1,270 ± 596	0.05	-	-	-	-	-	-	-	-
**72b**	MAO-B	4,730 ± 190	26 ± 5	182	-	-	-	-	-	-	-	-
**72c**	MAO-B	183 ± 38	4.4 ± 0.6	42	-	-	-	-	-	-	-	-
**73a**	MAO-B	94,300 ± 8,400	110 ± 10	857	-	-	-	-	-	-	-	-
**73b**	MAO-B	230 ± 20	180 ± 30	1.2	-	-	-	-	-	-	-	-
**73c**	MAO-B	252,200 ± 20,400	270 ± 20	934	-	-	-	-	-	-	-	-
**73d**	MAO-B	436,500 ± 40,300	480 ± 40	909	-	-	-	-	-	-	-	-

Selectivity: determined based on the inhibitor's preference for MAO-A or MAO-B. **SI**: Selectivity index calculated from the ratio IC_{50}, considering IC_{50} MAO-A / IC_{50} MAO-B for MAO-B selective and non-selective inhibitors, and IC_{50} MAO-B / IC_{50} MAO-A for MAO-A selective inhibitors. **Inhibition profile**: **R** = Reversible, **QR:** Quasi-reversible, **C** = Competitive; Modes of inhibition related to MAO-A are indicated with **[a]**, and those related to MAO-B with **[b]**. **Ki MAO-A/MAO-B**: Inhibition constants for the MAO isoforms. **TPSA ($Â^2$):** topological polar superficial area; Values < 140 $Â^2$ indicate good oral absorption; values< 90 $Â^2$ suggest high probability of penetrating BBB. **LogP**: Lipophylic partition coefficient; ideal values are 0-3 log mol/L. **BBB penetration**: Indicates the ability to cross BBB (Yes or Not). **Toxicity**: Classified as low, moderate, or high. – indicates data not shown in the original paper.

Table S4 - Evaluation of the Druggability profile of Chalcones Inhibitors.

Chalcones Inhibitors

Inhibitor	Selectivity	IC_{50} MAO-A (nM)	IC_{50} MAO-B (nM)	SI	Inhibitory profile	Toxicity	Molecular weith	TPSA	LogP	BBB penetration	Ki MAO-B (nM)	Ki MAO-A (nM)
**75**	MAO-B	-	-	0.05	R/C [b]	-	306.28	-	-	-	220	4,320
**76**	MAO-B	-	-	5.42	R/C [b]	-	282.28	-	-	-	900	4,880
**77**	MAO-B	-	-	13.18	R/C [b]	low	-	-	-	yes	110	1,450
**78**	MAO-B	-	-	26.36	R/C [b]	-	-	-	-	-	330	8,700
**79a**	MAO-B	-	-	16	R/C [b]	low	-	-	-	yes	110	1,760
**79b**	MAO-A	-	-	0.13	R/C [b]	low	-	-	-	yes	1,360	180
**80a**	MAO-B	-	-	920	R/C [b]	Low	-	27.117	-	yes	5.0 ± 0.5	4,600 ± 500
**80b**	MAO-B	-	-	630	-	Low	-	-	-	-	14.6 ± 0.1	9,200 ± 1,800
**81**	MAO-B	-	-	-	-	Low	-	17.1	-	yes	875 ± 160	-
**82a**	MAO-B	NA	4.51	>22.173	R [b]	-	-	-	-	-	-	-
**82b**	MAO-B	15,370	11.35	1,354	R [b]	-	-	-	-	-	-	-
**83**	MAO-B	16,100 ± 2,140	67 ± 16	240	Ir/C [b]	-	-	-	-	-	-	-
**84a**	MAO-B	6,293 ± 432	67 ± 2	93.88	R/C [b]	Low	-	-	-	-	32 ± 4	-
**84b**	MAO-B	>40,000	118 ± 36	>338.98	R/C [b]	-	-	-	-	-	45 ± 1	-
**85a**	MAO-B	5,820 ± 720	6.2 ± 0.9	938.7	R/C [b]	-	-	-	-	-	0.78 ± 0.10	-
**85b**	MAO-B	5,230 ± 30	11 ± 1	475.5	R/C [b]	-	-	-	-	-	6.8 ± 1.2	-
**87a**	MAO-B	2,710 ± 140	470 ± 20	5.8	-	-	-	-	-	-	-	-
**87b**	MAO-B	4,180 ± 770	280 ± 30	14.9	-	-	-	-	-	-	-	-
**87c**	MAO-B	51,000 ± 1,720	550 ± 90	92.7	-	-	-	-	-	-	-	-
**87d**	MAO-B	63,200 ± 3,480	350 ± 80	180	-	-	-	-	-	-	-	-

**89a**	MAO-B	131 ± 8	13 ± 2	10	R [a]	-	-	-	-	-	-	-
**89b**	MAO-B	1,050 ± 53	5.3 ± 0.4	198	-	-	-	-	-	-	-	-
**89c**	MAO-B	3,220 ± 171	5.2 ± 1	619	-	-	-	-	-	-	-	-

Selectivity: determined based on the inhibitor's preference for MAO-A or MAO-B. **IC_{50} MAO-A / MAO-B:** 'NA'= not active compounds. **SI**: Selectivity index calculated from the ratio IC_{50}, considering IC_{50} MAO-A / IC_{50} MAO-B for MAO-B selective and non-selective inhibitors, and IC_{50} MAO-B / IC_{50} MAO-A for MAO-A selective inhibitors. **Inhibition profile**: **R** = Reversible, **Ir =** Irreversible, **C** = Competitive; Modes of inhibition related to MAO-A are indicated with **[a]**, and those related to MAO-B with **[b]**. **Ki MAO-A/MAO-B**: Inhibition constants for the MAO isoforms. **TPSA ($Â^2$):** topological polar superficial area; Values < 140 $Â^2$ indicate good oral absorption; values< 90 $Â^2$ suggest high probability of penetrating BBB. **LogP**: Lipophylic partition coefficient; ideal values are 0-3 log mol/L. **BBB penetration**: Indicates the ability to cross BBB (Yes or Not). **Toxicity**: Classified as low, moderate, or high. – indicates data not shown in the original paper.

Table S5 - Evaluation of the Druggability profile of Propargylamine and Phtalonitrile derivatives.

Propargylamine and Phtalonitrile derivatives

Inhibitor	Selectivity	IC_{50} MAO-A (nM)	IC_{50} MAO-B (nM)	SI	Inhibitory profile	Toxicity	Molecular weith	TPSA	LogP	BBB penetration	Ki MAO-B (nM)	Ki MAO-A (nM)
**91a**	MAO-B	3,500	60	58	-	Low	-	-	-	-	-	-
**91b**	MAO-B	3,100	2.3	1,347	-	Low	-	-	-	-	-	-
**92a**	MAO-B	9,970	380	26	R [b]	Low	-	-	-	-	-	-
**92b**	MAO-B	>50,000	510	>100	R [b]	Low	-	-	-	-	-	-
**92c**	MAO-B	23,770	440	55	R [b]	Low	-	-	-	-	-	-
**93**	MAO-A	721	14,600	0,05	R [a]	-	-	-	-	-	-	-
**96a**	MAO-B	218,000	25	8,720	R [b]	-	-	-	-	-	-	-
**96b**	MAO-B	623	14	45	-	-	-	-	-	-	-	-
**97**	MAO-B	55,620	740	75.16	R [b]	-	324.37	90.78	3.37	Yes	-	-

Selectivity: determined based on the inhibitor's preference for MAO-A or MAO-B. **SI**: Selectivity index calculated from the ratio IC_{50}, considering IC_{50} MAO-A / IC_{50} MAO-B for MAO-B selective and non-selective inhibitors, and IC_{50} MAO-B / IC_{50} MAO-A for MAO-A selective inhibitors. **Inhibition profile**: **R** = Reversible; Modes of inhibition related to MAO-A are indicated with **[a]**, and those related to MAO-B with **[b]**. **Ki MAO-A/MAO-B**: Inhibition constants for the MAO isoforms. **TPSA ($\hat{A}^2$):** topological polar superficial area; Values < 140 $\hat{A}^2$ indicate good oral absorption; values< 90 $\hat{A}^2$ suggest high probability of penetrating BBB. **LogP**: Lipophylic partition coefficient; ideal values are 0-3 log mol/L. **BBB penetration**: Indicates the ability to cross BBB (Yes or Not). **Toxicity**: Classified as low, moderate, or high. – indicates data not shown in the original paper.

Table S6 - Evaluation of the Druggability profile of Alkaloids Inhibitors.

Alkaloids Inhibitors

Inhibitor	Selectivity	IC_{50} MAO-A (nM)	IC_{50} MAO-B (nM)	SI	Inhibitory profile	Toxicity	Molecular weith	TPSA	LogP	BBB penetration	Ki MAO-B (nM)	Ki MAO-A (nM)
**99**	MAO-B	2,620	167	15.7	R/C	-	-	-	-	-	-	-
**100**	MAO-B	3,920	86	45	R/C	-	-	-	-	-	-	-
**101a**	MAO-B	924	128	7.2	R	-	-	-	-	-	-	-
**101b**	MAO-B	2,220	61	36	R	-	-	-	-	-	-	-
**103**	MAO-B	-	8,900	-	-	-	-	-	-	-	260	-
**105**	MAO-B	-	47,4	>211	R/C	low	333.3	68.55	-	-	44.1	-
**106a**	MAO-B	1,020 ± 160	80 ± 3	12.75	R	Low	356.466	-	4.424	Yes	-	-
**106b**	MAO-A	120 ± 60	61 ± 40	0.19	R	Low	448.622	-	5.206	Yes	-	-

Selectivity: determined based on the inhibitor's preference for MAO-A or MAO-B. **SI**: Selectivity index calculated from the ratio IC_{50}, considering IC_{50} MAO-A / IC_{50} MAO-B for MAO-B selective and non-selective inhibitors, and IC_{50} MAO-B / IC_{50} MAO-A for MAO-A selective inhibitors. **Inhibition profile**: **R** = Reversible, **C** = Competitive. **Ki MAO-A/MAO-B**: Inhibition constants for the MAO isoforms. **TPSA ($\hat{A}^2$):** topological polar superficial area; Values < 140 $\hat{A}^2$ indicate good oral absorption; values< 90 $\hat{A}^2$ suggest high probability of penetrating BBB. **LogP**: Lipophylic partition coefficient; ideal values are 0-3 log mol/L. **BBB penetration**: Indicates the ability to cross BBB (Yes or Not). **Toxicity**: Classified as low, moderate, or high. – indicates data not shown in the original paper.

Table S7 - Evaluation of the Druggability profile of Benzopyrone Derivatives.

					**Benzopyrone Derivatives**							
Inhibitor	Selectivity	IC_{50} MAO-A (nM)	IC_{50} MAO-B (nM)	SI	Inhibitory profile	Toxicity	Molecular weith	TPSA	LogP	BBB penetration	Ki MAO-B (nM)	Ki MAO-A (nM)
**9108**	MAO-B	NA	140 ± 10	>714	R [b]	-	-	-	-	-	-	-
**109a**	MAO-B	NA	6 ± 0.4	>16,667	R [b]	-	-	-	-	-	-	-
**109b**	MAO-B	1,170 ± 80	3 ± 0.2	360	-	-	-	-	-	-	-	-
**110**	MAO-B	>10,000	8,2 ± 0,82	>1221	-	Low	267.24	76.03	3.57	Yes	-	-
**111a**	MAO-B	NA	760 ± 50	>131.6	R [b]	-	59.31	265.27	2.83	-	-	-
**111b**	MAO-B	NA	21,110 ± 1,420	>4.7	R [b]	-	72.20	266.26	1.60	Yes	-	-
**112a**	MAO-B	NA	0.31 ± 0.02	>333,333	-	-	-	-	-	-	-	-
**112b**	MAO-B	NA	0.8 ± 0.05	>125,000	-	-	-	-	-	-	-	-
**122c**	MAO-B	NA	0.74 ± 0.02	>135,870	-	-	-	-	-	-	-	-
**113**	MAO-B	-	3.1	7244	-	-	-	-	-	-	-	-
**114a**	MAO-A	7 ± 2.1	NA	-	-	-	-	-	-	-	-	-
**114b**	MAO-A	9.1 ± 0.9	17,000 ± 2.4%	-	-	-	-	-	-	-	-	-
**14c**	MAO-A	11 ± 2.8	5,000 ± 0.8%	-	-	-	-	-	-	-	-	-
**115**	MAO-B	15,280 ± 723	13 ± 9	>7,693.31	MI	Low	302.32	39.44	3.17	Yes	3.274	-
**116**	MAO-B	>200,000	3,660 ± 1,640	>100	-	-	-	-	-	-	-	-
**117a**	MAO-B	9,160 ± 610	140 ± 10	65.43	R [b]	-	323.17	50.44	3.79	Yes	-	-
**117b**	MAO-A	6,080 ± 410	21,750 ± 1,460	3.58	-	-	339.17	70.67	3.49	No	-	-
**118**	NS	3,700	3,900	1.05	-	-	-	-	-	-	-	-
**119**	MAO-B	29,720 ± 0,53%	0.37 ± 40	>270,270	R/C [b]	Low	272.08	35.53	-	Yes	-	-
**121a**	MAO-B	NA	69 ± 3	>1,449	-	-	-	-	-	-	-	-

**121b**	MAO-B	NA	68 ± 3	>1,471	-	-	-	-	-	-	-	-
**122a**	MAO-B	4,760 ± 390	64 ± 5.4	74	QR [b]	-	-	-	-	-	-	-
**122b**	MAO-B	NA	63 ± 4.2	>1,585	QR [b]	-	-	-	-	-	-	-
**123**	MAO-B	77,900 ± 25,500	638 ± 387	122	R/C [b]	-	-	-	-	-	940	-
**124**	MAO-B	NA	2.9 ± 1.2	>3,448	-	-	-	-	-	-	-	-
**125**	MAO-B	NA	0.67 ± 0.13	>149,254	R/C [b]	Low	293.32	59.31	3.69	Yes	0.67	-
**127**	MAO-B	36 ± 5.9	1.1 ± 0.2	33	-	-	-	-	-	-	-	-
**128a**	MAO-B	785 ± 47	39 ± 13	20	R/C [a]	-	-	-	-	-	-	1,000
**128b**	MAO-B	1,086 ± 120	7.5 ± 1.1	145	R/C [b]	-	-	-	-	-	6.5	-
**129a**	MAO-B	3,330 ± 101	3.8 ± 0.1	876	-	-	-	-	-	-	-	-
**129b**	MAO-B	286 ± 36	43 ± 0.02	6.7	-	-	-	-	-	-	-	-
**130a**	MAO-B	95 ± 10	0.33 ± 0.001	297.8	R/C [b]	-	-	-	-	-	103	4
**130b**	MAO-B	879 ± 19	2 ± 0.3	440	-	-	-	-	-	-	-	-
**131a**	MAO-B	1,040 ± 3	2.8 ± 0.02	371	QR [b]	-	-	-	-	-	-	-
**131b**	MAO-B	2,200 ± 202	3.7 ± 1.2	595	-	-	-	-	-	-	-	-
**132**	MAO-B	15,800 ± 1,660	4 ± 1.6	3,950	QR [b]	-	-	-	-	-	-	-
**133a**	MAO-A	24 ± 1	78 ± 10	0.3	R/C [a]	-	-	-	-	-	-	25
**133b**	MAO-B	1,290 ± 140	4.5 ± 0.3	287	QR/C [b]	-	-	-	-	-	7.1	-
**134a**	MAO-B	12 ± 2	0.89 ± 0.3	13	R/C [a]	-	-	-	-	-	1.8	19
**134b**	MAO-B	33 ± 6	4.1 ± 0.7	8	-	-	-	-	-	-	-	-
**134c**	MAO-B	34 ± 3	3.5 ± 1	10	-	-	-	-	-	-	-	-
**134d**	MAO-B	26 ± 6	3.1 ± 0.4	8	-	-	-	-	-	-	-	-
**134e**	MAO-B	10 ± 1	1.2 ± 0.4	8	-	-	-	-	-	-	-	-
**136a**	MAO-B	7,980 ± 1,090	2.9 ± 0.9	2,751	R [b]	-	-	-	-	-	2.7	-

**136b**	MAO-B	NA	6.2 ± 0.63	-	-	-	-	-	-	-	-	-
**137**	MAO-B	>10,000	5.4 ± 1.1	>18,519	R/C [b]	-	-	-	-	-	2.6 ± 0.24	-
**138a**	MAO-B	28,900 ± 4,220	1.4 ± 0.3	20,643	-	-	-	-	-	-	-	-
**138b**	MAO-B	>100,000	2.5 ± 0.7	>40,000	-	-	-	-	-	-	-	-
**140a**	MAO-B	9.5 ± 1	6.9 ± 0.6	1.38	-	-	-	-	-	Yes	-	-
**140b**	MAO-A	10 ± 2	76 ± 15	7.6	-	-	-	-	-	-	-	-
**140c**	MAO-B	83 ± 1	4.7 ± 0.8	17.66	-	-	-	-	-	-	-	-
**141**	MAO-A	39,773	92,399	2.32	-	-	-	-	-	-	-	-
**142a**	NS	-	-	-	-	-	284.72	25.25	4.33	Yes	-	-
**142b**	NS	-	-	-	-	-	250.24	25.24	3.84	Yes	-	-
**144a**	MAO-A	2.8	86,000	30,714	-	-	-	-	-	-	-	-
**144b**	MAO-A	2.1	83,000	39,524	-	-	-	-	-	-	-	-
**147**	MAO-B	NA	142 ± 11	>704	R/C [b]	-	-	-	-	-	68	-
**148a**	MAO-B	NA	685 ± 13	>145	R/C [b]	-	-	-	-	-	648	-
**148b**	MAO-B	NA	847 ± 78	>118	-	-	-	-	-	-	-	-
**149a**	MAO-B	44 ± 7	4 ± 1	11	R/C [b]	Low	-	-	-	Yes	3.4 ± 0.4	51 ± 8
**149b**	MAO-B	250 ± 30	8 ± 1	31	-	Low	-	-	-	Yes	7 ± 2	255 ± 25
**150a**	MAO-B	7,430 ± 178	2,540 ± 103	2.9	R/C [b]	-	-	-	-	-	-	6,900
**150b**	MAO-B	24,700 ± 2,360	269 ± 71	91.8	R/C [b]	-	-	-	-	-	160	-

Selectivity: determined based on the inhibitor's preference for MAO-A, MAO-B, or NS (non-selective). **IC_{50} MAO-A / MAO-B:** 'NA'= not active compounds. **SI**: Selectivity index calculated from the ratio IC_{50}, considering IC_{50} MAO-A / IC_{50} MAO-B for MAO-B selective and non-selective inhibitors, and IC_{50} MAO-B / IC_{50} MAO-A for MAO-A selective inhibitors. **Inhibition profile**: **R** = Reversible, **QR:** Quasi-reversible, **MI** = Mixed inhibitor**, C** = Competitive; Modes of inhibition related to MAO-A are indicated with **[a]**, and those related to MAO-B with **[b]**. **Ki MAO-A/MAO-B**: Inhibition constants for the MAO isoforms. **TPSA ($Â^2$):** topological polar superficial area; Values < 140 $Â^2$ indicate good oral absorption; values< 90 $Â^2$ suggest high probability of penetrating BBB. **LogP**: Lipophylic partition coefficient; ideal values are 0-3 log mol/L. **BBB penetration**: Indicates the ability to cross BBB (Yes or Not). **Toxicity**: Classified as low, moderate, or high. – indicates data not shown in the original paper.

Table S8 - Evaluation of the Druggability profile of Benzyloxy-based Analogues.

Benzyloxy-based Analogues

Inhibitor	Selectivity	IC_{50} MAO-A (nM)	IC_{50} MAO-B (nM)	SI	Inhibitory profile	Toxicity	Molecular weith	TPSA	LogP	BBB penetration	Ki MAO-B (nM)	Ki MAO-A (nM)
**153**	MAO-B	>100,000	9 ± 1	110,000	C [b]	-	-	-	-	-	6.8	-
**154**	MAO-B	>100,000	12.34 ± 1.62	>8,104	-	Low	272.28	35.53	3.656	Yes	-	-
**155**	MAO-B	189 ± 12	3 ± 1	63	R/C [b]	-	-	-	5.61	-	3.9 ± 0.5	560 ± 7
**156a**	MAO-B	6,460 ± 1,140	39 ± 2	166		-	-	-	-	-	-	-
**156b**	MAO-B	3,650 ± 150	85 ± 16	43		-	-	-	-	-	-	-
**156c**	MAO-B	3,520 ± 770	105 ± 32	34		-	-	-	-	-	-	-
**157**	MAO-B	17,000 ± 890	2,950 ± 90	6	MI [b]	-	487.96	79.03	6.29	-	-	-
**159a**	MAO-B	NA	41 ± 1	-	R/NC [b]	Low	504.04	104.57	3.865	-	36	-
**159b**	MAO-B	NA	65 ± 2	-	R/NC [b]	Low	518.06	104.57	4.155	-	55	-
**160**	MAO-B	-	3.9 ± 0.7	>25,641	-	-	-	-	-	-	-	-
**161a**	MAO-B	50,700 ± 4,450	2.9 ± 0.3	17,482	-	-	-	-	-	-	-	-
**161b**	MAO-B	17,700 ± 2,940	1.3 ± 0.3	13,615	-	-	-	-	-	-	-	-
**161c**	MAO-B	38,200 ± 3,130	4 ± 1	9,550	-	-	-	-	-	-	-	-
**162**	MAO-B	NA	8,320 ± 540	>12	-	-	-	-	-	-	-	-
**163a**	MAO-A	5,030 ± 928	7,870 ± 647	1.6	Ir [a]/PR [b]	-	-	-	-	-	-	-
**163b**	MAO-B	13,200 ± 2,400	3,690 ± 457	3.6	Ir [a]/PR [b]	-	-	-	-	-	-	-
**165a**	MAO-B	16,200	270	60	R/C [b]	-	-	-	-	Yes	320	-
**165b**	MAO-B	55,900	460	122	R/C [b]	-	-	-	-	Yes	890	-

Selectivity: determined based on the inhibitor's preference for MAO-A or MAO-B. **IC_{50} MAO-A / MAO-B:** 'NA'= not active compounds. **SI**: Selectivity index calculated from the ratio IC_{50}, considering IC_{50} MAO-A / IC_{50} MAO-B for MAO-B selective and non-selective inhibitors, and IC_{50} MAO-B / IC_{50} MAO-A for MAO-A selective inhibitors. **Inhibition profile**: **R** = Reversible, **Ir** = Irreversible, **PR** = Partially reversible, **C** = Competitive, **NC** = Non-competitive, **MI** = Mixed inhibitor; Modes of inhibition related to MAO-A are indicated with **[a]**, and those related to MAO-B with **[b]**. **Ki MAO-A/MAO-B**: Inhibition constants for the MAO isoforms. **TPSA (Â^2):** topological polar superficial area; Values < 140 Â^2 indicate good oral absorption; values< 90 Â^2 suggest high probability of penetrating BBB. **LogP**: Lipophylic partition coefficient; ideal values are 0-3 log mol/L. **BBB penetration**: Indicates the ability to cross BBB (Yes or Not). **Toxicity**: Classified as low, moderate, or high. – indicates data not shown in the original paper.

Table S9 - Evaluation of the Druggability profile of Azole-based Derivatives.

Azole-based Derivatives

Inhibitor	Selectivity	IC_{50} MAO-A (nM)	IC_{50} MAO-B (nM)	SI	Inhibitory profile	Toxicity	Molecular weith	TPSA	LogP	BBB penetration	Ki MAO-B (nM)	Ki MAO-A (nM)
**164**	MAO-A	1 ± 0.1	510 ± 20	510	-	-	-	-	-	-	-	-
**165a**	MAO-B	990 ± 130	210 ± 10	4.7	-	-	-	-	-	-	-	-
**165b**	MAO-B	930 ± 20	200 ± 10	4.6	-	-	-	-	-	-	-	-
**166**	MAO-B	-	103 ± 18	>175	R/C	-	-	-	-	-	-	-
**168**	MAO-B	NA	11 ± 5	-	C	Low		61.69	2.66	Yes	21 ± 1	-
**169**	MAO-B	46,200 ± 11,200	2.7 ± 0.64	17,111	R	-	-	-	-	-	-	-
**170a**	MAO-B	NA	7.61 ± 0.64	-	R	-	-	-	-	Yes	-	-
**170b**	MAO-B	NA	19.35 ± 0.68	-	R	-	-	-	-	Yes	-	-
**171a**	MAO-B	15,400 ± 1,980	900 ± 50	16.7	C	-	-	-	-	-	0,35	-
**171b**	MAO-A	3750 ± 212	3,980 ± 518	1.1	C	-	-	-	-	-	-	1,3
**171c**	MAO-B	12,900 ± 14	371 ± 35	34.8	-	-	-	-	-	-	-	-
**172a**	MAO-B	1,110 ± 7	12 ± 0.61	92	-	-	-	-	-	-	-	-
**172b**	MAO-B	751 ± 23	411 ± 11	1.8	-	-	-	-	-	-	-	-
**173a**	MAO-B	NA	5.3 ± 0.3	-	R/C	Low	305.338	67.49	3.715	Yes	-	-
**173b**	MAO-B	NA	6.8 ± 0.2	-	R/C	Low	337.337	96.95	2.452	Yes	-	-

**174**	MAO-B	NA	5.3 ± 0.3	-	R/C	Low	305.338	67.49	3.715	Yes	-	-
**175a**	MAO-A	28 ± 3.9	1,400 ± 87	50	-	-	-	-	-	-	-	-
**175b**	MAO-B	>100,000	763 ± 35	131.062	-	-	-	-	-	Yes	-	-
**176a**	MAO-A	3 ± 0.7	29,500 ± 4,680	9,833		-	-	-	-	-	-	-
**176b**	MAO-B	8,180 ± 1,940	20 ± 1	409	R/C	-	-	-	-	-	-	-
**177a**	MAO-A	-	-	0.000055	R/C	-	-	-	-	-	72,120 ± 1,330	4 ± 1
**177b**	MAO-A	-	-	0.003	R/C	-	-	-	-	-	1,900 ± 120	5 ± 1
**178**	MAO-B	>100,000	12,200 ± 2,300	>8.2	R	-	-	-	-	-	-	-
**179**	MAO-B	NA	221 ± 28	271.1	-	-	-	-	-	-	-	-
**180**	MAO-B	NA	11.97 ± 0.37	>8,354	-	-	-	-	-	-	-	-
**182**	MAO-B	21,460 ± 70	50 ± 3	477	R/C	-	-	-	-	-	13.2 ± 1.4	-
**183a**	MAO-B	-	1,372,800 ± 48,400	-	-	-	-	-	-	-	-	-
**183b**	MAO-B	-	26,500 ± 1,300	-	-	-	-	-	-	-	-	-
**184**	MAO-B	46,365 ± 1,506	19 ± 1	2,440	-	-	-	-	-	-	-	-
**185a**	MAO-A	2,640	77,180	29.23	-	-	-	-	-	-	-	-
**185b**	MAO-B	-	41,470	-	-	-	-	-	-	-	-	-
**186**	MAO-B	26,014 ± 1,983	67.3 ± 1.5	387	R/C	Low	-	-	-	Yes	82,50	-
**187a**	MAO-B	19,000 ± 1,200	17 ± 4.5	1,138	R/C	-	-	-	-	-	6.2	-
**187b**	MAO-B	61,000 ± 9,190	98 ± 3.6	622	-	-	-	-	-	-	-	-

Selectivity: determined based on the inhibitor's preference for MAO-A or MAO-B. **IC_{50} MAO-A / MAO-B:** 'NA'= not active compounds. **SI**: Selectivity index calculated from the ratio IC_{50}, considering IC_{50} MAO-A / IC_{50} MAO-B for MAO-B selective and non-selective inhibitors, and IC_{50} MAO-B / IC_{50} MAO-A for MAO-A selective inhibitors. **Inhibition profile**: **R** = Reversible, **C** = Competitive. **Ki MAO-A/MAO-B**: Inhibition constants for the MAO isoforms. **TPSA (Â^2):** topological polar superficial area; Values < 140 Â^2 indicate good oral absorption; values< 90 Â^2 suggest high probability of penetrating BBB. **LogP**: Lipophylic partition coefficient; ideal values are 0-3 log mol/L. **BBB penetration**: Indicates the ability to cross BBB (Yes or Not). **Toxicity**: Classified as low, moderate, or high. – indicates data not shown in the original paper.

Table S10 - Evaluation of the Druggability profile of Diverse Molecular Hybrids.

Diverse Molecular Hybrids

Inhibitor	Selectivity	IC_{50} MAO-A (nM)	IC_{50} MAO-B (nM)	SI	Inhibitory profile	Toxicity	Molecular weith	TPSA	LogP	BBB penetration	Ki MAO-B (nM)	Ki MAO-A (nM)
**190a**	MAO-B	-	10 ± 3	-	R	Low	-	-	-	Yes	-	-
**190b**	MAO-B	-	10 ± 5	-	Ir	Low	-	-	-	Yes	-	-
**190c**	MAO-B	-	20 ± 5	-	R	Low	-	-	-	Yes	-	-
**191**	MAO-B	58,190 ± 1,970	2,780 ± 110	20.93	-	Low	-	-	-	-	-	-
**192**	MAO-B	>100,000	60 ± 4	1,666.67	R	Low	-	-	-	Yes	-	-
**193a**	MAO-B	NA	950 ± 350	>105.26	-	-	-	-	-	-	-	-
**193b**	MAO-B	NA	3,970 ± 380	25.18	-	-	-	-	-	-	-	-
**194**	MAO-B	>10,000	760 ± 80	>13.16	-	-	323.32	83.83	2.97	-	-	-
**195a**	MAO-B	>40,000	110 ± 24	>363.6	R/C	-	-	-	-	-	-	-
**195b**	MAO-B	4,210 ± 980	100 ± 13	42.1	R/C	-	-	-	-	-	-	-
**196**	MAO-A	5,950 ± 830	6,520 ± 2	0.91	-	-	-	-	-	-	-	-
**197a**	MAO-B	-	42 ± 2	>23.812	R/NC	Low	-	-	-	-	-	-
**197b**	MAO-B	-	56 ± 2	>17.857	-	Low	-	-	-	-	35	-
**200**	MAO-B	15,220 ± 3,400	32 ± 2	475	-	Low	-	-	-	-	46	-
**203a**	MAO-A	194 ± 7	652 ± 27	3.36	-	-	455.58	45.55	4.84	Yes	-	-
**203b**	MAO-A	188 ± 7	479 ± 20	2.55	MI	Low	473.57	45.55	5.00	Yes	-	3.4
**203c**	MAO-B	1,485 ± 56	330 ± 14	0.22	-	-	423.51	45.55	4.05	Yes	-	-
**204a**	MAO-B	3,857 ± 770	203 ± 4	19.04	R/C	-	464.14	-	-	Yes	155 ± 50	-
**204b**	MAO-B	6,149 ± 305	979 ± 206	6.67	R/C	-	455.22	-	-	Yes	721 ± 74	-
**204c**	MAO-A	3,691 ± 655	5,336 ± 667	0.69	-	-	458.26	-	-	-	-	-

**206a**	MAO-B	36,920 ± 11	12,700 ± 14	2.9	-	-	375.36	164.69	-	-	-	-
**206b**	MAO-B	32,160 ± 27	18,870 ± 10	1.7	-	-	359.29	181.76	-	-	-	-
**206c**	MAO-B	37,300 ± 6	19,560 ± 95	1.9	-	-	301.25	138.13	-	-	-	-
**206d**	MAO-A	13,100 ± 12	46,110 ± 3	0.28	-	-	420.37	159.24	-	-	-	-
**206e**	MAO-A	16,030 ± 11	51,880 ± 16	0.3	-	-	317.25	146.87	-	-	-	-
**206f**	MAO-A	19,360 ± 73	42,140 ± 7	0.45	-	-	316.25	152.67	-	-	-	-

Selectivity: determined based on the inhibitor's preference for MAO-A or MAO-B. **IC_{50} MAO-A / MAO-B:** 'NA'= not active compounds. **SI**: Selectivity index calculated from the ratio IC_{50}, considering IC_{50} MAO-A / IC_{50} MAO-B for MAO-B selective and non-selective inhibitors, and IC_{50} MAO-B / IC_{50} MAO-A for MAO-A selective inhibitors. **Inhibition profile**: **R** = Reversible, **Ir** = Irreversible, **C** = Competitive, **NC** = Non-competitive, **MI** = Mixed inhibitor. **Ki MAO-A/MAO-B**: Inhibition constants for the MAO isoforms. **TPSA (Â^2):** topological polar superficial area; Values < 140 Â^2 indicate good oral absorption; values< 90 Â^2 suggest high probability of penetrating BBB. **LogP**: Lipophylic partition coefficient; ideal values are 0-3 log mol/L. **BBB penetration**: Indicates the ability to cross BBB (Yes or Not). **Toxicity**: Classified as low, moderate, or high. – indicates data not shown in the original paper.

Table S11 - Evaluation of the Druggability profile of Dual Cholinesterase and MAO Inhibitors.

Dual Cholinesterase and MAO Inhibitors

Inhibitor	Selectivity	IC_{50} MAO-A (nM)	IC_{50} MAO-B (nM)	SI	IC_{50} AChE (nM)	IC_{50} BuChE (nM)	Inhibitory profile	Toxicity	TPSA	LogP	BBB penetration
**212**	MAO-B / AChE	4,520 ± 32	59 ± 2	76.61	8.7 ± 0.2	-	R/C [a],[b] MI [c]	Low	-	-	-
**213a**	MAO-B / AChE	3,990 ± 218	101 ± 8.9	39.5	47%	6.35%	-	-	-	-	Yes
**213b**	MAO-B / AChE	2,660 ± 51	5.3 ± 0.8	501.88	44%	12.77%	R/C	-	-	-	Yes
**213c**	MAO-B / AChE	29,100 ± 2,720	7.2 ± 1.8	4,041	41%	6.22%	-	-	-	-	Yes
**215a**	MAO-B / AChE	5,164 ± 131	890 ± 96	5.8	52 ± 6	960 ± 5	R [b] R/C [c]	Low	-	-	-
**215b**	MAO-B / AChE	1,730 ± 87	34 ± 7	50.88	850 ± 107	880 ± 7	R [b] R/C [c]	Low	-	-	-
**217a**	MAO-B	-	84 ± 3	-	-	-	R [b]	Low	-	-	-
**217b**	MAO-B / AChE	-	111 ± 6	-	15,170 ± 6,030	-	R [b]	Low	-	-	-
***E*-218**	MAO-B / AChE	15,000 ± 1,000	479 ± 81	31.3	174 ± 17	-	IM [c]	-	-	-	-
***E/Z*-218**	MAO-B / AChE	2,980 ± 80	743 ± 48	4.1	295 ± 45	-	-	-	-	-	-
***E*-219**	MAO-B / AChE	13,000 ± 6,000	355 ± 134	36.6	39 ± 1	7,000 ± 10	IM [c	Low	-	4.71	Yes
***E/Z*-219**	MAO-B / AChE	39,000 ± 5,000	358 ± 8	108.9	53 ± 6	10,000 ± 320	-	-	-	-	-
**220a**	MAO-B / AChE	29,400 ± 1,220	2,720 ± 320	10.8	8,770 ± 200	-	-	-	-	-	-
**220b**	MAO-B / AChE	31,400 ± 3,500	650 ± 23	48.3	28,000 ± 2,430	36,400 ± 2,360	R/C [b]	-	32.34	-	Yes
**220c**	MAO-B / AChE	34,900 ± 4,100	710 ± 3.5	49.2	26,300 ± 1,290	36,200 ± 3,650	R/C [b]	-	32.34	-	Yes
**221a**	MAO-B	>100,000	250 ± 20	>400	>100,000	>100,000	-	Low	59.31	3.52	-
**221b**	MAO-B	>100,000	310 ± 20	>322.58	-	-	R [b]	Low	59.31	3.94	Yes
**221c**	MAO-B	-	1,590 ± 110	62.89	>100,000	>100,000	-	Low	59.31	3.16	Yes
**221d**	MAO-A / AChE	78,160 ± 5,280	>100,000	>0.78	3,780 ± 250	>100,000	-	Low	59.31	2.28	-
**222a**	MAO-B	>40,000	5,480 ± 770	>7.30	-	-	R/C [b]	Low	-	-	-

**22b**	AChE	-	-	-	12,900 ± 2,580	-	-	-	-	-	-
**224**	MAO-A / AChE	7.7 ± 0.7	7,900 ± 1,340	1,025	520 ± 70	44,900 ± 6,100	-	-	-	-	-
**225a**	BuChE	>100,000	>100,000	-	>100,000	223 ± 5	-	Low	-	-	Yes
**225b**	-	6,420 ± 10,500	7,830 ± 780	1.2	>100,000	56,300 ± 10,500	-	Low	-	-	Yes

Selectivity: determined based on the inhibitor's preference for MAO-A or MAO-B and AChE or BuChE. **IC_{50} MAO-A / MAO-B** and **AChE / BuChE:** 'NA'= not active compounds. **SI**: Selectivity index calculated from the ratio IC_{50}, considering IC_{50} MAO-A / IC_{50} MAO-B for MAO-B selective and non-selective inhibitors, and IC_{50} MAO-B / IC_{50} MAO-A for MAO-A selective inhibitors. **Inhibition profile**: **R** = Reversible, **C** = Competitive, **MI** = Mixed inhibitor; Modes of inhibition related to MAO-A are indicated with **[a]**, and those related to MAO-B with **[b],** and to AChE with **[c]**.**TPSA (Â^2):** topological polar superficial area; Values < 140 Â^2 indicate good oral absorption; values< 90 Â^2 suggest high probability of penetrating BBB. **LogP**: Lipophylic partition coefficient; ideal values are 0-3 log mol/L. **BBB penetration**: Indicates the ability to cross BBB (Yes or Not). **Toxicity**: Classified as low, moderate, or high. – indicates data not shown in the original paper.

Table S12 - Evaluation of the Druggability profile of Dual Adenosine Receptors Antagonists and MAO Inhibitors.

Dual Adenosine Receptors Antagonists and MAO Inhibitors

Inhibitor	Selectivity	IC_{50} MAO-A (nM)	IC_{50} MAO-B (nM)	SI	Inhibitory profile	Ki MAO-B (nM)	Ki A_1 (nM)	Ki A_{2A} (nM)	Ki A_{2B} (nM)	Ki A_3 (nM)	Toxicity	BBB penetration
**227a**	MAO-B / A_1	>10,000	197 ± 25	50.76	-	-	791 ± 110	1,510 ± 31	>1,000	>1,000	-	Yes
**227b**	MAO-B / A	>10,000	508	19.68	-	-	217 ± 64	268 ± 75	>1,000	>300	-	-
**229**	MAO-B / A_1	>10,000	210 ± 41	47.62	-	-	393 ± 101	595 ± 51	-	-	-	-
**230a**	MAO-B	>10,000	62.9 ± 7	158.98	-	-	-	-	>1,000	>10,000	-	-
**230b**	MAO-B / A_{2A}	>10,000	1,800 ± 160	5.56	-	-	605 ± 157	417 ± 68	>1,000	4,410 ± 710	-	-
**232**	MAO-B / A_1	>10,000	106 ±	94.34	-	-	396 ± 154	1,620 ± 280	>1,000	>10,000	-	-
**234**	MAO-B / A_{2A}	>10,000	570 ± 18	175.44	-	-	1,550 ± 310	189 ± 47	>1,000	>1,000	-	-
**235**	MAO-B / A_{2A}	34%	242.67 ± 45.32		-	-	>1,000	264 ± 106	>1,000	>1,000	-	-
**236**	MAO-B / A_{2A}	-	-	-	-	290 ± 30	-	330 ± 90	-	-	Low	Yes
**237**	MAO-B / A_{2A}	-	-	-	-	630 ± 110	-	850 ± 110	-	-	Low	Yes
**238**	MAO-B / A_{2A}	10,000	200	50	-	-	-	260	-	-	-	-
**239a**	MAO-B / A_1	1,630	20	81.5	-	-	369 ± 20	NA	-	-	-	-
**239b**	MAO-B / A_1	273	7.4	36.89	-	-	676 ± 190	NA	-	-	-	-
**240**	MAO-B / A_{2A}	>10,000	34.9 ± 2.5	286.53	R/C	-	2,500 ± 660	39.5 ± 5.8	>1,000	>1,000	-	-

Selectivity: determined based on the inhibitor's preference for MAO-A or MAO-B and A_1, A_{2A}, A_{2B} or A_3. **SI**: Selectivity index calculated from the ratio IC_{50}, considering IC_{50} MAO-A / IC_{50} MAO-B for MAO-B selective and non-selective inhibitors, and IC_{50} MAO-B / IC_{50} MAO-A for MAO-A selective inhibitors. **Inhibition profile**: **R** = Reversible, **C** = Competitive. **Ki MAO-B** and **A_1/A_{2A}/A_{2B}/A_3**: Inhibition constants for MAO-B and adenosine receptors; 'NA'= not active compounds. **BBB penetration**: Indicates the ability to cross BBB (Yes or Not). **Toxicity**: Classified as low, moderate, or high. – indicates data not shown in the original paper.

Table S13 - Evaluation of the Druggability profile of Dual MAO and Catechol O-methyltransferase Inhibitors.

Dual MAO and Catechol O-methyltransferase (COMT) Inhibitors

Inhibitor	Selectivity	IC_{50} MAO-A (nM)	IC_{50} MAO-B (nM)	SI	Inhibitory profile	Toxicity	Molecular weith	TPSA	BBB penetration	Ki COMT (nM)
**243**	MAO-B / COMT	76,100 ± 4,130	13,900 ± 890	5.5	R/C	-	-	-	-	290 ± 10
**244**	COMT	95,580 ± 1,710	58,820 ± 4,950	-	-	Low	-	-	Yes	-
**245**	COMT	76,600 ± 7,430	83,200 ± 14,500	0.92	-	-	-	-	-	48 ± 3
**246**	COMT	41,400 ± 3,090	42,100 ± 4,140	1.02	-	-	-	-	-	230 ± 50
**247**	MAO-B / COMT	46,000 ± 5,260	7,260 ± 650	6.34	-	-	-	-	-	570 ± 50
**248**	MAO-B / COMT	37,400 ± 980	7,830 ± 630	4.78	-	-	-	-	-	420 ± 180
**250a**	MAO-B	NA	2,500 ± 220	>4	-	Low	273.3	60.77	Yes	1,650 ± 10
**250b**	MAO-B	NA	4,270 ± 300	>2.3	-	Low	298.3	84.56	Yes	1,330 ± 30
**250c**	MAO-B	NA	4,550 ± 310	>2.2	-	Low	-	-	-	2,410 ± 60
**250d**	MAO-B	NA	4,380 ± 510	>2.3	-	Low	-	-	-	1,270 ± 110

Selectivity: determined based on the inhibitor's preference for MAO-A, MAO-B, or COMT. **IC_{50} MAO-A / MAO-B:** 'NA'= not active compounds. **SI**: Selectivity index calculated from the ratio IC_{50}, considering IC_{50} MAO-A / IC_{50} MAO-B for MAO-B selective and non-selective inhibitors, and IC_{50} MAO-B / IC_{50} MAO-A for MAO-A selective inhibitors. **Inhibition profile**: **R** = Reversible, **C** = Competitive. **TPSA (Â^2):** topological polar superficial area; Values < 140 Â^2 indicate good oral absorption; values< 90 Â^2 suggest high probability of penetrating BBB. **BBB penetration**: Indicates the ability to cross BBB (Yes or Not). **Toxicity**: Classified as low, moderate, or high. – indicates data not shown in the original paper.

Table S14 - Evaluation of the Druggability profile of Caspase Inhibitors.

Caspase Inhibitors

Inhibitor	Selectivity	IC_{50} MAO-A (nM)	IC_{50} MAO-B (nM)	SI	Inhibitory profile	Toxicity	Molecular weith	TPSA	LogP	BBB penetration	Ki MAO-B (nM)	Ki MAO-A (nM)
252a	MAO-B	22,750	8,320	2.73	R	-	-	-	-	Yes	3,580	6,000
252b	MAO-B	8,260	5,960	1.38	R	-	-	-	-	Yes	2,570	2,180

Selectivity: determined based on the inhibitor's preference for MAO-A or MAO-B. **SI:** Selectivity index calculated from the ratio IC_{50} MAO-A/IC_{50} MAO-B. **Inhibition profile**: **R** = Reversible. **Ki MAO-A/MAO-B**: Inhibition constants for the MAO isoforms. **TPSA ($\hat{A}^2$):** topological polar superficial area; Values < 140 $\hat{A}^2$ indicate good oral absorption; values< 90 $\hat{A}^2$ suggest high probability of penetrating BBB. **LogP**: Lipophylic partition coefficient; ideal values are 0-3 log mol/L. **BBB penetration**: Indicates the ability to cross BBB (Yes or Not). **Toxicity**: Classified as low, moderate, or high. – indicates data not shown in the original paper.
